# Fatty acid species differentially regulate macrophage polarization and oxidative stress with secondary effects on macrophage–HSC crosstalk

**DOI:** 10.14814/phy2.71082

**Published:** 2026-09-10

**Authors:** Koichi Nakanishi, Hiroji Shinkawa, Shigekazu Takemura, Kanako Nakagawa, Yukiko Minamiyama, Takeaki Ishizawa

**Affiliations:** ^1^ Department of Hepato‐Biliary‐Pancreatic Surgery Osaka Metropolitan University Osaka Japan; ^2^ Department of Frontier Life‐Science, Graduate School of Medicine Osaka Metropolitan University Osaka Japan; ^3^ Food Hygiene and Environmental Health Division of Applied Life Science, Graduate School of Life and Environmental Sciences Kyoto Prefectural University Kyoto Japan

**Keywords:** hepatic stellate cells, lipid species, macrophage polarization, metabolic dysfunction–associated steatohepatitis, PPARγ

## Abstract

Metabolic dysfunction–associated steatohepatitis (MASH) is characterized by lipid accumulation, inflammation, and fibrosis. Macrophage–hepatic stellate cell (HSC) communication plays a key role in fibrogenesis; however, how fatty acid species influence macrophage polarization remains unclear. Bone marrow‐derived macrophages (BMDMs) were polarized toward M1 or M2 phenotypes in the presence of palmitic acid (PA), oleic acid (OA), or palmitoleic acid (PO). Polarization markers, reactive oxygen species (ROS), and peroxisome proliferator–activated receptor gamma (PPARγ) expression were analyzed by RT‐qPCR, Western blotting, and chemiluminescence. Primary HSCs were exposed to fatty acids or conditioned media from treated macrophages. PA enhanced selected inflammatory macrophage responses and increased ROS production under M1‐polarizing conditions. OA enhanced ROS production in M1 macrophages and suppressed several M2‐associated markers, whereas PO reduced inflammatory responses and ROS under M1‐polarizing conditions. Direct fatty acid exposure did not significantly alter HSC activation markers. Conditioned media from polarized macrophages, particularly M1 macrophages, reduced α‐SMA and cytoglobin protein expression in HSCs, while fatty acid‐specific effects were modest and marker‐dependent. Fatty acid species differentially modulate macrophage polarization and redox activity, partly in association with PPARγ signaling. These macrophage changes may secondarily affect macrophage–HSC communication and stellate cell redox‐related responses, providing insight into lipid regulation of immune‐stromal interactions in the liver.

## Graphical abstract

Fatty acid species differentially regulate macrophage polarization and ROS production. These macrophage changes secondarily influence HSC redox‐related markers, without evidence of direct fatty acid‐induced HSC activation.

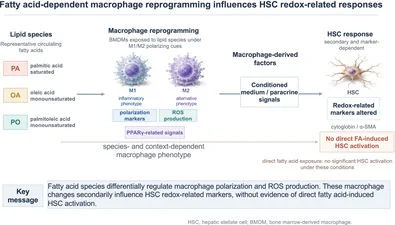

## 1 INTRODUCTION

Metabolic dysfunction–associated steatohepatitis (MASH) is a progressive form of metabolic dysfunction–associated steatotic liver disease (MASLD) characterized by hepatic steatosis, hepatocellular injury, chronic inflammation, and fibrosis (Eslam et al., 2020). In Japan, several million individuals are estimated to be affected, and disease progression is strongly associated with metabolic abnormalities such as obesity, diabetes mellitus, and dyslipidemia. Persistent inflammation in MASH promotes excessive extracellular matrix deposition, ultimately leading to cirrhosis, hepatocellular carcinoma, and liver failure.

In MASLD, excessive lipid accumulation within the liver plays a central role in disease progression. Saturated and unsaturated fatty acids released from expanded adipose tissue induce inflammatory signaling and oxidative stress in hepatocytes and non‐parenchymal cells (Tilg & Moschen, 2010), driving the transition from simple steatosis to MASH. Adipose tissue inflammation further increases circulating free fatty acids and pro‐inflammatory cytokines (Hotamisligil, 2006), amplifying hepatic inflammation. However, the mechanisms by which specific fatty acids contribute to fibrogenic processes remain incompletely understood.

Liver fibrosis is primarily mediated through interactions between macrophages and hepatic stellate cells (HSCs) (Takemura et al., 2018). Macrophages are highly plastic innate immune cells that orchestrate inflammatory and reparative responses. In response to environmental cues, resting macrophages (M0) polarize toward pro‐inflammatory M1 or anti‐inflammatory M2 phenotypes. M1 macrophages, induced by stimuli such as lipopolysaccharide and interferon‐γ, produce inflammatory cytokines including tumor necrosis factor‐α (TNF‐α) and express inducible nitric oxide synthase (iNOS) (Devaraj & Jialal, 2011). In contrast, interleukin‐4 and interleukin‐13 drive M2 polarization, characterized by expression of peroxisome proliferator–activated receptor‐γ (PPARγ), transforming growth factor‐β (TGF‐β) (Devaraj & Jialal, 2011; Kodai et al., 2007; Lee et al., 2011), and other markers associated with tissue repair and fibrosis (Sun et al., 2017). Dysregulation of macrophage polarization is a key determinant of chronic inflammatory diseases, including MASH (Xuan et al., 2015).

HSCs reside in the space of Disse and remain quiescent under physiological conditions, storing vitamin A–containing lipid droplets. Upon chronic liver injury, HSCs become activated, lose lipid droplets, express α‐smooth muscle actin (α‐SMA), and produce extracellular matrix components such as type I collagen (Friedman, 2008). Macrophage‐derived cytokines and oxidative stress are critical drivers of this activation process, highlighting the importance of macrophage–HSC crosstalk in fibrosis progression.

Palmitic acid (PA), oleic acid (OA), and palmitoleic acid (PO) are abundant circulating fatty acids that are transported bound to albumin. Previous studies have shown that PA promotes inflammatory macrophage activation via TLR4‐dependent pathways, whereas OA and PO can exert context‐dependent effects on inflammatory signaling depending on cellular context and activation state (Chan et al., 2015; Shi et al., 2006; Song et al., 2015). A recent human macrophage study further suggested that fatty acid composition, rather than total lipid exposure alone, can influence macrophage polarization through PPARγ‐related signaling (AlSaeed et al., 2026). However, most studies have examined the effects of fatty acids on naïve macrophages, and little is known about how individual fatty acids modulate macrophages actively undergoing cytokine‐driven polarization or how these changes influence macrophage–HSC communication.

Therefore, this study aimed to examine how representative fatty acid species modulate macrophage polarization and influence macrophage–HSC communication under defined conditions.

## 2 MATERIALS AND METHODS

### 2.1 Reagents and media

Bone marrow–derived macrophages (BMDMs) and hepatic stellate cells were cultured in high‐glucose Dulbecco's modified Eagle's medium (DMEM, 4500 mg/L; Wako, Osaka, Japan) supplemented with 10% fetal bovine serum (FBS; Biowest, Nuaillé, France) and 100 U/mL penicillin and 100 μg/mL streptomycin (Wako). Accutase (Innovative Cell Technologies/Funakoshi) was used to detach adherent cells.

For macrophage polarization, lipopolysaccharide (LPS; *E. coli* 055:B5; Difco), interferon‐γ (IFN‐γ; Cat. No. RCY‐359; ITSI‐Biosciences, LLC, Johnstown, PA, USA), interleukin‐4 (IL‐4; Cat. No. 400‐04), and interleukin‐13 (IL‐13; Cat. No. 400‐16) (all from Peprotech, Rocky Hill, NJ, USA) were used.

Palmitic acid (PA; Cat. No. P0500; Sigma‐Aldrich, St. Louis, MO), oleic acid (OA; Cat. No. 151‐03403; FUJIFILM Wako), and palmitoleic acid (PO; Cat. No. P9417; Sigma‐Aldrich) were used as representative saturated and unsaturated fatty acids. Fatty acid–free bovine serum albumin (BSA; Cat. No. 015‐23871; FUJIFILM Wako) and ethanol (99.5%; FUJIFILM Wako) were used to prepare BSA–fatty acid complexes.

Total RNA was extracted using the NucleoSpin RNA kit (Cat. No. 740955.25; MACHEREY‐NAGEL, Düren, Germany) and reverse‐transcribed using the ReverTra Ace qPCR RT Kit (Cat. No. FSQ‐101; TOYOBO, Osaka, Japan). RT‐qPCR was performed with THUNDERBIRD Probe qPCR Mix (Cat. No. QPS‐101; TOYOBO) on a 7500 Fast Real‐Time PCR System (Applied Biosystems).

Reactive oxygen species (ROS) production was assessed using L‐012 (Cat. No. 120‐14,891; Wako, Osaka, Japan), phorbol 12‐myristate 13‐acetate (PMA; Cat. No. 16561‐29‐8; Sigma‐Aldrich), and superoxide dismutase (SOD; Cat. No. 190‐08771; Wako).

### 2.2 Animals

Male Wistar rats were purchased from Japan SLC, Inc. (Shizuoka, Japan). All animal procedures were approved by the Animal Care and Use Committee of Osaka Metropolitan University (Approval No. 21002) and were conducted in accordance with institutional guidelines and the ARRIVE guidelines. Rats were euthanized under sevoflurane anesthesia by intraperitoneal injection of ethyl carbamate (urethane; 4 mL/kg of 25% solution; FUJIFILM Wako). Bone marrow–derived macrophages (BMDMs) and hepatic stellate cells were isolated from rats as described in Sections 2.1.3 and 2.1.5, respectively.

### 2.3 Isolation of bone marrow cells and culture of macrophages

Bone marrow cells were isolated from the femurs and tibias of male Wistar rats (6–12 weeks old) by flushing with sterile PBS(−). After red blood cell lysis and filtration, cells were resuspended in high‐glucose DMEM containing 10% FBS and antibiotics and adjusted to 1.5 × 10^6^ cells/mL.

Cells were seeded into 75‐cm^2^ flasks and cultured for 7 days at 37°C in 5% CO_2_ in the presence of rat recombinant granulocyte–macrophage colony‐stimulating factor (GM‐CSF, 15 ng/mL; Cat. No. 400‐23; Peprotech) to generate BMDMs.

### 2.4 Macrophage polarization and fatty acid treatment

After 7 days, BMDMs were detached with Accutase, collected, and seeded into 6‐well plates (Cat. No. 353046; Corning, NY, USA) at 1.0 × 10^5^ cells/well in 2 mL complete medium. After 24 h for adherence, macrophages were polarized as follows:

- M0: unstimulated controls
- M1: LPS (5 ng/mL) + IFN‐γ (1 ng/mL)
- M2: IL‐4 (10 ng/mL) + IL‐13 (10 ng/mL)

PA, OA, or PO was dissolved in ethanol (500 mM stock) and conjugated to BSA (1.7 mM) to obtain 10 mM BSA–fatty acid solutions (2% ethanol). These were added to the culture medium at a final 50‐fold dilution, giving final fatty acid concentrations of 0, 0.1, or 0.2 mM (34 μM BSA, 0.04% ethanol).

Macrophages were incubated with fatty acids and polarization stimuli for 3, 6, or 24 h. Cells were then harvested for protein analysis (western blot) or RNA extraction (RT‐qPCR), as indicated in each experiment. Successful polarization was confirmed by the expected induction of iNOS/TNF‐α (M1) and PPARγ/Ym1 (M2).

### 2.5 Isolation and culture of hepatic stellate cells

Hepatic stellate cells were isolated from male Wistar rats (6–12 weeks old) by in situ liver perfusion via the portal vein with calcium‐free buffer followed by enzyme‐containing buffers (pronase E and collagenase) at 42°C–44°C. After perfusion and additional digestion, liver cell suspensions were filtered and stellate cells were enriched by Nycodenz density gradient centrifugation in Gey's balanced salt solution, as described previously with minor modifications (Takemura et al., 2018).

The stellate cell–enriched fraction was collected, washed, and resuspended in high‐glucose DMEM with 10% FBS and antibiotics. Cells were first plated in 6‐cm dishes for 1 h to allow Kupffer cells to adhere, and the non‐adherent stellate cells were then collected and seeded into 6‐well plates at 0.35–0.45 × 10^6^ cells/mL.

### 2.6 Stimulation of hepatic stellate cells with fatty acids and conditioned media

Stellate cells were cultured for 3 days after isolation before stimulation. Fresh medium containing BSA‐conjugated PA, OA, or PO was added to achieve final fatty acid concentrations of 0 or 0.2 mM (34 μM BSA, 0.04% ethanol).

In separate experiments, stellate cells were treated with conditioned media from fatty acid–treated polarized macrophages. Culture supernatants from macrophages stimulated for 24 h (Section 2.1.4) were collected, clarified by centrifugation, and mixed 1:1 with fresh stellate cell medium (total 2 mL/well).

After 3 days of stimulation, stellate cells were harvested for protein extraction or RNA isolation.

### 2.7 Protein extraction and western blot analysis

Macrophages and stellate cells were washed with PBS(−) and lysed in ice‐cold whole‐cell lysis buffer containing HEPES, NaCl, EDTA, EGTA, dithiothreitol, Tween‐20, glycerol, Na_3_VO_4_, NaF, and phenylmethylsulfonyl fluoride. Lysates were clarified by centrifugation, and supernatants were stored at −80°C until use.

Protein concentrations were determined using a BCA Protein Assay Kit (Cat. No. 23225; Thermo Fisher Scientific, Waltham, MA) with BSA standards, and absorbance was measured at 562 nm using a microplate reader (SPECTRA MAX 190; Molecular Devices).

Equal amounts of protein were mixed with SDS sample buffer, boiled, and separated by SDS‐PAGE (7.5%–15% gels). Proteins were transferred to PVDF membranes (Cat. No. IPVH08130; Merck Millipore, Burlington, MA), blocked with commercial blocking buffer (Can Get Signal; Cat. No. NKB‐101; TOYOBO), and incubated with primary antibodies against iNOS/NOS2, TNF‐α, mannose receptor (MR), PPARγ, α‐smooth muscle actin (α‐SMA), cytoglobin, or β‐actin (loading control). A rabbit polyclonal anti‐cytoglobin antibody was kindly provided by Prof. Norifumi Kawada (Osaka Metropolitan University, Japan). β‐actin was used as a loading control.

After incubation with HRP‐conjugated secondary antibodies, bands were visualized using enhanced chemiluminescent substrates (Immobilon Western, Cat. No. WBKLS; Merck Millipore, Burlington, MA or Trident Fento‐ECL, Cat. No. GTX14698; GeneTex, Irvine, CA) and detected with a LuminoGraph 1 (ATTO) or Fusion Solo S (Vilber Lourmat). The target proteins, antibody host/clone information, suppliers, catalogue numbers, lot numbers, working dilutions, and corresponding HRP‐conjugated secondary antibodies are summarized in Table S1.

Original images of the available membrane sections corresponding to the representative blots shown in the main figures are provided in File S1. Molecular‐weight marker positions visible in the original images, migration positions of the target proteins and β‐actin, sample lanes, and the regions used for presentation in the main figures are indicated.

Original images of the available membrane sections from all additional independent experiments included in the densitometric analyses are provided in Figure S2. For each blot, the corresponding main‐figure panel, target protein, visible molecular‐weight marker positions, sample order, experimental date, and bands included in the quantitative analysis are indicated. Where present in the original blots, additional control lanes without BSA vehicle or fatty‐acid treatment are labeled “No BSA/no FA” in File S1.

During the original experiments, the membranes were cut horizontally according to the expected molecular‐weight ranges of the target proteins before antibody incubation; therefore, full‐length images of the uncut membranes are not available. The images provided represent the complete original images available for each membrane section.

β‐Actin was used as the loading control. Depending on the experiment, β‐actin was detected either by reprobing the same membrane section after target‐protein detection or on a separately run gel and membrane using aliquots of the same protein samples. The loading‐control procedure used for each blot is explicitly indicated in the Supplementary Materials.

### 2.8 RNA extraction and quantitative real‐time PCR

Total RNA was extracted from macrophages and stellate cells (0 or 0.2 mM fatty acid) using the NucleoSpin RNA kit, including on‐column DNase treatment, according to the manufacturer's protocol. Total RNA was eluted in RNase‐free water and quantified using a NanoDrop 2000c spectrophotometer.

cDNA was synthesized from total RNA using the ReverTra Ace qPCR RT Kit (10‐μL reactions; 37°C for 15 min, 95°C for 5 min) on a thermal cycler (TaKaRa PCR Thermal Cycler MP; Takara Bio), and diluted to 5–10 ng/μL.

RT‐qPCR was performed in 20‐μL reactions containing cDNA (final 0.5–1 ng/μL), THUNDERBIRD Probe qPCR Mix, ROX reference dye, and either commercial TaqMan Gene Expression Assays or custom primer–probe sets (Sigma‐Aldrich). GAPDH or β‐actin was used as the endogenous control for RT‐qPCR analyses, depending on the experiment, as specified in each figure legend.

Amplification was carried out on a 7500 Fast Real‐Time PCR System using 40 cycles of 95°C for 3 s and 60°C for 30 s. Custom‐designed primer–probe sets were used for GAPDH, TGF‐β1, β‐actin, Col1a1, and α‐SMA, whereas commercially available TaqMan Gene Expression Assays were used for NOS2, TNF‐α, PPARγ, Ym1, and cytoglobin. The sequences of the custom‐designed primers and probes and the assay IDs for the commercial TaqMan assays are provided in Table S2.

### 2.9 Chemiluminescence assay for superoxide production

BMDMs were seeded into white 96‐well plates at 2.0 × 10^5^ cells/100 μL/well and allowed to adhere for 24 h. Cells were then treated with fatty acids and polarized to M0, M1, or M2 as described above.

After 24 h of polarization, cells were washed with PBS(−) and incubated with Krebs–HEPES buffer (100 μL/well). Basal chemiluminescence was recorded for 30 min using a SpectraMax i3x plate reader; the mean value of the last 5 min was taken as background.

L‐012 was added to a final concentration of 100 μM, and chemiluminescence was recorded for another 30 min; the mean of the last 5 min was defined as the base superoxide (O_2_·^−^) level. PMA (10 μM) was then added as a protein kinase C activator, and chemiluminescence was monitored for 30 min; the maximal value was defined as the peak O_2_·^−^ production.

O_2_·^−^ production was calculated as peak minus base values after background subtraction. In selected wells, SOD (1000 U/mL) was added to confirm that the signal reflected O_2_·^−^ generation.

### 2.10 Data analysis

Western blot bands were quantified using CS Analyzer (ATTO) and ImageJ (NIH). Band intensities were normalized to β‐actin or other appropriate loading controls.

Statistical analyses were performed using Statistical Analysis System software (Esumi Co., Ltd., Tokyo, Japan). Data are presented as mean ± SE unless otherwise indicated. For comparisons among multiple groups, one‐way ANOVA followed by the Tukey–Kramer post hoc test was used. For conditioned medium experiments with matched preparations, repeated‐measures one‐way ANOVA followed by the Tukey–Kramer post hoc test was applied. *p* < 0.05 was considered statistically significant.

## 3 RESULTS

### 3.1 Effects of individual fatty acids on macrophage polarization

To examine how different fatty acids influence cytokine‐driven macrophage polarization, we analyzed representative M1 and M2 markers in the presence of PA, OA, or PO.

**FIGURE 1:**
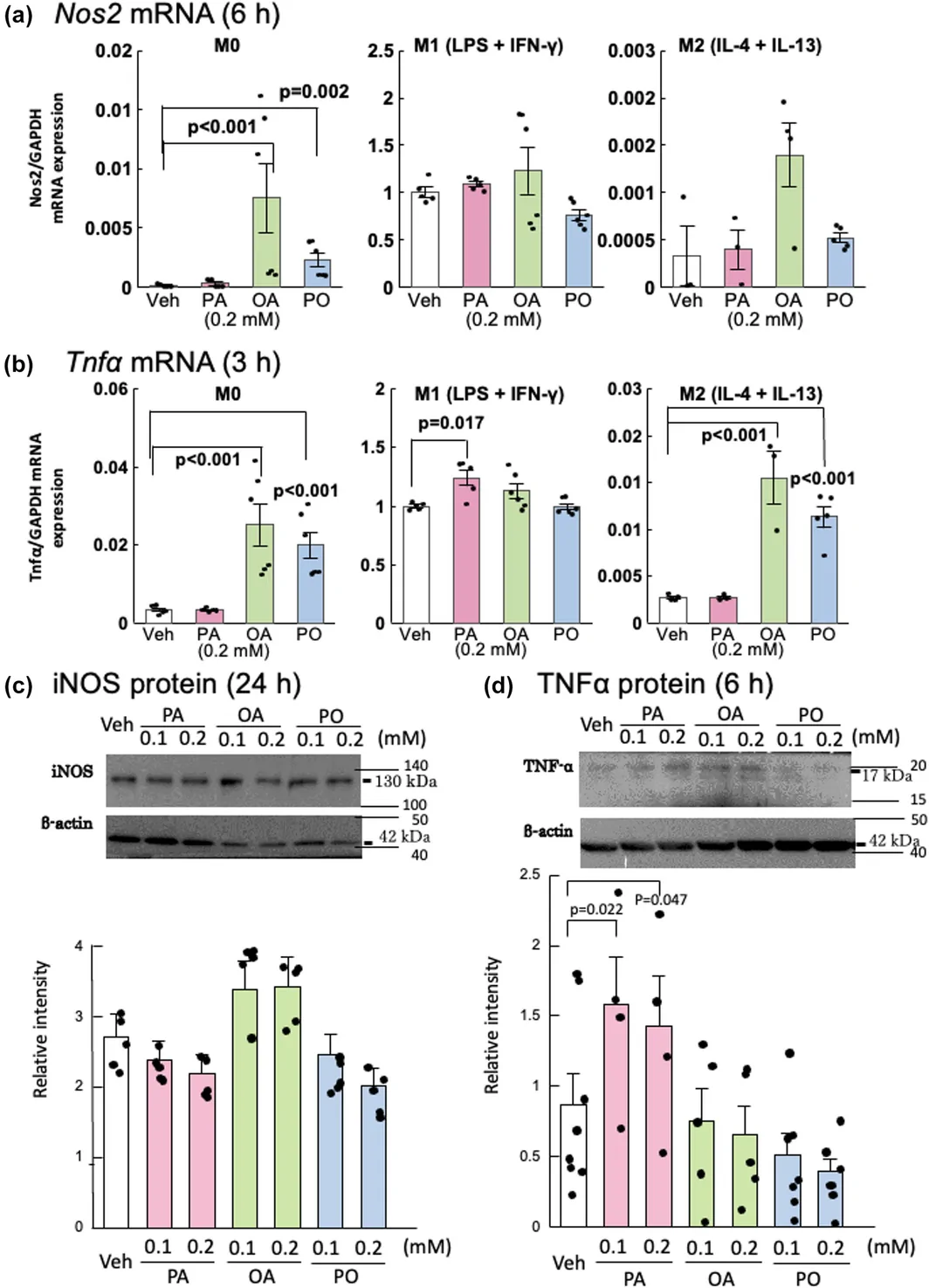
Effects of free fatty acids on macrophage polarization toward the M1 phenotype. Bone marrow–derived macrophages (BMDMs) were treated with palmitic acid (PA), oleic acid (OA), or palmitoleic acid (PO) (0–0.2 mM), followed by stimulation with LPS (5 ng/mL) plus IFN‐γ (1 ng/mL) or IL‐4 (10 ng/mL) plus IL‐13 (10 ng/mL) for the indicated times. Macrophages were classified as M0 (unstimulated), M1 (LPS + IFN‐γ), or M2 (IL‐4 + IL‐13). (a, b) mRNA expression of Nos2 and Tnf was analyzed by RT‐qPCR and normalized to GAPDH using the 2^−ΔΔCt^ method. Data are presented as mean ± SE (*n* = 3–6). (c, d) Protein expression of iNOS and TNF‐α was assessed by western blot and normalized to β‐Actin. Representative immunoblots and densitometric quantification are shown. The TNF‐α and β‐Actin images were obtained from separate gels and membranes. The β‐Actin blot was generated using the same sample set and was used for normalization of TNF‐α expression. The sample order was identical between the TNF‐α and β‐Actin blots. Representative blots are provided in File S1, and additional blots from independent experiments included in the densitometric analyses are provided in Figure S2. Data are presented as mean ± SE (*n* = 3–6). Exact *p* values vs. the corresponding vehicle control are shown in the figure; values smaller than 0.001 are indicated as *p* < 0.001. Statistical analysis was performed using one‐way ANOVA followed by Tukey's multiple comparison test. Horizontal brackets indicate the exact groups compared.

The M1 markers Nos2/iNOS and Tnf/TNF‐α were robustly induced in M1 macrophages, whereas iNOS and TNF‐α proteins were undetectable in M0 and M2 macrophages. In M1 macrophages, PA significantly increased Tnf mRNA expression and increased iNOS and TNF‐α protein expression, while changes in Nos2 mRNA were modest (Figure 1a–d). In M0 and M2 macrophages, OA and PO increased Nos2 and Tnf mRNA expression in some comparisons; however, the magnitude of induction was much lower than that induced by LPS plus IFN‐γ (Figure 1a,b), and iNOS and TNF‐α proteins remained undetectable (data not shown).

**FIGURE 2:**
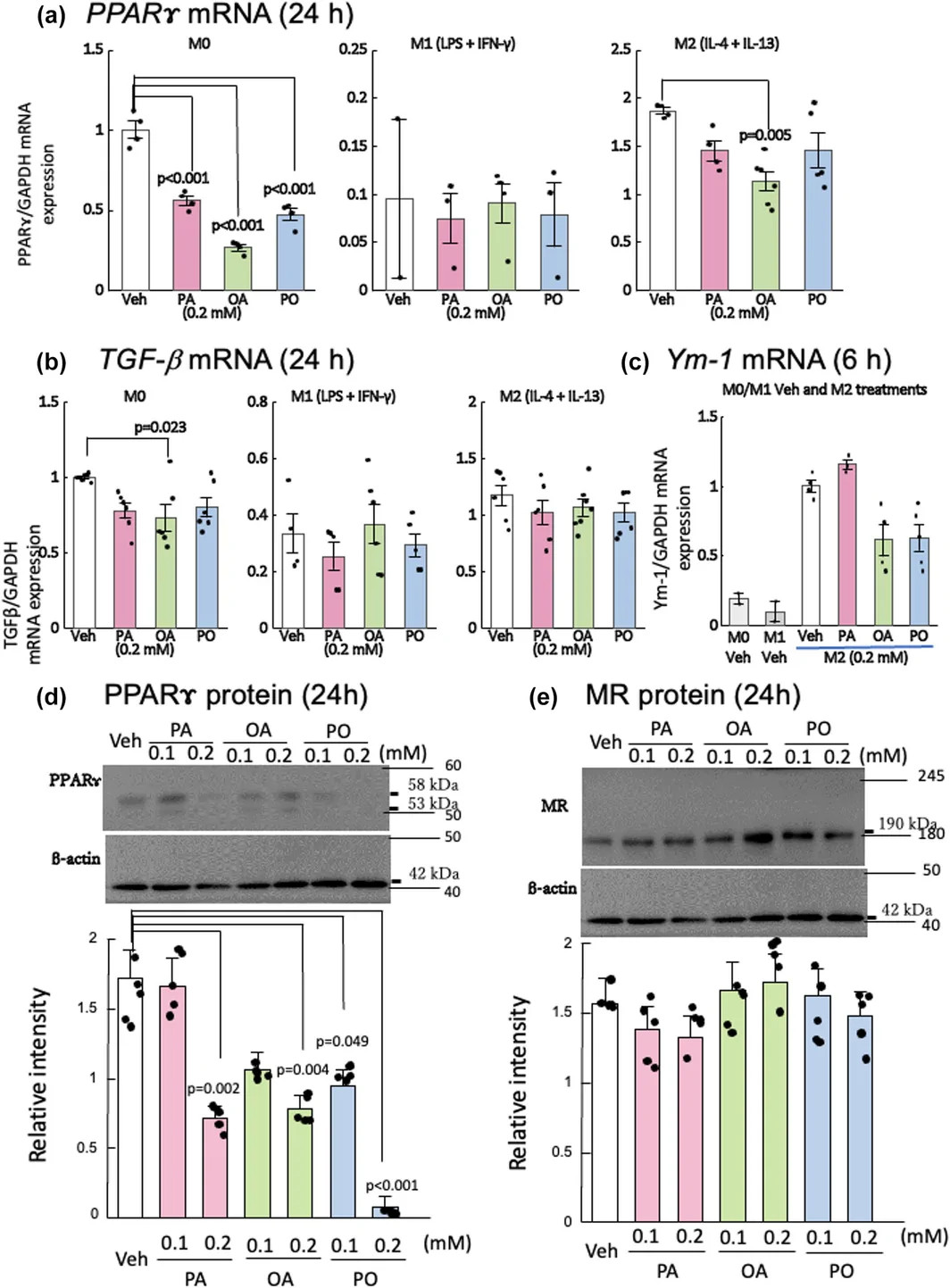
Effects of free fatty acids on macrophage polarization toward the M2 phenotype. Bone marrow–derived macrophages (BMDMs) were treated with BSA (vehicle) or BSA‐conjugated free fatty acids (palmitic acid [PA], oleic acid [OA], or palmitoleic acid [PO], 0.1 or 0.2 mM), followed by stimulation with LPS (5 ng/mL) plus IFN‐γ (1 ng/mL) or IL‐4 (10 ng/mL) plus IL‐13 (10 ng/mL) for 6 or 24 h, as indicated. (a–c) mRNA expression levels of M2 markers PPARγ, TGF‐β, and Ym1 were quantified by RT‐qPCR. Expression levels were normalized to GAPDH and calculated using the 2^−ΔΔCt^ method. Data are presented as mean ± SE (*n* = 3–6). (d, e) After 24 h of stimulation, protein expression levels of PPARγ and mannose receptor (MR) were determined by western blot analysis. Representative immunoblots are shown, and band intensities were quantified and normalized to β‐Actin as a loading control. Representative uncropped blots are provided in File S1, and additional uncropped blots from independent experiments included in the densitometric analyses are provided in Figure S2. Data are presented as mean ± SE (*n* = 3–6). Exact *p* values vs. the corresponding vehicle control are shown in the figure; values smaller than 0.001 are indicated as *p* < 0.001. Statistical analysis was performed using one‐way ANOVA followed by Tukey's multiple comparison test. Horizontal brackets indicate the exact groups compared.

The M2 marker PPARγ was strongly expressed at both the mRNA and protein levels in M2 macrophages, whereas PPARγ protein was not detected in M0 and M1 macrophages. In M2 macrophages, PPARγ expression was reduced by fatty acid treatment at selected concentrations, with the most consistent suppression observed for PA and OA in the protein analysis (Figure 2a,d). TGF‐β and Ym1 mRNA expression also showed fatty acid‐dependent changes, whereas MR protein expression was not significantly altered by any fatty acids tested (Figure 2b,c,e).

**FIGURE 3:**
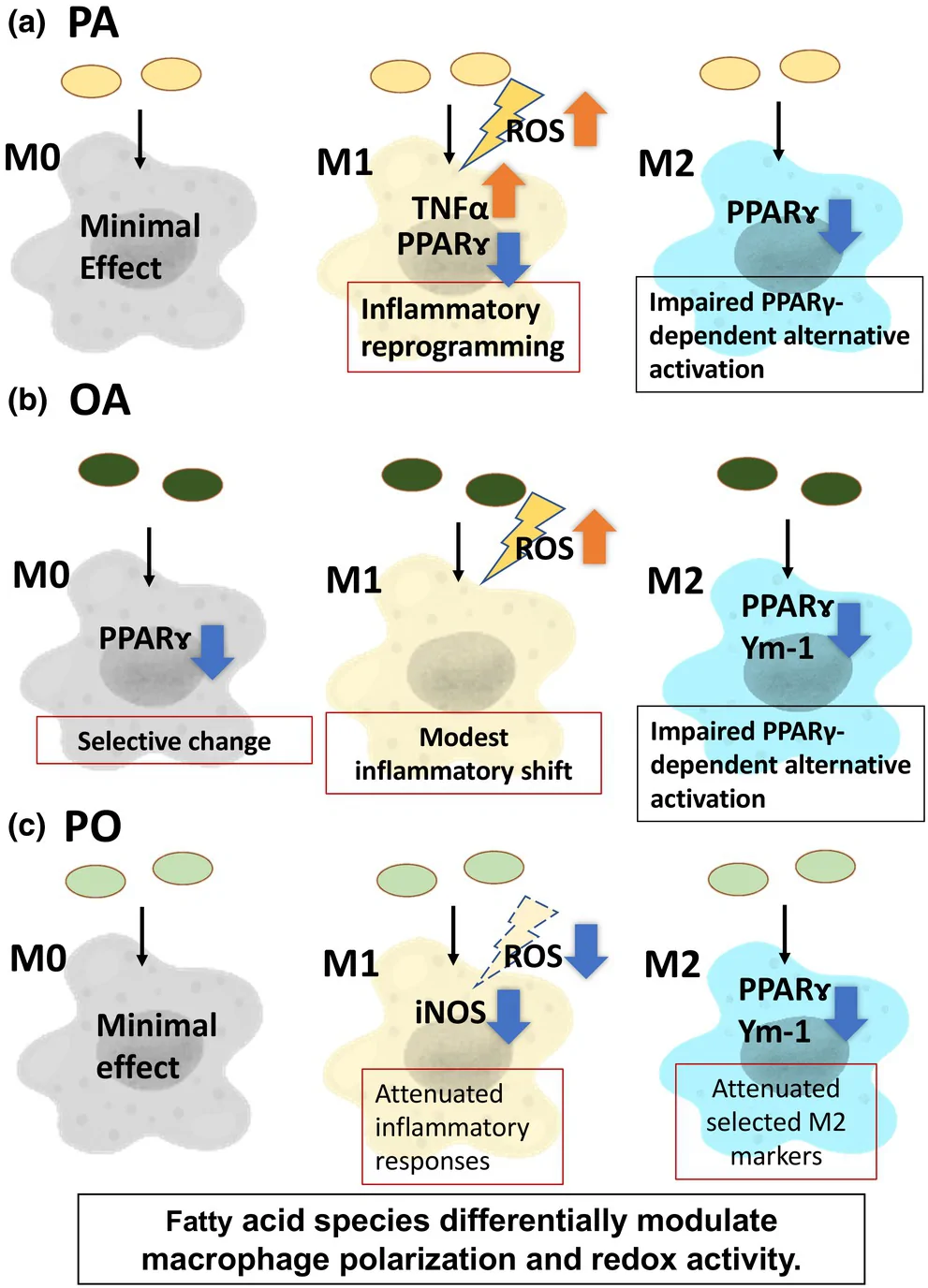
Schematic summary of fatty acid species‐dependent macrophage reprogramming. Palmitic acid (PA), oleic acid (OA), and palmitoleic acid (PO) differentially modulate macrophage polarization and redox activity under M0, M1, and M2 conditions. (a) PA enhanced inflammatory features under M1‐polarizing conditions, with increased TNFα and ROS and reduced PPARγ, while impairing PPARγ‐dependent alternative activation under M2‐polarizing conditions. (b) OA showed selective effects, including reduced PPARγ in M0 macrophages, increased ROS under M1‐polarizing conditions, and reduced PPARγ and Ym‐1 under M2‐polarizing conditions. (c) PO had minimal effects in M0 macrophages, attenuated inflammatory responses under M1‐polarizing conditions, and reduced selected M2‐associated markers. Overall, fatty acid species differentially modulate macrophage polarization and redox activity, which may secondarily influence macrophage–HSC crosstalk in a marker‐dependent manner.

These findings are summarized in Figure 3. Overall, PA reinforced M1‐associated inflammatory features, OA enhanced ROS production and suppressed several M2‐associated markers, and PO attenuated M1‐associated responses while also reducing selected M2‐associated markers under alternative activation conditions.

### 3.2 Effects of individual fatty acids on ROS production in polarized macrophages

We next investigated the impact of fatty acids on inflammatory activity by measuring ROS production in polarized macrophages in the presence of PA, OA, or PO.

**FIGURE 4:**
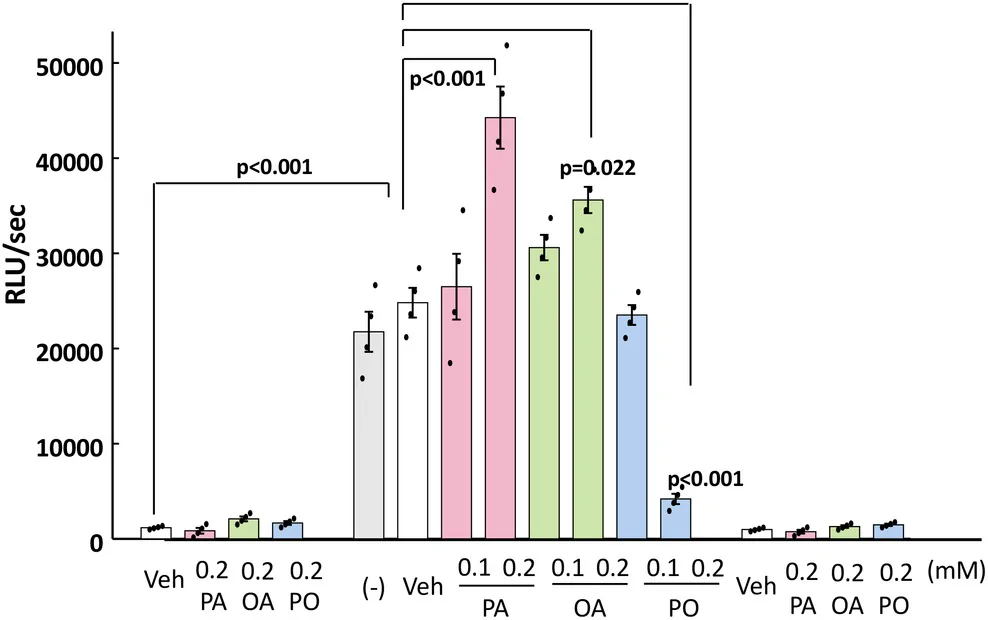
Reactive oxygen species (ROS) production in polarized macrophages treated with free fatty acids. Bone marrow–derived macrophages (BMDMs) were treated with 0.1 or 0.2 mM BSA‐conjugated free fatty acids (palmitic acid, oleic acid, or palmitoleic acid) and stimulated for 24 h under the following conditions: unstimulated (M0), LPS (5 ng/mL) plus IFN‐γ (1 ng/mL) (M1 polarization), or IL‐4 (10 ng/mL) plus IL‐13 (10 ng/mL) (M2 polarization). Intracellular ROS production was measured using the chemiluminescent probe L‐012 and quantified by chemiluminescence assay. Data are presented as mean ± SE (*n* = 3–6). Exact *p* values are shown in the figure when applicable; values smaller than 0.001 are indicated as *p* < 0.001. Statistical analysis was performed using one‐way ANOVA followed by Tukey's multiple comparison test. Horizontal brackets indicate the exact groups compared.

Chemiluminescence measurements using L‐012 revealed that ROS production was markedly higher in M1 macrophages than in M0 or M2 macrophages (Figure 4). In M0 and M2 macrophages, none of the fatty acids significantly affected ROS production. In M1 macrophages, there was no difference in ROS levels between non‐treated cells and vehicle‐treated cells, indicating that BSA itself did not alter ROS production. In contrast, the addition of 0.2 mM PA or OA significantly increased ROS production, whereas 0.2 mM PO significantly decreased it. Thus, PA and OA enhanced the inflammatory activity of M1 macrophages, while PO exerted a suppressive effect.

### 3.3 Direct effects of individual fatty acids on hepatic stellate cell activation

To determine whether fatty acids directly modulate hepatic stellate cell activation, we examined the expression of activation markers and fibrosis‐related genes in stellate cells cultured in the presence of PA, OA, or PO.

**FIGURE 5:**
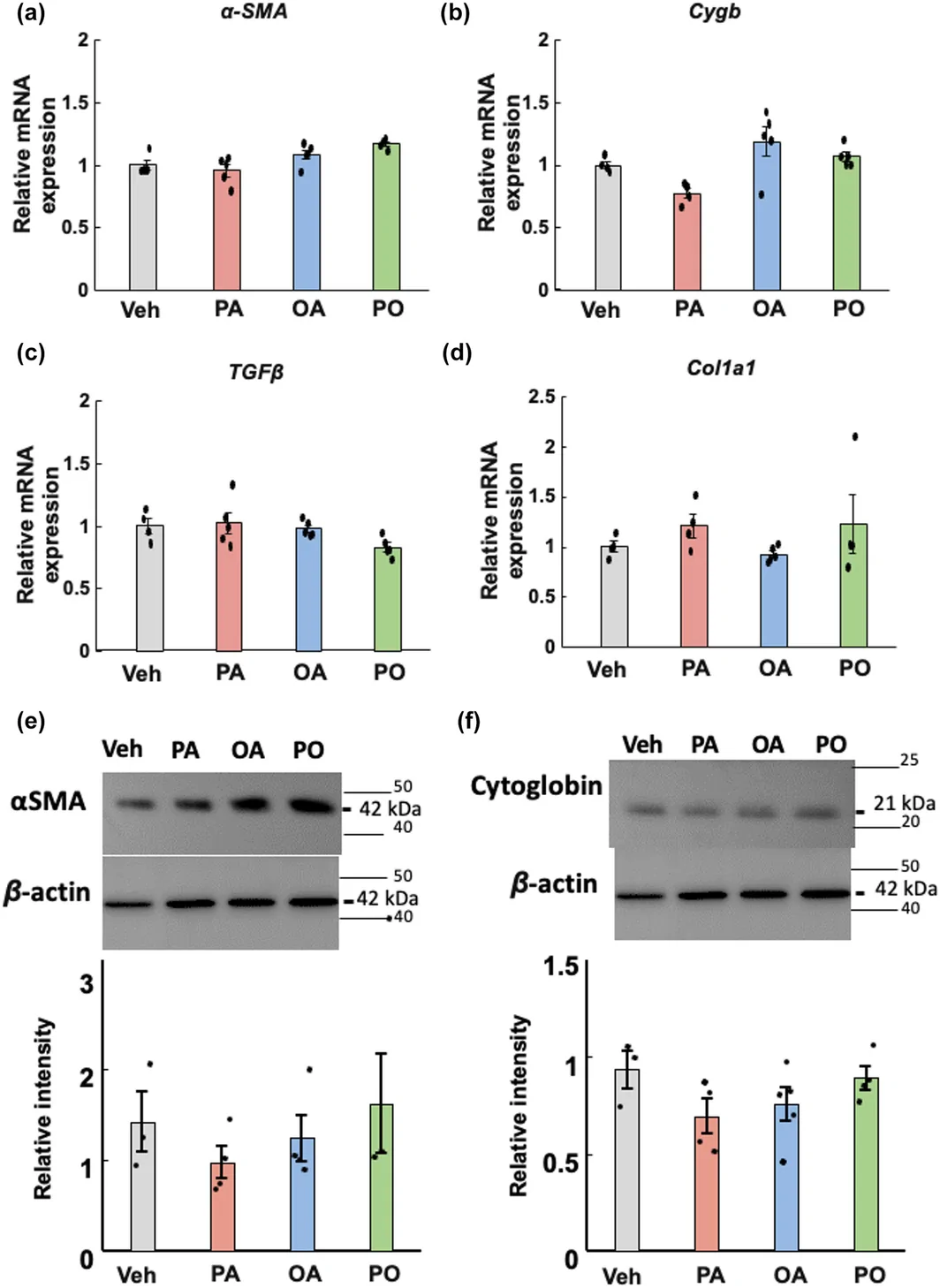
Direct effects of free fatty acids on activation and fibrosis‐related markers in hepatic stellate cells. Primary hepatic stellate cells (HSCs) were isolated and cultured for 3 days. Cells were then treated with BSA (vehicle) or BSA‐conjugated free fatty acids (palmitic acid [PA], oleic acid [OA], or palmitoleic acid [PO], 0.2 mM) and further incubated for 3 days. (a–d) mRNA expression levels of the activation marker α‐SMA and fibrosis‐related factors cytoglobin, TGF‐β, and Col1a1 were quantified by RT‐qPCR. Expression levels were normalized to β‐Actin and calculated using the 2^−ΔΔCt^ method. (e, f) Protein expression levels of α‐SMA and cytoglobin were determined by Western blot analysis. Representative immunoblots are shown, and band intensities were quantified and normalized to β‐Actin as a loading control. Data are presented as mean ± SE from independent experiments (*n* = 3–6 for mRNA analyses and *n* = 3–5 for protein analyses). Statistical analysis was performed using one‐way ANOVA followed by Tukey's multiple comparison test. No significant differences were observed. Representative uncropped blots are provided in File S1, and additional uncropped blots from independent experiments included in the densitometric analyses are provided in Figure S2.

mRNA expression of α‐smooth muscle actin (α‐SMA), a marker of stellate cell activation, was not altered by any of the fatty acids (Figure 5a). Likewise, cytoglobin, an inhibitory factor for stellate cell activation, showed no change in mRNA expression (Figure 5b). mRNA levels of TGF‐β and Col1a1, encoding the pro‐α1 chain of type I collagen, were also unaffected. At the protein level, neither α‐SMA nor cytoglobin differed significantly between vehicle‐treated and fatty acid–treated cells.

These results indicate that none of the tested fatty acids directly induced hepatic stellate cell activation under our experimental conditions.

### 3.4 Effects of fatty acids on hepatic stellate cell activation via macrophage‐derived factors

We then asked whether fatty acids could indirectly modulate stellate cell activation through macrophage‐derived mediators. To this end, stellate cells were cultured in conditioned media (CM) collected from macrophages stimulated with PA, OA, or PO.

**FIGURE 6:**
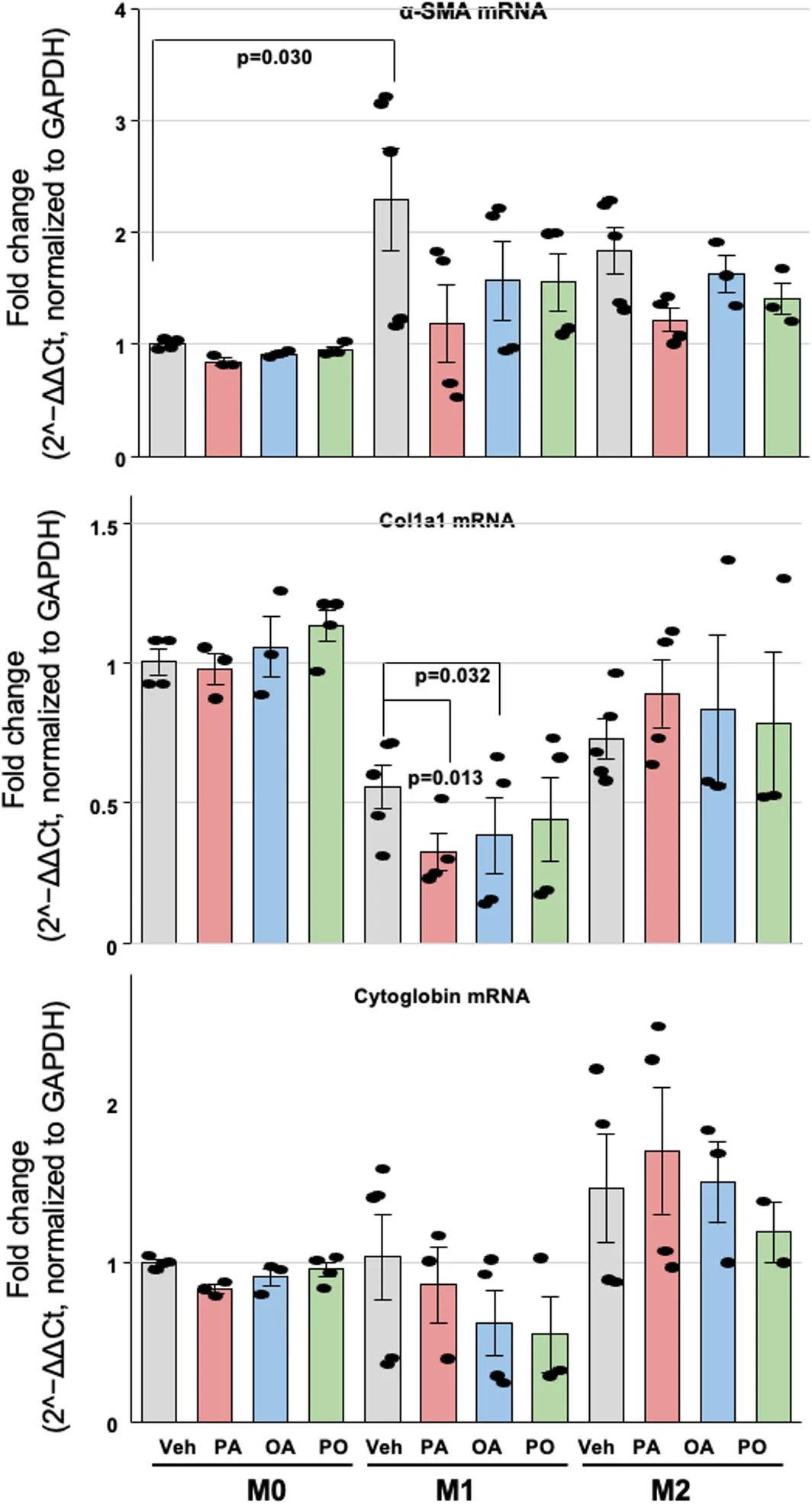
Effects of fatty acid–treated macrophage‐conditioned media on gene expression in hepatic stellate cells. Primary hepatic stellate cells (HSCs) were isolated and cultured for 3 days. Cells were maintained in high‐glucose DMEM containing 10% FBS and antibiotics. Macrophages were treated for 24 h with BSA (vehicle), BSA‐conjugated palmitic acid (PA, 0.2 mM), oleic acid (OA, 0.2 mM), or palmitoleic acid (PO, 0.2 mM) in the presence of LPS (5 ng/mL) plus IFN‐γ (1 ng/mL) or IL‐4 (10 ng/mL) plus IL‐13 (10 ng/mL), as indicated. Conditioned media were collected and mixed with fresh culture medium at a 1:1 volume ratio, and HSCs were further incubated for 3 days. mRNA expression levels of α‐SMA, Col1a1, and cytoglobin were analyzed by RT‐qPCR. Expression levels were normalized to GAPDH and calculated using the 2^−ΔΔCt^ method. Data are expressed as fold change relative to M0 vehicle control (mean ± SE, *n* = 4). Exact *p* values are shown in the figure. Statistical analysis was performed using repeated‐measures one‐way ANOVA followed by Tukey's multiple comparison test. Horizontal brackets indicate the exact groups compared.

**FIGURE 7:**
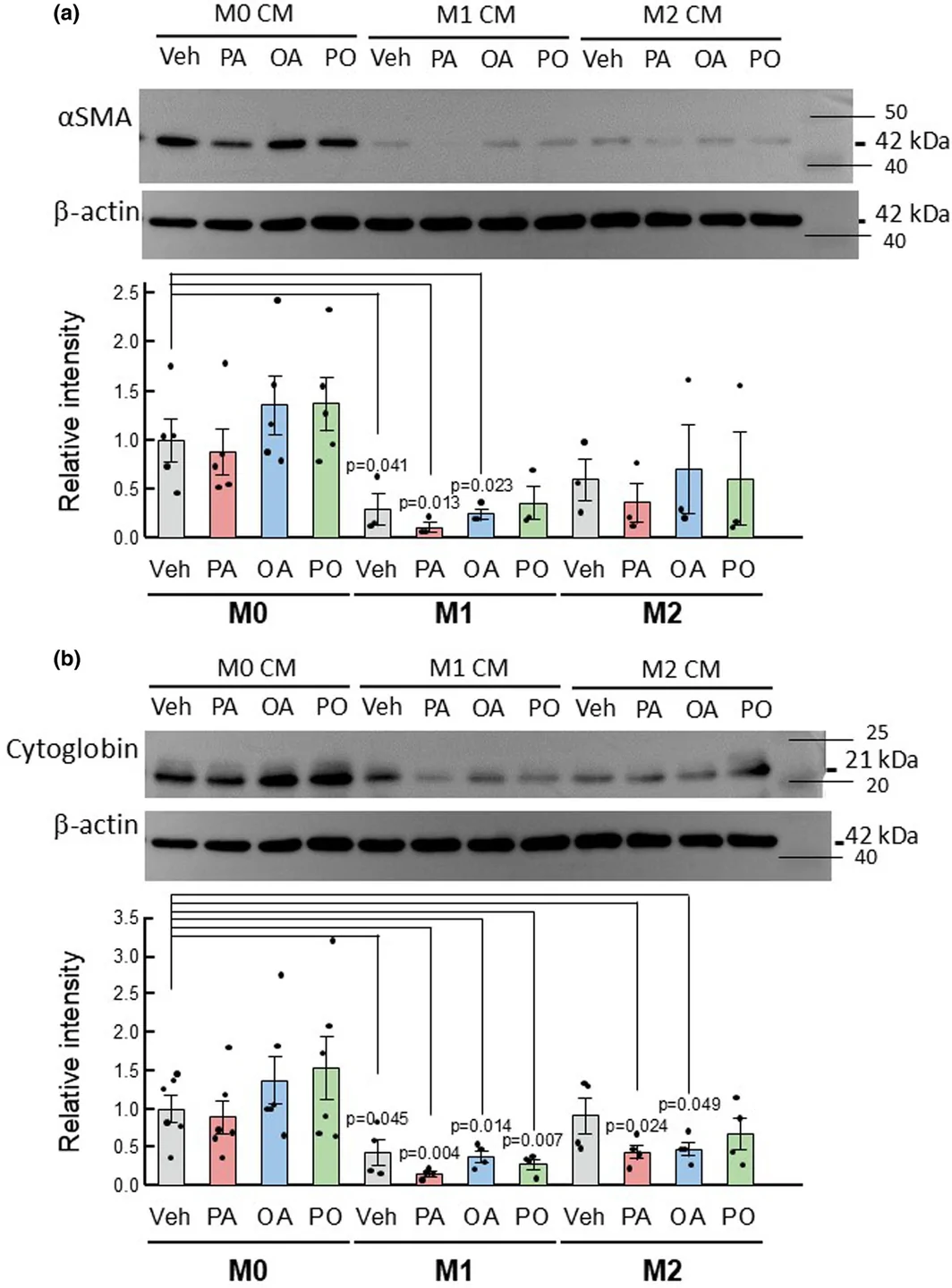
Effects of fatty acid–treated macrophage‐conditioned media on (a) α‐SMA and (b) cytoglobin protein expression in hepatic stellate cells. Primary hepatic stellate cells (HSCs) were isolated and cultured for 3 days. Cells were then maintained in high‐glucose DMEM containing 10% FBS and antibiotics. Macrophages were treated for 24 h with BSA (vehicle), BSA‐conjugated palmitic acid (PA, 0.2 mM), oleic acid (OA, 0.2 mM), or palmitoleic acid (PO, 0.2 mM), in the presence of LPS (5 ng/mL) plus IFN‐γ (1 ng/mL) or IL‐4 (10 ng/mL) plus IL‐13 (10 ng/mL), as indicated. Conditioned media were collected and mixed with fresh culture medium at a 1:1 volume ratio, and HSCs were further incubated for 3 days. Protein expression levels of α‐SMA and cytoglobin were determined by western blot analysis. Representative immunoblots are shown, and band intensities were quantified and normalized to β‐Actin as a loading control. Molecular weights of the target proteins and β‐Actin are indicated beside the blot panels. Data are presented as mean ± SE from independent experiments (*n* = 3–6). Exact *p* values are shown in the figure. Statistical analysis was performed using repeated‐measures one‐way ANOVA followed by Tukey's multiple comparison test. Representative uncropped blots are provided in File S1, and additional uncropped blots from independent experiments included in the densitometric analyses are provided in Figure S2, with sample lanes, molecular weight marker positions, migration positions, and cropped regions indicated. Horizontal brackets indicate the exact groups compared.

When stellate cells were cultured with CM from polarized macrophages, the effects on HSC markers were more dependent on macrophage polarization status than on fatty acid treatment itself. At the mRNA level, M1 macrophage CM reduced cytoglobin and α‐SMA expression, and PA‐treated M1 macrophage CM further reduced α‐SMA and Col1a1 expression compared with M1 vehicle CM (Figure 6). At the protein level, α‐SMA expression was significantly reduced in HSCs exposed to CM from M1 vehicle‐, M1 PA‐, or M1 OA‐treated macrophages compared with those exposed to M0 vehicle CM, whereas M1 PO CM showed a borderline reduction. Cytoglobin protein was also significantly reduced by CM from M1 vehicle‐, M1 PA‐, M1 OA‐, M1 PO‐, M2 PA‐, and M2 OA‐treated macrophages compared with M0 vehicle CM (Figure 7). These results indicate that macrophage polarization exerts a major influence on HSC α‐SMA and cytoglobin expression, whereas fatty acid‐specific effects are modest and marker‐dependent.

Collectively, these data suggest that fatty acid‐dependent macrophage reprogramming may secondarily modulate HSC redox‐related responses, particularly cytoglobin expression, without providing evidence for direct HSC activation by fatty acids.

## 4 DISCUSSION

In this study, we examined how distinct fatty acid species regulate macrophage polarization, oxidative stress, and macrophage–HSC crosstalk under defined in vitro conditions. PA and OA, two of the most abundant circulating fatty acids, account for approximately 31% and 27% of plasma free fatty acids, respectively (Palomer et al., 2018). Plasma free fatty acid concentrations are typically 100–200 μg/mL in healthy individuals but increase to approximately 200–300 μg/mL in patients with NASH (Sharifnia et al., 2015). In vitro studies examining fatty acid–treated macrophages commonly use concentrations of 0.4–0.5 mM (Camell & Smith, 2013; Chan et al., 2015; Lager et al., 2013). However, because 0.4 mM PA induced cytotoxicity in preliminary experiments using BMDMs, we selected 0.2 mM as a non‐cytotoxic concentration for the present study.

Among the fatty acids examined, PA exerted the strongest pro‐inflammatory effects on macrophage polarization. PA significantly increased Tnf mRNA and iNOS/TNF‐α protein expression in M1 macrophages and reduced PPARγ expression in alternatively activated macrophages, indicating partial suppression of M2‐associated features and reinforcement of inflammatory macrophage responses. Previous studies have reported that PA promotes inflammatory macrophage activation by increasing iNOS and IL‐6 expression (Chan et al., 2015). Although PA did not induce these markers in M0 macrophages in our study, it consistently suppressed several M2‐associated markers including PPARγ. Because PPARγ negatively regulates NF‐κB–mediated inflammatory signaling (Liu et al., 2021), suppression of PPARγ by PA may shift macrophage polarization toward a pro‐inflammatory phenotype.

In contrast, the unsaturated fatty acids OA and PO exerted context‐dependent effects on macrophage polarization. OA increased Nos2 and Tnf mRNA expression in M0 and M2 macrophages; however, these changes did not translate into detectable alterations at the protein level, and the magnitude of induction was minimal compared with that observed in LPS/IFN‐γ–stimulated M1 macrophages. OA therefore did not substantially drive M1 polarization under these conditions. Nevertheless, OA enhanced ROS production in M1 macrophages and reduced several M2‐associated markers, including PPARγ and Ym1, suggesting attenuation of the anti‐inflammatory M2 phenotype.

Previous reports have demonstrated anti‐inflammatory effects of OA, including reduced IL‐6 and TNF‐α production and increased IL‐10 and adiponectin levels (Santamarina et al., 2021; Scoditti et al., 2015). These effects have been linked to signaling through free fatty acid receptor 4 (FFAR4/GPR120) and adiponectin‐mediated activation of AMP‐activated protein kinase (AMPK) (Chen et al., 2018). In the present study, however, OA suppressed M2‐associated markers and enhanced ROS production in M1 macrophages, indicating that its effects are strongly context dependent. OA has also been reported to increase TLR4 expression in steatotic hepatocytes and thereby enhance inflammatory responses (Song et al., 2015). A recent study in human macrophages similarly emphasized that fatty acid composition can shape macrophage polarization through PPARγ‐related pathways (AlSaeed et al., 2026). Thus, the overall impact of OA on macrophage polarization may depend on the balance between its anti‐inflammatory and pro‐inflammatory signaling effects, as well as cell type and activation state.

PO also showed distinctive effects on macrophage responses. Although PO increased Tnf‐α mRNA expression in M0 and M2 macrophages, this did not result in detectable changes in TNF‐α protein levels, suggesting that PO does not strongly promote inflammatory macrophage polarization. In M1 macrophages, PO reduced iNOS protein expression and suppressed ROS production, while it also reduced PPARγ protein expression in M2 macrophages. PO has been reported to suppress PA‐induced inflammatory signaling via AMPK activation (Chan et al., 2015; Song et al., 2015) and to attenuate high‐fat diet–induced hepatic inflammation (Souza et al., 2014, 2017, 2020). In addition, studies in J774A.1 macrophages have shown that PO suppresses PA‐induced increases in TLR4 expression (Tsai et al., 2021). Consistent with these reports, our findings suggest that PO exerts a context‐dependent modulatory effect that can dampen inflammatory responses while also attenuating selected M2‐associated signaling pathways.

Macrophages sense inflammatory cues through pattern‐recognition receptors such as Toll‐like receptors (TLRs), which activate downstream inflammatory signaling cascades. Increased TLR4 expression has been observed in liver biopsies from patients with NASH (Palomer et al., 2018), and saturated fatty acids can activate TLR2/4 signaling pathways and promote NF‐κB–dependent inflammatory programs. PA has been linked to TLR4‐dependent signaling in macrophages (Liu et al., 2012; Sharifnia et al., 2015), consistent with our observation that PA enhances inflammatory responses. However, TLR4 signaling was not directly examined in this study and should be investigated in future work.

Another pathway by which lipid excess may regulate macrophage function involves CD36‐mediated fatty acid uptake. CD36 plays an important role in macrophage lipid metabolism and has been associated with metabolic dysfunction and AMPK‐related signaling pathways (Carta et al., 2017). Although we did not examine CD36 expression or AMPK activation in this study, these mechanisms may contribute to fatty acid–dependent macrophage reprogramming.

Because oxidative stress plays a key role in liver fibrogenesis, we also examined the effects of fatty acids on ROS production in polarized macrophages (Parola & Robino, 2001). Macrophage‐derived ROS contribute to microbial defense and inflammatory signaling, and M2 macrophages can also produce ROS under certain conditions (Lewis et al., 2019). Using the chemiluminescent probe L‐012, we observed that ROS production was markedly higher in M1 macrophages than in M0 or M2 macrophages. Fatty acid treatment had minimal effects on ROS production in M0 and M2 macrophages, whereas PA and OA significantly increased ROS production in M1 macrophages.

The majority of ROS detected in our experiments was superoxide (O_2_·^−^), which is commonly generated by mitochondria and NADPH oxidase complexes (Lewis et al., 2019). Previous studies have shown that PA stimulates ROS production and contributes to lipotoxicity through oxidative stress mechanisms (Fauser et al., 2013; Rosca et al., 2012). High levels of circulating free fatty acids can also activate NADPH oxidase (NOX) and enhance ROS generation (Chen et al., 2018). Consistent with these findings, our data suggest that PA‐induced oxidative stress in inflammatory macrophages may involve NOX‐dependent ROS production.

To determine how fatty acids influence macrophage–HSC communication, we next examined their effects on hepatic stellate cells. Direct treatment of primary stellate cells with PA, OA, or PO did not significantly alter α‐SMA expression or other fibrosis‐related markers, indicating that fatty acids alone did not directly activate stellate cells under these experimental conditions. Instead, fatty acids influenced stellate cell marker expression only indirectly and modestly through macrophage‐derived signals.

Conditioned media from polarized macrophages altered α‐SMA and cytoglobin expression in stellate cells, but the revised protein quantification indicates that the effect was driven mainly by macrophage polarization status rather than by fatty acid treatment. Cytoglobin is an antioxidant protein that plays an important protective role in stellate cells by scavenging reactive oxygen species and maintaining redox homeostasis (Okina et al., 2020; Thi Thanh Hai et al., 2018). Reduced cytoglobin expression may therefore weaken antioxidant defenses (Okina et al., 2020) and increase susceptibility to oxidative stress. Our results suggest that fatty acid–induced macrophage reprogramming may influence the cellular microenvironment associated with fibrosis‐related processes by modifying macrophage‐derived signals that regulate stellate cell antioxidant capacity; however, the present data do not support a simple conclusion that fatty acid‐treated macrophages directly enhance HSC activation.

Indeed, stellate cells cultured with conditioned media from M1 or M2 macrophages exhibited reduced expression of both α‐SMA and cytoglobin compared with cells cultured with conditioned media from M0 macrophages. These findings indicate that macrophage‐derived mediators can modulate stellate cell phenotype independently of direct fatty acid exposure. In inflammatory macrophage‐conditioned media, cytokines such as TNF‐α and macrophage‐derived ROS may contribute to suppression of cytoglobin expression and alteration of stellate cell redox homeostasis. In addition, LPS has been reported to suppress α‐SMA expression in stellate cells (Ho et al., 2021).

Although M2 macrophages are often associated with tissue repair and fibrosis through production of profibrotic mediators such as TGF‐β, conditioned media from IL‐4/IL‐13–polarized macrophages did not significantly enhance α‐SMA expression in our primary stellate cell system. Previous studies have shown that IL‐4 and IL‐13 can stimulate collagen production and stellate cell activation in vitro (Sugimoto et al., 2005; Sui et al., 2018); however, robust activation of stellate cells typically requires sustained exposure to bioactive TGF‐β together with additional inflammatory or oxidative signals. The concentration of active TGF‐β present in our conditioned media may therefore have been insufficient to induce overt stellate cell activation.

Although some fatty acid‐treated CM groups showed modest marker‐specific changes, these effects were not uniform across α‐SMA, Col1a1, and cytoglobin at the mRNA and protein levels. Therefore, we interpret the conditioned medium experiments as evidence that fatty acid‐dependent macrophage reprogramming can secondarily modify HSC redox‐related responses, rather than as definitive evidence that fatty acids regulate HSC activation through macrophage‐derived factors.

Several limitations of this study should be considered. First, although saturated fatty acids are known to activate inflammatory signaling through receptors such as TLR4, we did not directly assess TLR4‐dependent signaling pathways in this study. Future work should examine the involvement of TLR4, NF‐κB, and related pathways in fatty acid–induced macrophage reprogramming. Second, ROS production was evaluated using L‐012 chemiluminescence. Although SOD‐sensitive L‐012 signals are useful for detecting superoxide generation, additional approaches such as DCFH‐DA fluorescence, mitochondrial ROS probes, or oxidative stress marker analysis would strengthen the evaluation of macrophage redox responses. Third, the present study used in vitro macrophage polarization and stellate cell culture systems. Although this reductionist approach allowed us to dissect lipid species–dependent macrophage–HSC communication, the in vivo relevance of these findings should be validated in experimental models of MASH. Finally, HSC activation was evaluated mainly by marker expression, and functional assays of proliferation, migration, and contractility were not performed. Further studies are needed to determine how physiological lipid exposure conditions influence macrophage‐stromal interactions and HSC function in vivo.

In conclusion, our findings demonstrate that fatty acid composition regulates macrophage polarization and oxidative activity in a lipid species–dependent manner. In particular, PA suppresses selected PPARγ‐associated M2 features and promotes inflammatory macrophage reprogramming, whereas OA and PO exert context‐dependent effects on ROS production and polarization markers. This macrophage reprogramming alters macrophage‐derived paracrine signals and modulates stellate cell antioxidant capacity without directly activating stellate cells. These results highlight lipid species composition as an important determinant of immune–stromal interactions in the liver and suggest a potential mechanism linking metabolic stress to fibrogenic signaling in metabolic liver disease.

## 5 IMPACT AND IMPLICATIONS

Metabolic dysfunction–associated steatohepatitis (MASH) is characterized by excessive lipid accumulation, chronic inflammation, and progressive fibrosis, yet the mechanisms linking lipid species to immune–stromal interactions remain incompletely understood.

This study demonstrates that distinct fatty acid species differentially reprogram macrophage polarization and redox activity. In particular, saturated fatty acids suppress selected PPARγ‐associated M2 features and promote inflammatory macrophage phenotypes, leading to altered macrophage‐derived paracrine signaling that modulates cytoglobin expression in hepatic stellate cells.

These findings suggest that lipid species composition is a determinant of macrophage‐driven regulation of stellate cell redox‐related responses and may influence the fibrogenic microenvironment in metabolic liver disease.

Our results highlight macrophage metabolic reprogramming as a potential functional link between lipid overload and stromal responses in MASH and suggest that modulation of lipid‐driven macrophage signaling pathways may represent a potential strategy to regulate inflammation‐ and fibrosis‐related microenvironmental changes.

## 6 LIMITATIONS

Several limitations of this study should be acknowledged. First, although previous studies suggest that saturated fatty acids activate macrophage inflammatory signaling through pattern‐recognition receptors such as Toll‐like receptor 4 (TLR4), the involvement of TLR4‐dependent pathways was not directly examined in this study. Future studies evaluating TLR4 expression, downstream NF‐κB activation, and related signaling pathways will be necessary to clarify the molecular mechanisms underlying fatty acid–induced macrophage reprogramming.

Second, ROS production was assessed primarily using L‐012 chemiluminescence. Although the SOD‐sensitive signal supported superoxide detection, additional assays such as DCFH‐DA fluorescence, mitochondrial ROS probes, or oxidative stress marker measurements would be useful to further validate the redox changes observed in BMDMs.

Third, the present study was performed using in vitro macrophage polarization and primary stellate cell culture systems. While this reductionist approach allowed us to dissect lipid species–dependent macrophage–HSC communication under controlled conditions, the in vivo relevance of these findings remains to be established. Future studies using dietary or genetic models of MASH will be required to determine whether lipid species–dependent macrophage reprogramming similarly influences macrophage–stellate cell interactions during fibrosis progression in vivo.

Fourth, HSC activation was evaluated mainly by marker expression. Functional assays assessing stellate cell proliferation, migration, and contractility were not performed. Therefore, the present findings should be interpreted as changes in HSC marker expression and redox‐related responses rather than definitive evidence of altered HSC activation. The macrophage‐derived mediator(s) responsible for changes in cytoglobin expression in stellate cells also remain to be identified.

Fifth, the fatty acid concentrations used in this study were selected based on commonly used experimental conditions and preliminary cytotoxicity testing. Although circulating free fatty acid levels are elevated in metabolic syndrome and NASH, local fatty acid concentrations within the hepatic microenvironment may differ from systemic levels. Therefore, further studies will be required to determine how physiological lipid exposure conditions regulate macrophage–stromal interactions in vivo.

Finally, several experiments were performed with relatively small numbers of independent biological replicates (*n* = 3–6). Although directional changes were assessed across independent experiments, this sample size limits statistical precision. Therefore, the present findings should be interpreted as exploratory mechanistic observations requiring confirmation in larger independent experiments and relevant in vivo models.

## 7 CONCLUSION

In summary, fatty acid species differentially modulate macrophage polarization and redox activity under inflammatory conditions. These changes in macrophage phenotype alter macrophage‐derived signals that influence stellate cell redox‐related gene expression. Our findings provide insight into how lipid composition may regulate immune–stromal interactions in the liver.

## AUTHOR CONTRIBUTIONS

**Koichi Nakanishi:** Investigation; methodology. **Hiroji Shinkawa:** Data curation; validation. **Shigekazu Takemura:** Conceptualization; data curation; formal analysis; funding acquisition. **Kanako Nakagawa:** Investigation. **Yukiko Minamiyama:** Conceptualization; data curation; formal analysis; funding acquisition; methodology; project administration; supervision; visualization. **Takeaki Ishizawa:** Resources; supervision.

## FUNDING INFORMATION

This work was supported by institutional research funding and related collaborative research programs and by Japan Society for the Promotion of Science (JSPS KAKENHI) Grant Numbers 21K08690 (2021–2023) for Takemura and Minamiyama.

## CONFLICT OF INTEREST STATEMENT

The authors declare that they have no competing interests related to this work.

## ETHICS STATEMENT

All animal procedures were approved by the Animal Care and Use Committee of Osaka Metropolitan University (Approval No. 21002) and conducted in accordance with ARRIVE guidelines.

## Supporting information


**Table S1.** Antibodies used for western blot analysis.
**Table S2.** Primer sequences and TaqMan assays used for RT‐qPCR analysis (rat).

**File S1.** Original images of membrane sections used for western blot analysis.

**Figure S2.** Original images of membrane sections used for western blot analysis from all independent experiments included in the densitometric analyses are provided. During the original experiments, the membranes were cut horizontally according to the expected molecular‐weight ranges of the target proteins before antibody incubation. Therefore, intact images of the full uncut membranes are not available. The images presented here are the complete original images available for each membrane section used for detection and densitometric analysis. For each blot, the corresponding main‐figure panel, target protein, visible molecular‐weight marker positions, sample order, and experimental date are indicated. Different pre‐stained molecular‐weight markers were used among the independent experiments because the experiments were performed at different times and, in some cases, at different experimental sites. Single‐color and three‐color pre‐stained markers were used depending on the experiment. Therefore, the molecular‐weight reference bands visible in the original membrane images differ among blots, and only marker positions that can be clearly identified in each original image are labeled. Blots included in the quantitative analysis are identified accordingly. β‐Actin was used as the loading control. For most experiments, β‐actin was detected by reprobing the same membrane after target‐protein detection. Where β‐actin was obtained from a separately run gel and membrane using aliquots of the same protein samples, this is explicitly indicated for the corresponding blot. The corresponding β‐actin image is shown for each target‐protein blot or experimental set.

## Data Availability

The datasets generated and/or analyzed during the current study are available from the corresponding author upon reasonable request.

## References

[phy271082-bib-0001] AlSaeed, H. , Almusallam, H. , Menezes, S. , Almelaifi, H. , Alonaizi, H. , & Almejaimi, M. (2026). Fatty acid composition, at equivalent lipid exposure, dictates human macrophage polarization via PPARgamma signaling. Cells, 15, 308. 41677671 10.3390/cells15030308 PMC12897183

[phy271082-bib-0002] Camell, C. , & Smith, C. W. (2013). Dietary oleic acid increases m2 macrophages in the mesenteric adipose tissue. PLoS One, 8, e75147. 24098682 10.1371/journal.pone.0075147 PMC3787090

[phy271082-bib-0003] Carta, G. , Murru, E. , Banni, S. , & Manca, C. (2017). Palmitic acid: Physiological role, metabolism and nutritional implications. Frontiers in Physiology, 8, 902. 29167646 10.3389/fphys.2017.00902 PMC5682332

[phy271082-bib-0004] Chan, K. L. , Pillon, N. J. , Sivaloganathan, D. M. , Costford, S. R. , Liu, Z. , Theret, M. , Chazaud, B. , & Klip, A. (2015). Palmitoleate reverses high fat‐induced Proinflammatory macrophage polarization via AMP‐activated protein kinase (AMPK). Journal of Biological Chemistry, 290, 16979–16988. 25987561 10.1074/jbc.M115.646992 PMC4505442

[phy271082-bib-0005] Chen, X. , Li, L. , Liu, X. , Luo, R. , Liao, G. , Li, L. , Liu, J. , Cheng, J. , Lu, Y. , & Chen, Y. (2018). Oleic acid protects saturated fatty acid mediated lipotoxicity in hepatocytes and rat of non‐alcoholic steatohepatitis. Life Sciences, 203, 291–304. 29709653 10.1016/j.lfs.2018.04.022

[phy271082-bib-0006] Devaraj, S. , & Jialal, I. (2011). C‐reactive protein polarizes human macrophages to an M1 phenotype and inhibits transformation to the M2 phenotype. Arteriosclerosis, Thrombosis, and Vascular Biology, 31, 1397–1402. 21415385 10.1161/ATVBAHA.111.225508 PMC3099471

[phy271082-bib-0007] Eslam, M. , Newsome, P. N. , Sarin, S. K. , Anstee, Q. M. , Targher, G. , Romero‐Gomez, M. , Zelber‐Sagi, S. , Wai‐Sun Wong, V. , Dufour, J. F. , Schattenberg, J. M. , Kawaguchi, T. , Arrese, M. , Valenti, L. , Shiha, G. , Tiribelli, C. , Yki‐Järvinen, H. , Fan, J. G. , Grønbæk, H. , Yilmaz, Y. , … George, J. (2020). A new definition for metabolic dysfunction‐associated fatty liver disease: An international expert consensus statement. Journal of Hepatology, 73, 202–209. 32278004 10.1016/j.jhep.2020.03.039

[phy271082-bib-0008] Fauser, J. K. , Matthews, G. M. , Cummins, A. G. , & Howarth, G. S. (2013). Induction of apoptosis by the medium‐chain length fatty acid lauric acid in colon cancer cells due to induction of oxidative stress. Chemotherapy, 59, 214–224. 24356281 10.1159/000356067

[phy271082-bib-0009] Friedman, S. L. (2008). Hepatic stellate cells: Protean, multifunctional, and enigmatic cells of the liver. Physiological Reviews, 88, 125–172. 18195085 10.1152/physrev.00013.2007 PMC2888531

[phy271082-bib-0010] Ho, C. H. , Huang, J. H. , Sun, M. S. , Tzeng, I. S. , Hsu, Y. C. , & Kuo, C. Y. (2021). Wild bitter melon extract regulates LPS‐induced hepatic stellate cell activation, inflammation, endoplasmic reticulum stress, and Ferroptosis. Evidence‐Based Complementary and Alternative Medicine, 2021, 6671129. 34239589 10.1155/2021/6671129 PMC8241502

[phy271082-bib-0011] Hotamisligil, G. S. (2006). Inflammation and metabolic disorders. Nature, 444, 860–867. 17167474 10.1038/nature05485

[phy271082-bib-0012] Kodai, S. , Takemura, S. , Minamiyama, Y. , Hai, S. , Yamamoto, S. , Kubo, S. , Yoshida, Y. , Niki, E. , Okada, S. , Hirohashi, K. , & Suehiro, S. (2007). S‐allyl cysteine prevents CCl(4)‐induced acute liver injury in rats. Free Radical Research, 41, 489–497. 17454131 10.1080/10715760601118361

[phy271082-bib-0013] Lager, S. , Gaccioli, F. , Ramirez, V. I. , Jones, H. N. , Jansson, T. , & Powell, T. L. (2013). Oleic acid stimulates system a amino acid transport in primary human trophoblast cells mediated by toll‐like receptor 4. Journal of Lipid Research, 54, 725–733. 23275648 10.1194/jlr.M033050 PMC3617946

[phy271082-bib-0014] Lee, S. , Huen, S. , Nishio, H. , Nishio, S. , Lee, H. K. , Choi, B. S. , Ruhrberg, C. , & Cantley, L. G. (2011). Distinct macrophage phenotypes contribute to kidney injury and repair. Journal of the American Society of Nephrology, 22, 317–326. 21289217 10.1681/ASN.2009060615 PMC3029904

[phy271082-bib-0015] Lewis, C. V. , Vinh, A. , Diep, H. , Samuel, C. S. , Drummond, G. R. , & Kemp‐Harper, B. K. (2019). Distinct redox Signalling following macrophage activation influences Profibrotic activity. Journal of Immunology Research, 2019, 1278301. 31815149 10.1155/2019/1278301 PMC6877990

[phy271082-bib-0016] Liu, H. , Wang, M. , Jin, Z. , Sun, D. , Zhu, T. , & Liu, X. (2021). FNDC5 induces M2 macrophage polarization and promotes hepatocellular carcinoma cell growth by affecting the PPARgamma/NF‐kappaB/NLRP3 pathway. Biochemical and Biophysical Research Communications, 582, 77–85. 34695754 10.1016/j.bbrc.2021.10.041

[phy271082-bib-0017] Liu, S. P. , Li, X. Y. , Li, Z. , He, L. N. , Xiao, Y. , & Yan, K. (2012). Octanoylated ghrelin inhibits the activation of the palmitic acid‐induced TLR4/NF‐kappaB signaling pathway in THP‐1 macrophages. ISRN Endocrinology, 2012, 237613. 23251812 10.5402/2012/237613 PMC3513732

[phy271082-bib-0018] Okina, Y. , Sato‐Matsubara, M. , Matsubara, T. , Daikoku, A. , Longato, L. , & Rombouts, K. (2020). TGF‐beta1‐driven reduction of cytoglobin leads to oxidative DNA damage in stellate cells during non‐alcoholic steatohepatitis. Journal of Hepatology, 73, 882–895. 32330605 10.1016/j.jhep.2020.03.051

[phy271082-bib-0019] Palomer, X. , Pizarro‐Delgado, J. , Barroso, E. , & Vazquez‐Carrera, M. (2018). Palmitic and oleic acid: The Yin and Yang of fatty acids in type 2 diabetes mellitus. Trends in Endocrinology and Metabolism, 29, 178–190. 29290500 10.1016/j.tem.2017.11.009

[phy271082-bib-0020] Parola, M. , & Robino, G. (2001). Oxidative stress‐related molecules and liver fibrosis. Journal of Hepatology, 35, 297–306. 11580156 10.1016/s0168-8278(01)00142-8

[phy271082-bib-0021] Rosca, M. G. , Vazquez, E. J. , Chen, Q. , Kerner, J. , Kern, T. S. , & Hoppel, C. L. (2012). Oxidation of fatty acids is the source of increased mitochondrial reactive oxygen species production in kidney cortical tubules in early diabetes. Diabetes, 61, 2074–2083. 22586586 10.2337/db11-1437 PMC3402323

[phy271082-bib-0022] Santamarina, A. B. , Pisani, L. P. , Baker, E. J. , Marat, A. D. , Valenzuela, C. A. , & Miles, E. A. (2021). Anti‐inflammatory effects of oleic acid and the anthocyanin keracyanin alone and in combination: Effects on monocyte and macrophage responses and the NF‐kappaB pathway. Food & Function, 12, 7909–7922. 34250536 10.1039/d1fo01304a

[phy271082-bib-0023] Scoditti, E. , Massaro, M. , Carluccio, M. A. , Pellegrino, M. , Wabitsch, M. , Calabriso, N. , Storelli, C. , & de Caterina, R. (2015). Additive regulation of adiponectin expression by the mediterranean diet olive oil components oleic acid and hydroxytyrosol in human adipocytes. PLoS One, 10, e0128218. 26030149 10.1371/journal.pone.0128218 PMC4452359

[phy271082-bib-0024] Sharifnia, T. , Antoun, J. , Verriere, T. G. , Suarez, G. , Wattacheril, J. , Wilson, K. T. , Verriere, T. G. C. , Peek, R. M. , Abumrad, N. N. , & Flynn, C. R. (2015). Hepatic TLR4 signaling in obese NAFLD. American Journal of Physiology. Gastrointestinal and Liver Physiology, 309, G270–G278. 26113297 10.1152/ajpgi.00304.2014 PMC4537925

[phy271082-bib-0025] Shi, H. , Kokoeva, M. V. , Inouye, K. , Tzameli, I. , Yin, H. , & Flier, J. S. (2006). TLR4 links innate immunity and fatty acid‐induced insulin resistance. The Journal of Clinical Investigation, 116, 3015–3025. 17053832 10.1172/JCI28898 PMC1616196

[phy271082-bib-0026] Song, C. Y. , Zeng, X. , Wang, Y. , Shi, J. , Qian, H. , Zhang, Y. , Fang, J. Q. , Sheng, X. , Zheng, J. M. , & Chen, Y. X. (2015). Sophocarpine attenuates toll‐like receptor 4 in steatotic hepatocytes to suppress pro‐inflammatory cytokines synthesis. Journal of Gastroenterology and Hepatology, 30, 405–412. 25089018 10.1111/jgh.12691

[phy271082-bib-0027] Souza, C. O. , Teixeira, A. A. , Biondo, L. A. , Silveira, L. S. , Calder, P. C. , & Rosa Neto, J. C. (2017). Palmitoleic acid reduces the inflammation in LPS‐stimulated macrophages by inhibition of NFkappaB, independently of PPARs. Clinical and Experimental Pharmacology & Physiology, 44, 566–575. 28135761 10.1111/1440-1681.12736

[phy271082-bib-0028] Souza, C. O. , Teixeira, A. A. , Lima, E. A. , Batatinha, H. A. , Gomes, L. M. , & Carvalho‐Silva, M. (2014). Palmitoleic acid (n‐7) attenuates the immunometabolic disturbances caused by a high‐fat diet independently of PPARalpha. Mediators of Inflammation, 2014, 582197. 25147439 10.1155/2014/582197 PMC4131426

[phy271082-bib-0029] Souza, C. O. , Teixeira, A. A. S. , Biondo, L. A. , Silveira, L. S. , de Souza Breda, C. N. , & Braga, T. T. (2020). Palmitoleic acid reduces high fat diet‐induced liver inflammation by promoting PPAR‐gamma‐independent M2a polarization of myeloid cells. Biochimica et Biophysica Acta, Molecular and Cell Biology of Lipids, 1865, 158776. 32738301 10.1016/j.bbalip.2020.158776 PMC7487782

[phy271082-bib-0030] Sugimoto, R. , Enjoji, M. , Nakamuta, M. , Ohta, S. , Kohjima, M. , Fukushima, M. , Kuniyoshi, M. , Arimura, E. , Morizono, S. , Kotoh, K. , & Nawata, H. (2005). Effect of IL‐4 and IL‐13 on collagen production in cultured LI90 human hepatic stellate cells. Liver International, 25, 420–428. 15780068 10.1111/j.1478-3231.2005.01087.x

[phy271082-bib-0031] Sui, G. , Cheng, G. , Yuan, J. , Hou, X. , Kong, X. , & Niu, H. (2018). Interleukin (IL)‐13, prostaglandin E2 (PGE2), and prostacyclin 2 (PGI2) activate hepatic stellate cells via protein kinase C (PKC) pathway in hepatic fibrosis. Medical Science Monitor, 24, 2134–2141. 29633755 10.12659/MSM.906442 PMC5909417

[phy271082-bib-0032] Sun, Y. Y. , Li, X. F. , Meng, X. M. , Huang, C. , Zhang, L. , & Li, J. (2017). Macrophage phenotype in liver injury and repair. Scandinavian Journal of Immunology, 85, 166–174. 27491503 10.1111/sji.12468

[phy271082-bib-0033] Takemura, S. , Azuma, H. , Osada‐Oka, M. , Kubo, S. , Shibata, T. , & Minamiyama, Y. (2018). S‐allyl‐glutathione improves experimental liver fibrosis by regulating Kupffer cell activation in rats. American Journal of Physiology. Gastrointestinal and Liver Physiology, 314, G150–G163. 28971836 10.1152/ajpgi.00023.2017

[phy271082-bib-0034] Thi Thanh Hai, N. , Thuy, L. T. T. , Shiota, A. , Kadono, C. , Daikoku, A. , Hoang, D. V. , Dat, N. Q. , Sato‐Matsubara, M. , Yoshizato, K. , & Kawada, N. (2018). Selective overexpression of cytoglobin in stellate cells attenuates thioacetamide‐induced liver fibrosis in mice. Scientific Reports, 8, 17860. 30552362 10.1038/s41598-018-36215-4 PMC6294752

[phy271082-bib-0035] Tilg, H. , & Moschen, A. R. (2010). Evolution of inflammation in nonalcoholic fatty liver disease: The multiple parallel hits hypothesis. Hepatology, 52, 1836–1846. 21038418 10.1002/hep.24001

[phy271082-bib-0036] Tsai, Y. W. , Lu, C. H. , Chang, R. C. , Hsu, Y. P. , Ho, L. T. , & Shih, K. C. (2021). Palmitoleic acid ameliorates palmitic acid‐induced proinflammation in J774A.1 macrophages via TLR4‐dependent and TNF‐alpha‐independent signallings. Prostaglandins, Leukotrienes, and Essential Fatty Acids, 169, 102270. 33930845 10.1016/j.plefa.2021.102270

[phy271082-bib-0037] Xuan, W. , Qu, Q. , Zheng, B. , Xiong, S. , & Fan, G. H. (2015). The chemotaxis of M1 and M2 macrophages is regulated by different chemokines. Journal of Leukocyte Biology, 97, 61–69. 25359998 10.1189/jlb.1A0314-170R

