# Supplementary material for: Fatty acid species differentially regulate macrophage polarization and oxidative stress with secondary effects on macrophage–HSC crosstalk
Source: Physiol Rep. 2026 Sep 10;14(17):e71082. doi: 10.14814/phy2.71082 (PMC13562811; doi:10.14814/phy2.71082)
Supplement: Supplementary file 1 [file PHY2-14-e71082-s001.pdf]

Supplementary Table S1. Antibodies used for Western blot analysis

Target protein	Antibody (host/clone)	Supplier (City, Country)	Catalog No.	Lot No.	Dilution	Secondary antibody
iNOS (NOS2)	Mouse monoclonal anti-iNOS	BD Transduction Laboratories (Franklin Lakes, NJ, USA)	610328	4136577	1:5000	HRP-conjugated goat anti-mouse IgG (Dako, Glostrup, Denmark)
PPARγ	Rabbit monoclonal anti-PPARγ (C26H12)	Cell Signaling Technology (Danvers, MA, USA)	2435	4	1:2000	HRP-conjugated goat anti-rabbit IgG (Dako, Glostrup, Denmark)
CD206 (MR)	Rabbit polyclonal anti-mannose receptor	Abcam (Cambridge, UK)	ab64693	GR3234777-1	1:5000	HRP-conjugated goat anti-rabbit IgG (Dako, Glostrup, Denmark)
α-SMA	Mouse monoclonal anti-α-smooth muscle actin (Clone 1A4)	Dako (Glostrup, Denmark)	M0851	20019176	1:5000	HRP-conjugated goat anti-mouse IgG (Dako, Glostrup, Denmark)
Cytoglobin	Rabbit polyclonal anti-cytoglobin (kindly provided by Prof. Norifumi Kawada, Osaka Metropolitan University, Japan)	N/A	N/A	N/A	1:2000	HRP-conjugated goat anti-rabbit IgG (Dako, Glostrup, Denmark)
β-actin	Mouse monoclonal anti-β-actin	Sigma-Aldrich (St. Louis, MO, USA)	A5441	079M4799V	1:5000	HRP-conjugated goat anti-mouse IgG (Dako, Glostrup, Denmark)

Supplementary Table S2. Primer sequences and TaqMan assays used for RT-qPCR analysis (Rat)

A. Custom-designed primers and probes

Gene (Symbol)	Direction	Sequence (5′–3′)
GAPDH	Forward	AAGATGGTGAAGGTCGGTGTG
	Reverse	GAAGGCAGCCCTGGTAACC
	Probe	6-FAM-CGGATTTGGCCGTATCGGACGC-TAMRA
TGF-β1 (Tgfb1)	Forward	TGCTTCCGCATCACCGT
	Reverse	TAGTAGACGATGGGCAGTGGC
	Probe	6-FAM-CTGCGTGCCGCAGGCTTTGG-TAMRA
β-actin (Actb)	Forward	CCTCTGAACCCTAAGGCCAA
	Reverse	AGCCTGGATGGCTACGTACA
	Probe	6-FAM-TGACCCAGATCATGTTTGAGACCTTCAAC-TAMRA
Collagen type I alpha 1 (Col1a1)	Forward	CCCACCGGCCCTACTG
	Reverse	GACCAGCTTCACCCTTAGCA
	Probe	6-FAM-CCTCCTGGCTTCCCTG-TAMRA
α-SMA (Acta2)	Forward	GAGGAGCATCCGACCTTGC
	Reverse	TTTCTCCCGGTTGGCCTTA
	Probe	6-FAM-AACGGAGGCGCCGCTGAACC-TAMRA

B. Commercial TaqMan Gene Expression Assays (Applied Biosystems, Foster City, CA, USA)

Gene (Symbol)	Assay ID	Reporter
NOS2	Rn00561646_m1	FAM-MGB
TNF-α (Tnf)	Rn99999017_m1	FAM-MGB
PPARγ (Pparg)	Rn00440945_m1	FAM-MGB
Ym1 (Chi3l3)	Rn01490246_m1	FAM-MGB
Cytoglobin (Cygb)	Rn00590627_m1	FAM-MGB
