# Supplementary material for: Fatty acid species differentially regulate macrophage polarization and oxidative stress with secondary effects on macrophage–HSC crosstalk
Source: Physiol Rep. 2026 Sep 10;14(17):e71082. doi: 10.14814/phy2.71082 (PMC13562811; doi:10.14814/phy2.71082)

Supplementary Figure S2.

Original images of membrane sections used for Western blot analysis from all independent experiments included in the densitometric analyses are provided. During the original experiments, the membranes were cut horizontally according to the expected molecular-weight ranges of the target proteins before antibody incubation. Therefore, intact images of the full uncut membranes are not available. The images presented here are the complete original images available for each membrane section used for detection and densitometric analysis. For each blot, the corresponding main-figure panel, target protein, visible molecular-weight marker positions, sample order, and experimental date are indicated. Different pre-stained molecular-weight markers were used among the independent experiments because the experiments were performed at different times and, in some cases, at different experimental sites. Single-color and three-color pre-stained markers were used depending on the experiment. Therefore, the molecular-weight reference bands visible in the original membrane images differ among blots, and only marker positions that can be clearly identified in each original image are labeled. Blots included in the quantitative analysis are identified accordingly. β-Actin was used as the loading control. For most experiments, β-actin was detected by reprobing the same membrane after target-protein detection. Where β-actin was obtained from a separately run gel and membrane using aliquots of the same protein samples, this is explicitly indicated for the corresponding blot. The corresponding β-actin image is shown for each target-protein blot or experimental set.

Fig. 1c

Blot ID: #1605
Target: iNOS
Expected molecular weight: approximately 130 kDa

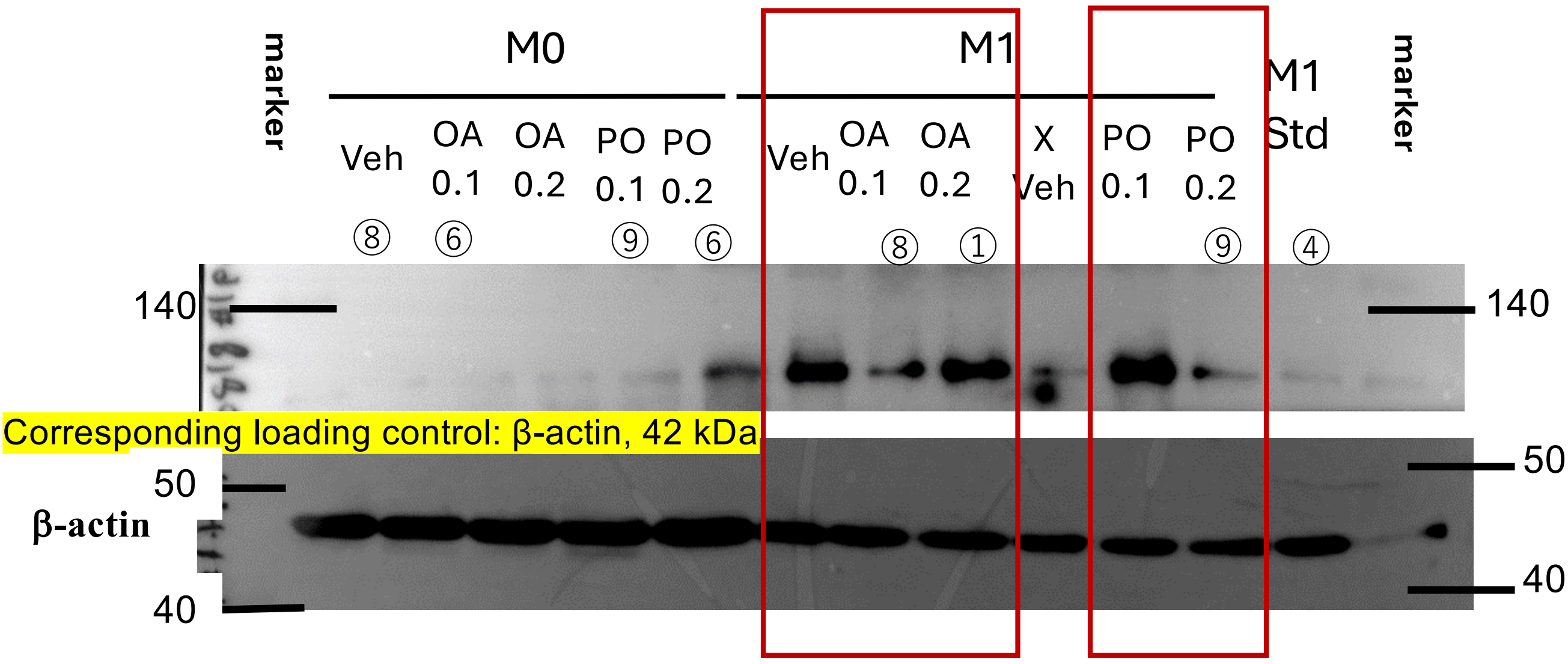

Blot ID: #1308
Target: iNOS
Expected molecular weight: approximately 130 kDa

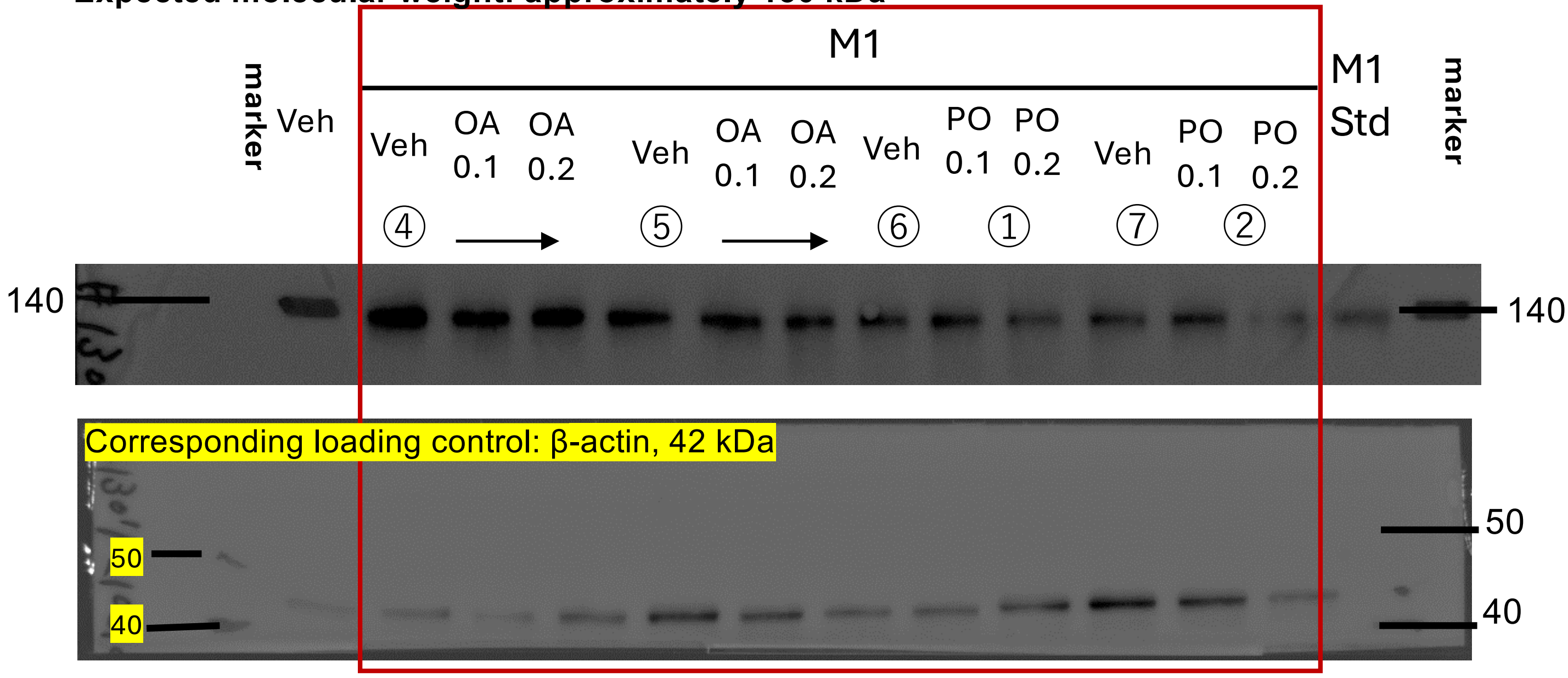

Blot ID: #1314
Target: iNOS
Expected molecular weight: approximately 130 kDa

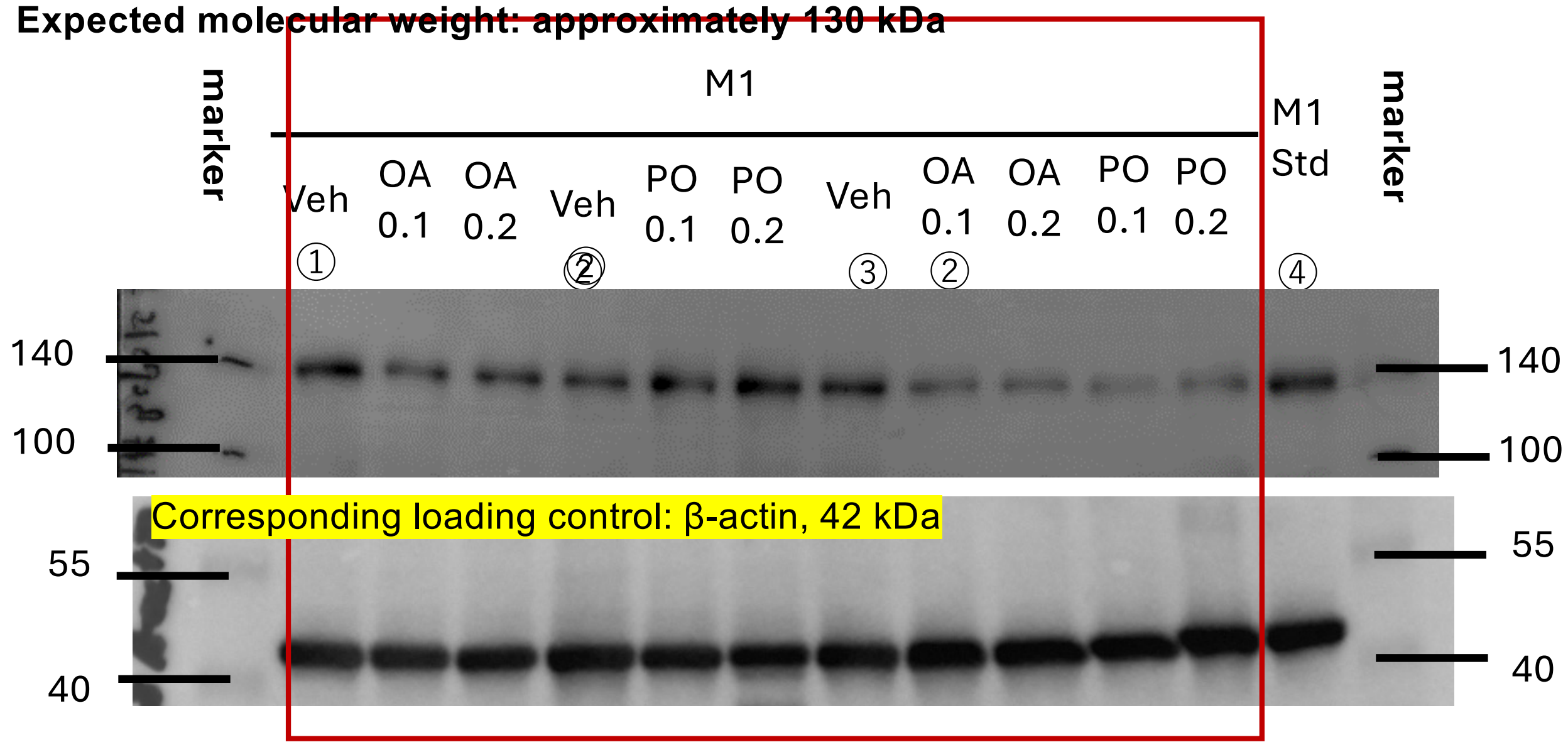

Blot ID: #1205
Target: iNOS
Expected molecular weight: approximately 130 kDa

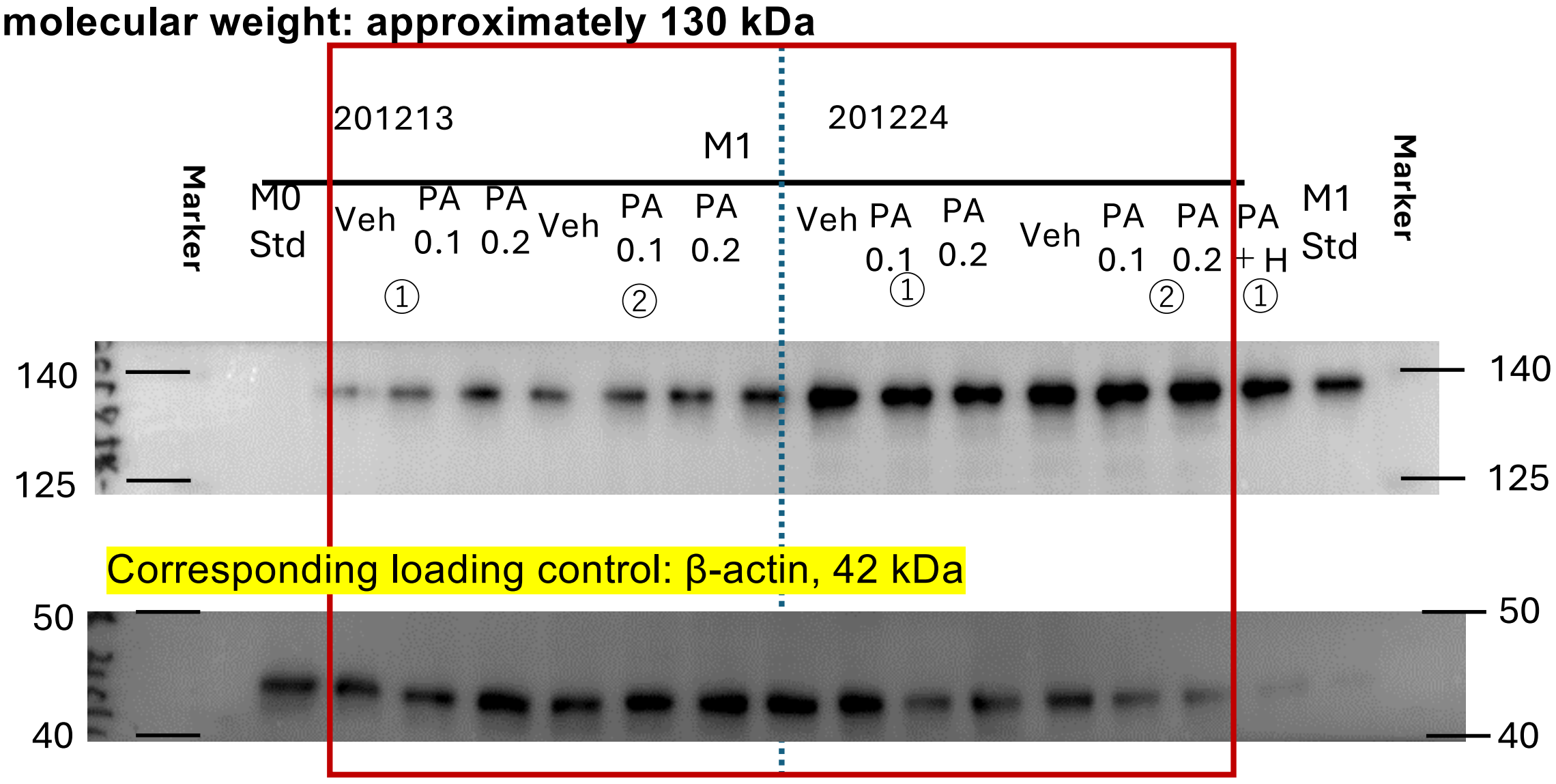

Fig. 1d TNF-α

The representative TNF-α blot is shown in Supplementary Fig. S1. The images below are the remaining independent blots included in the quantitative analysis for Fig. 1d.

Blot ID: #1606
Target: TNF-α
Expected molecular weight: approximately 17 kDa

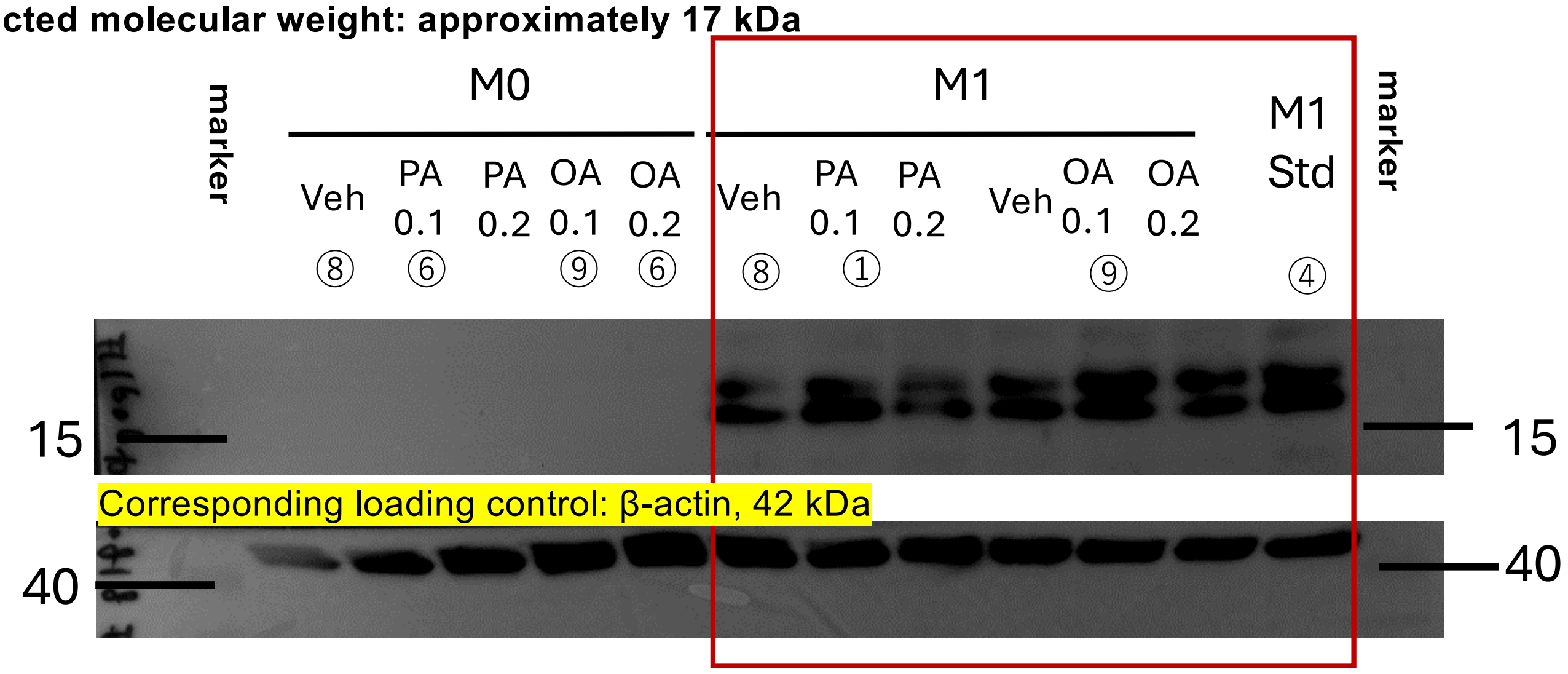

Blot ID: #1315
Target: TNF-α
Expected molecular weight: approximately 17 kDa

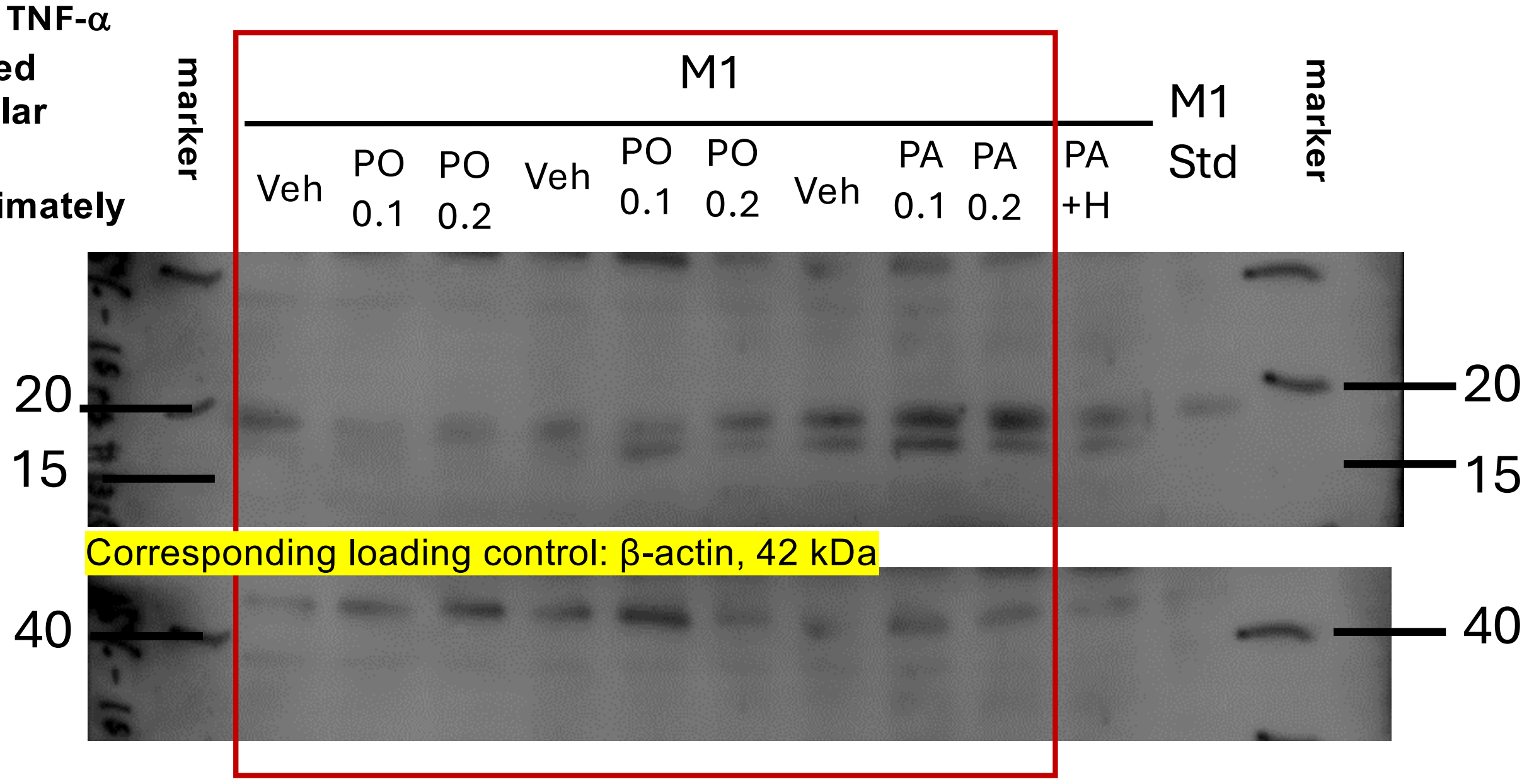

Blot ID: #152
Target: TNF-α
Expected molecular weight: approximately 17 kDa

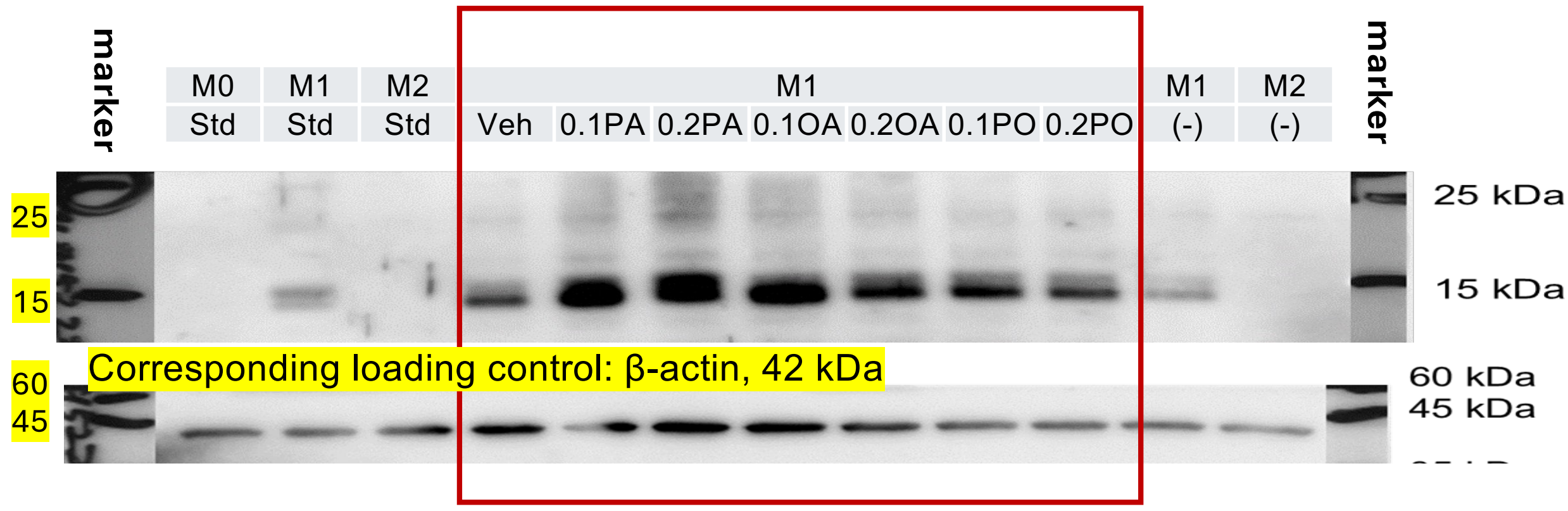

Blot ID: #1304
Target: TNF-α
Expected molecular weight: approximately 17 kDa

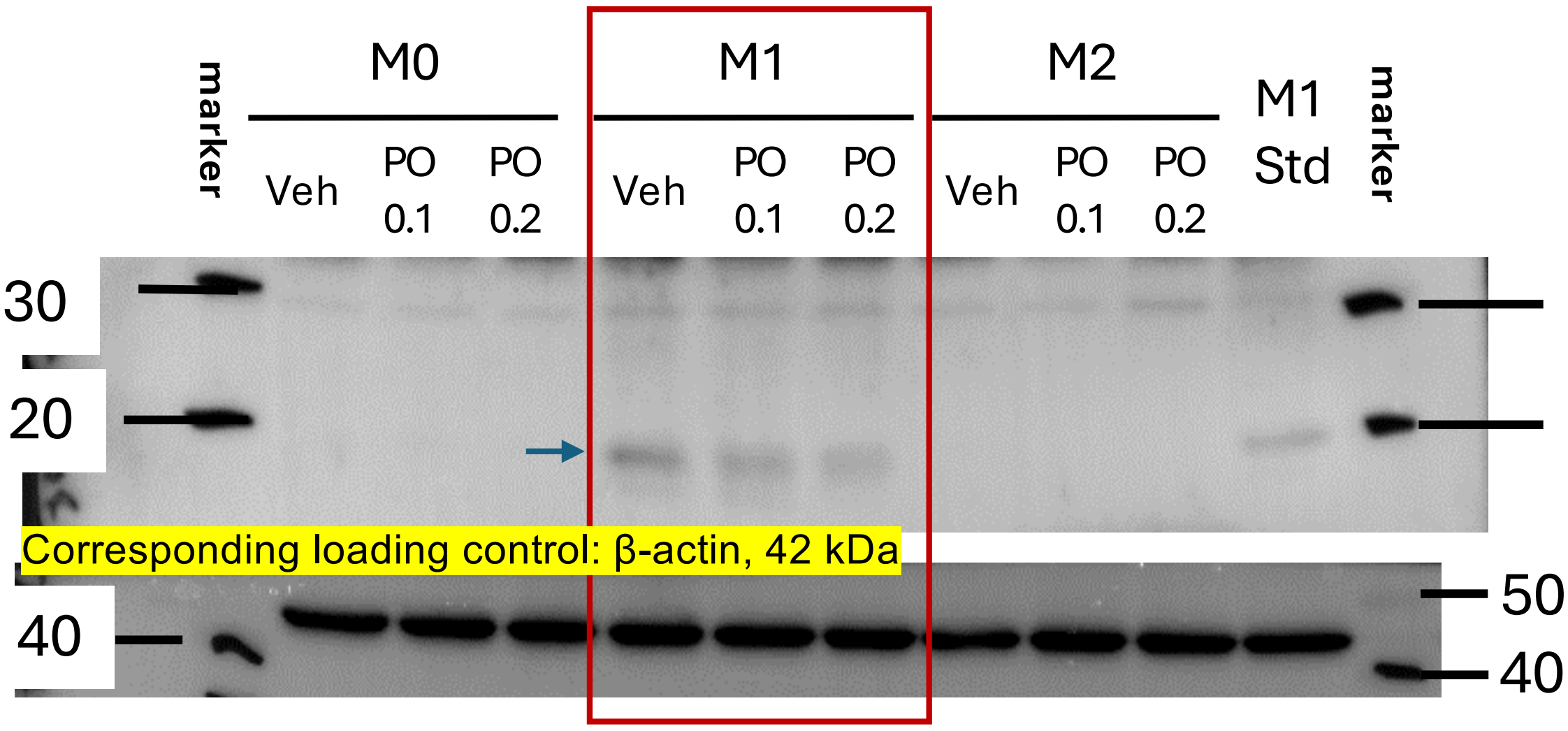

Blot ID: #176
Target: TNF-α
Expected molecular weight: approximately 17 kDa

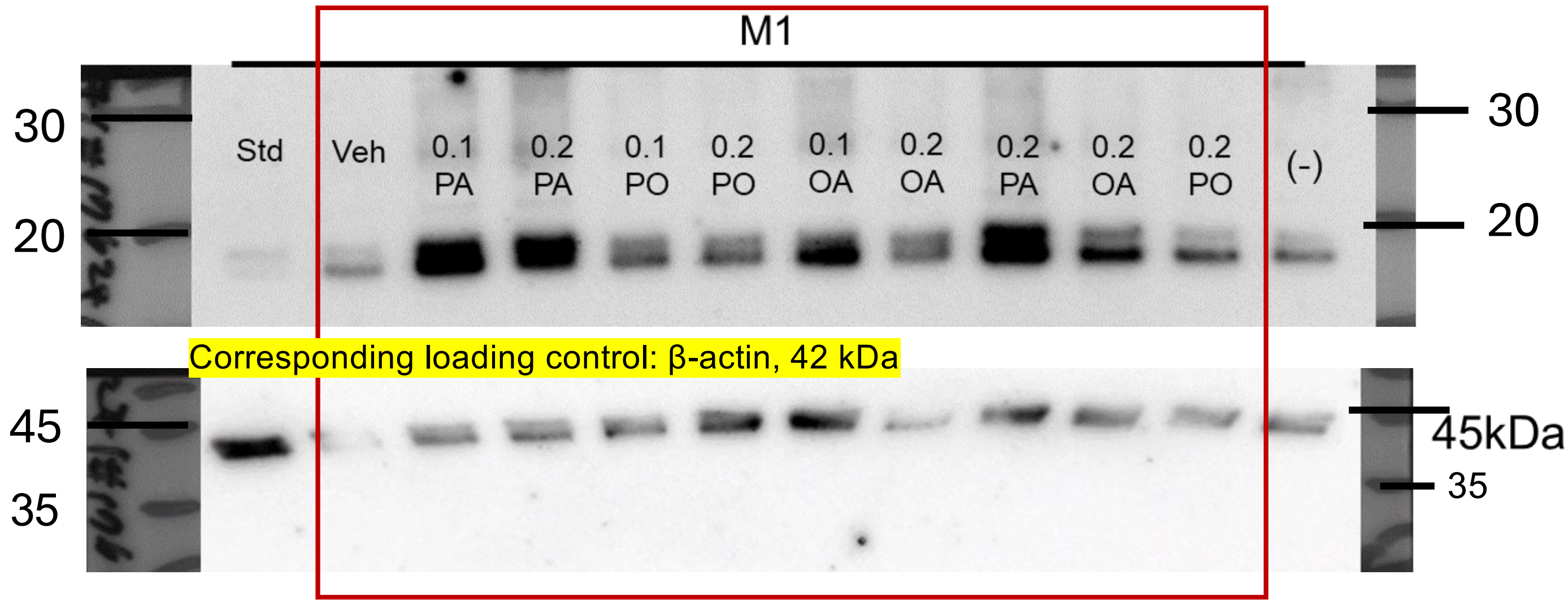

Blot ID: #175
Target: TNF-α
Expected molecular weight: approximately 17 kDa

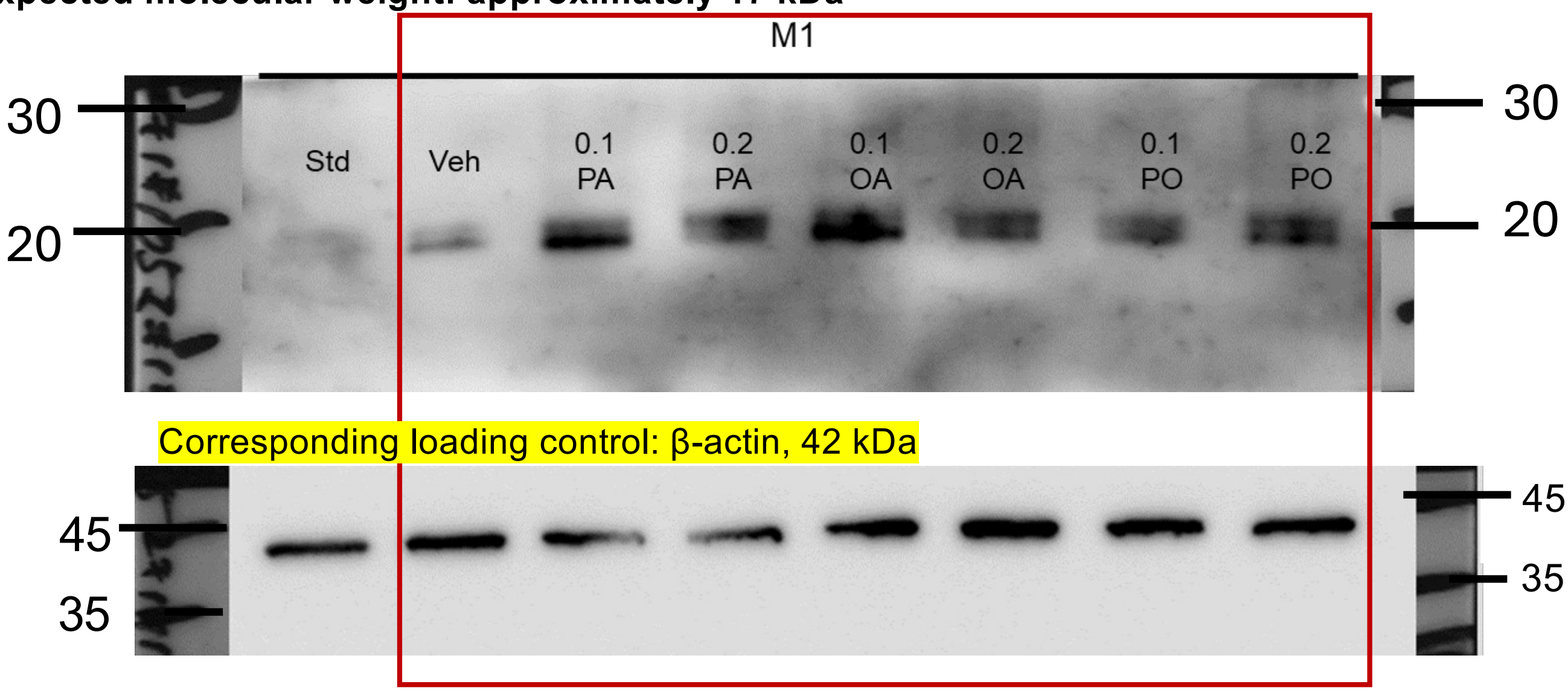

Fig. 2d PPAR-γ

Blot ID: #1205
Target: PPAR-γ
Expected molecular weight: approximately 53, 58 kDa

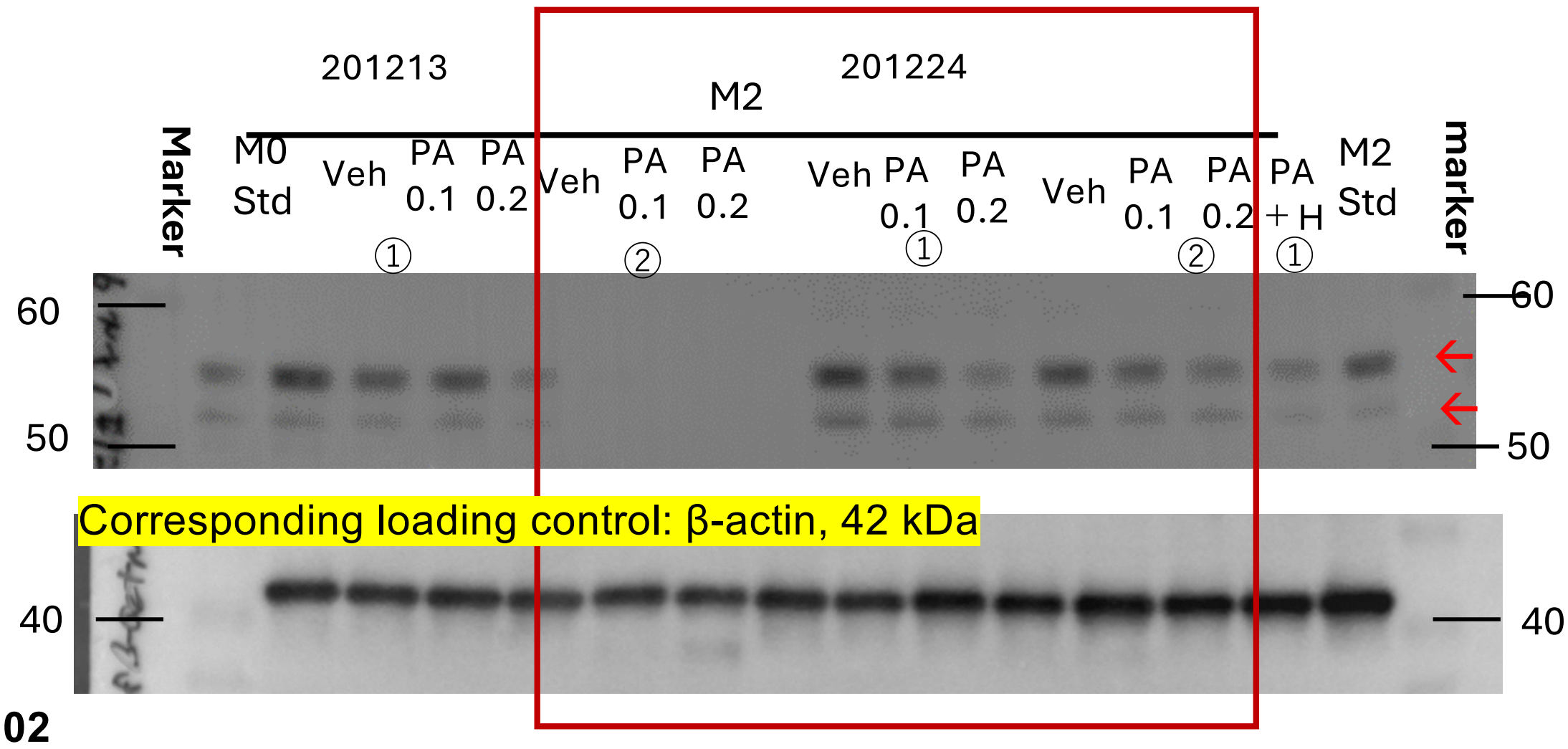

Blot ID: #1102
Target: PPAR-γ
Expected molecular weight: approximately 53, 58 kDa
The corresponding β-actin loading control was obtained from a separately run gel and membrane using aliquots of the same samples.

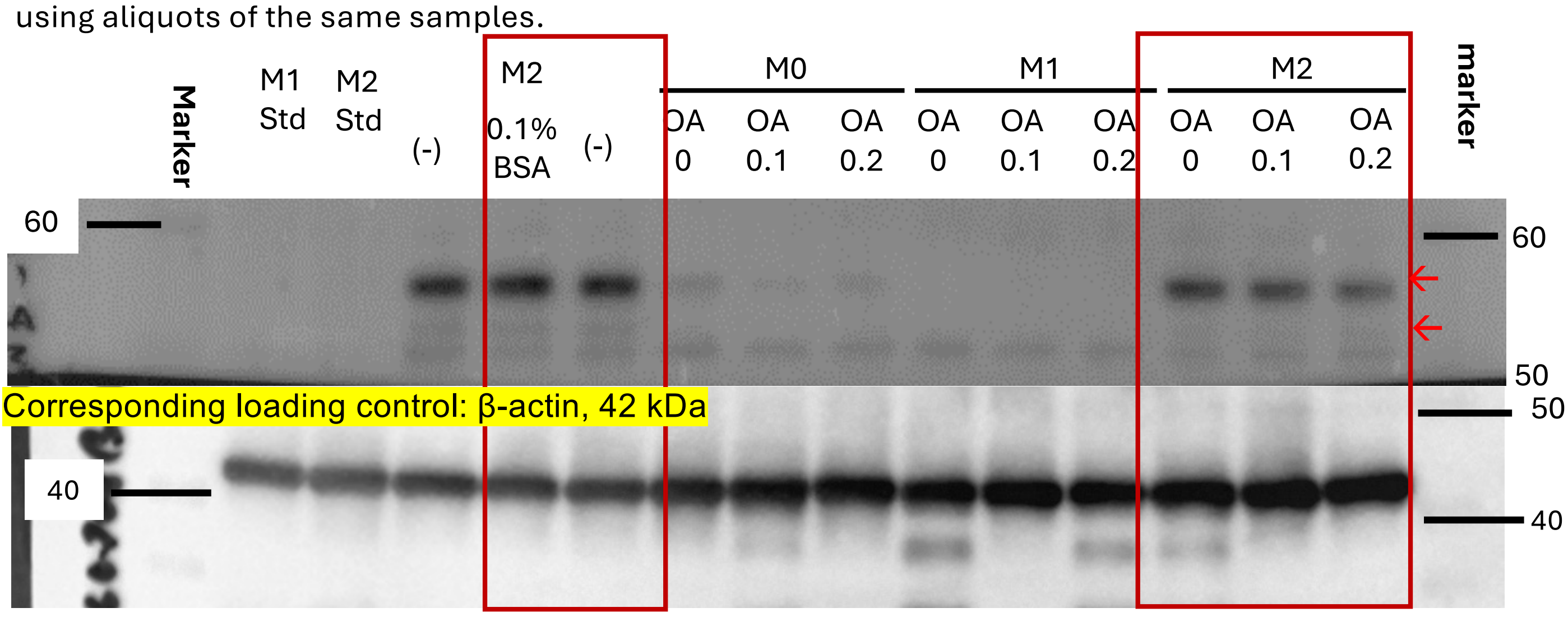

Blot ID: #1306
Target: PPAR-γ
Expected molecular weight: approximately 53, 58 kDa

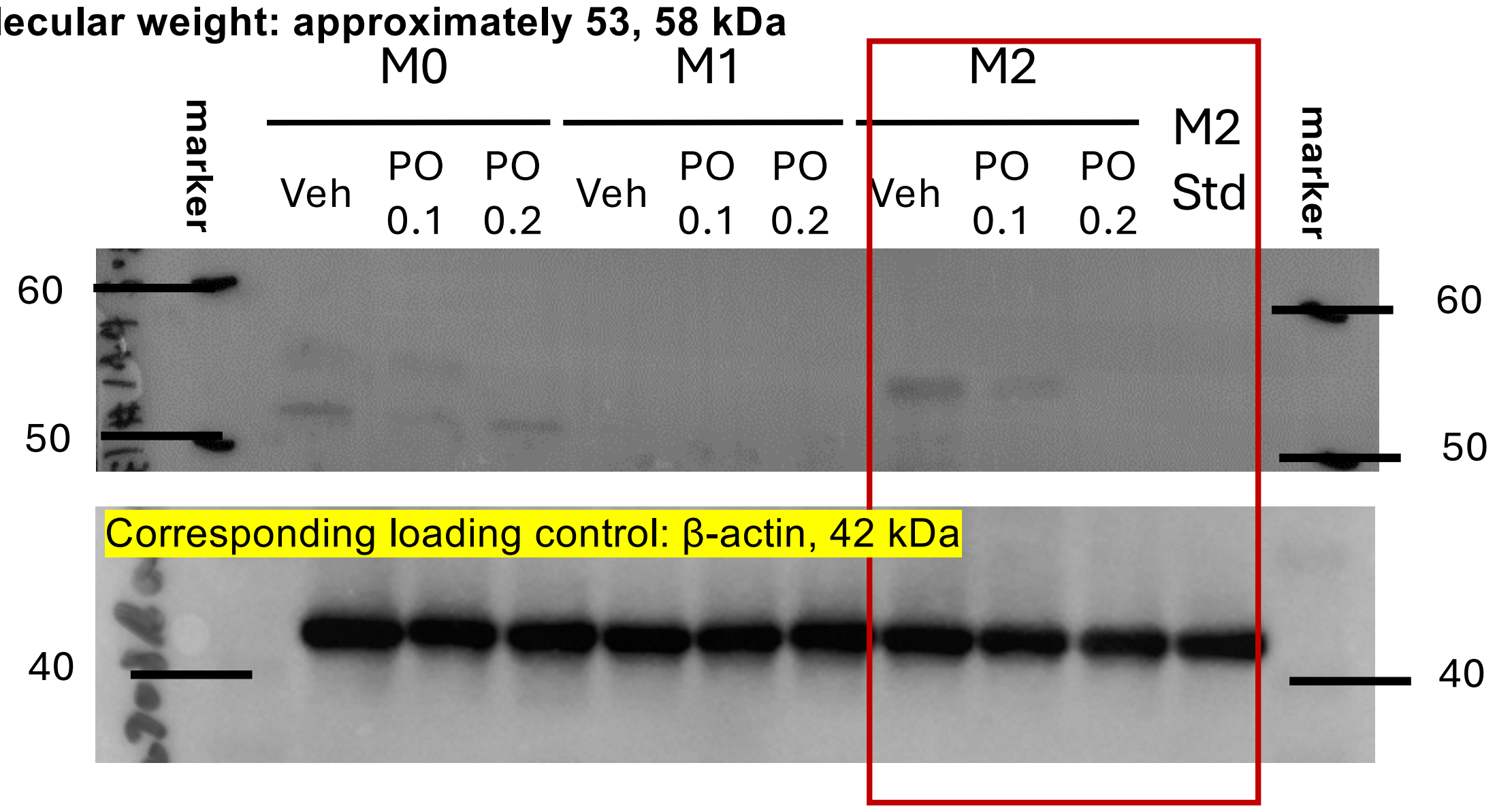

Blot ID: #1201
Target: PPAR-γ
Expected molecular weight: approximately 53, 58 kDa

The corresponding β-actin loading control was obtained from a separately run gel and membrane using aliquots of the same samples.

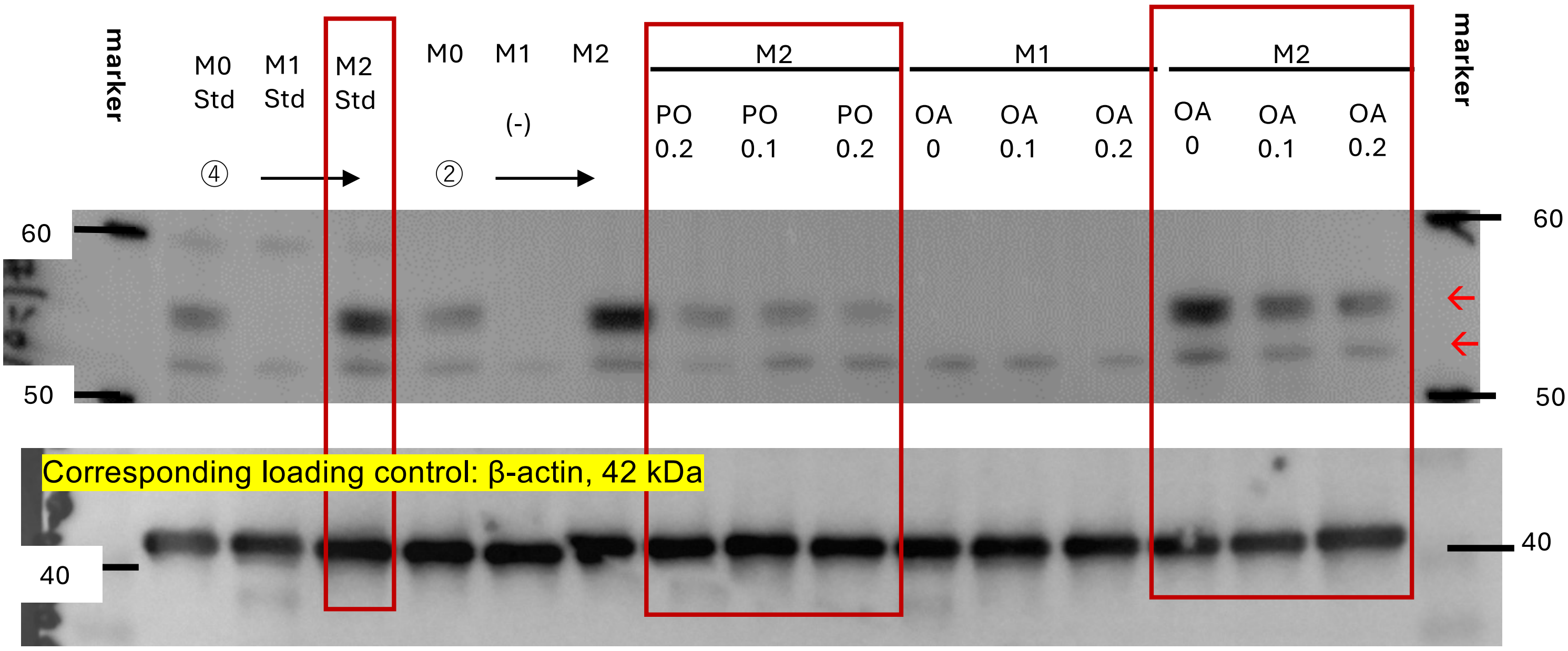

Fig. 2e MR

Blot ID: #1602
Target: MR
Expected molecular weight: approximately 190 kDa

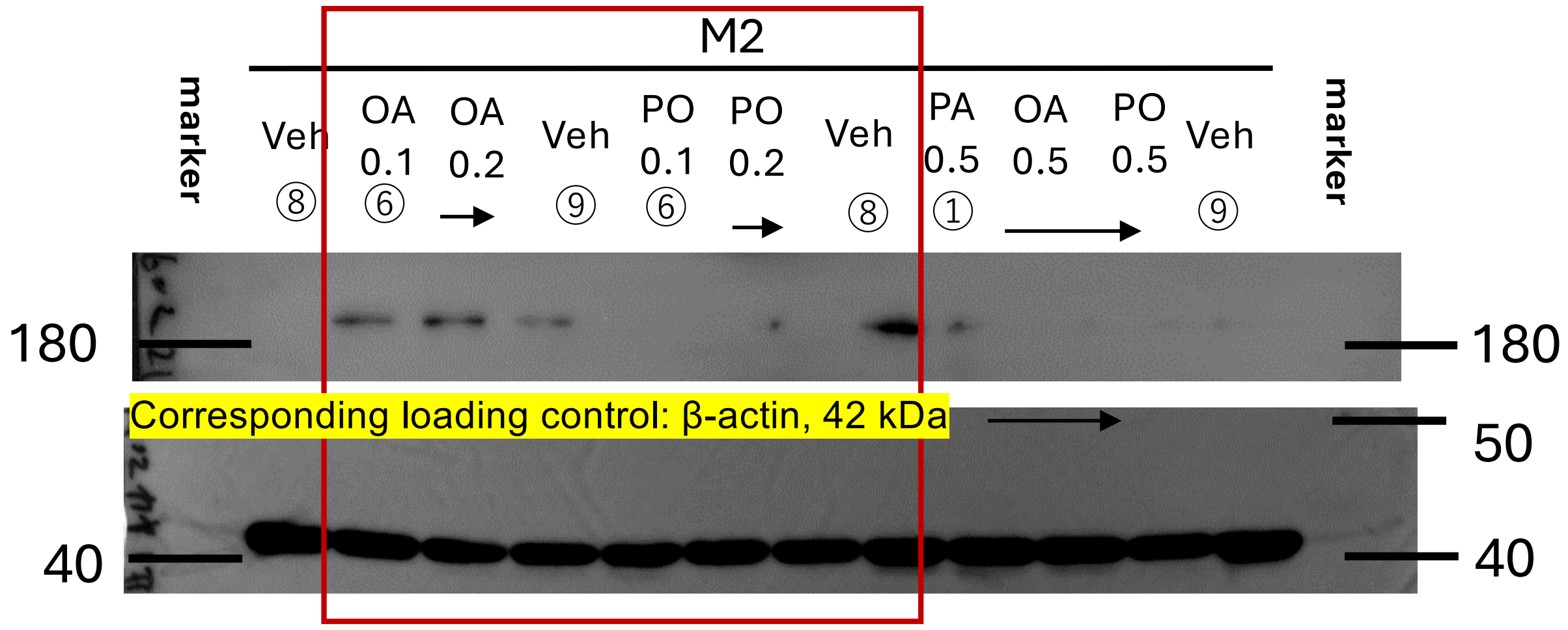

Blot ID: #1201
Target: MR
Expected molecular weight: approximately 190 kDa

The corresponding β-actin loading control was obtained from a separately run gel and membrane using aliquots of the same samples.

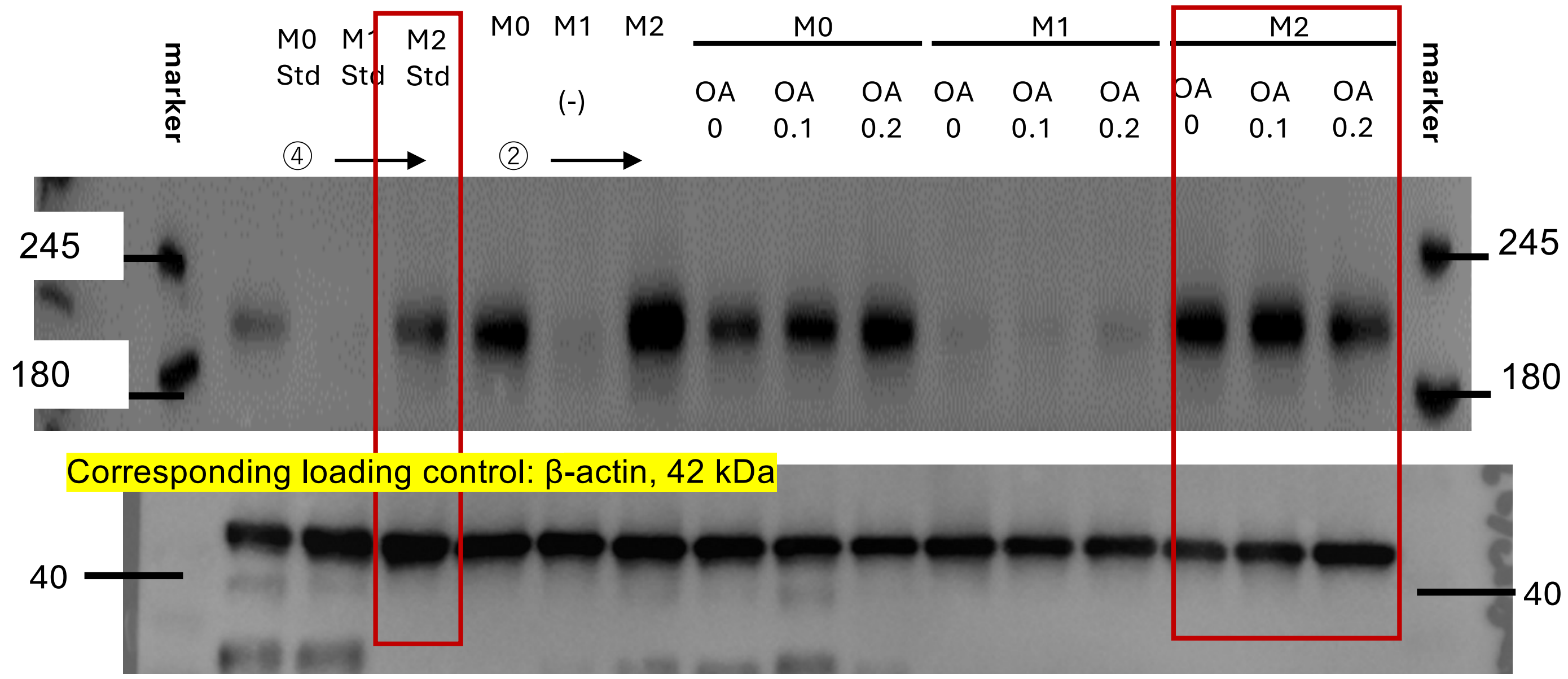

Blot ID: #154
Target: MR
Expected molecular weight: approximately 190 kDa

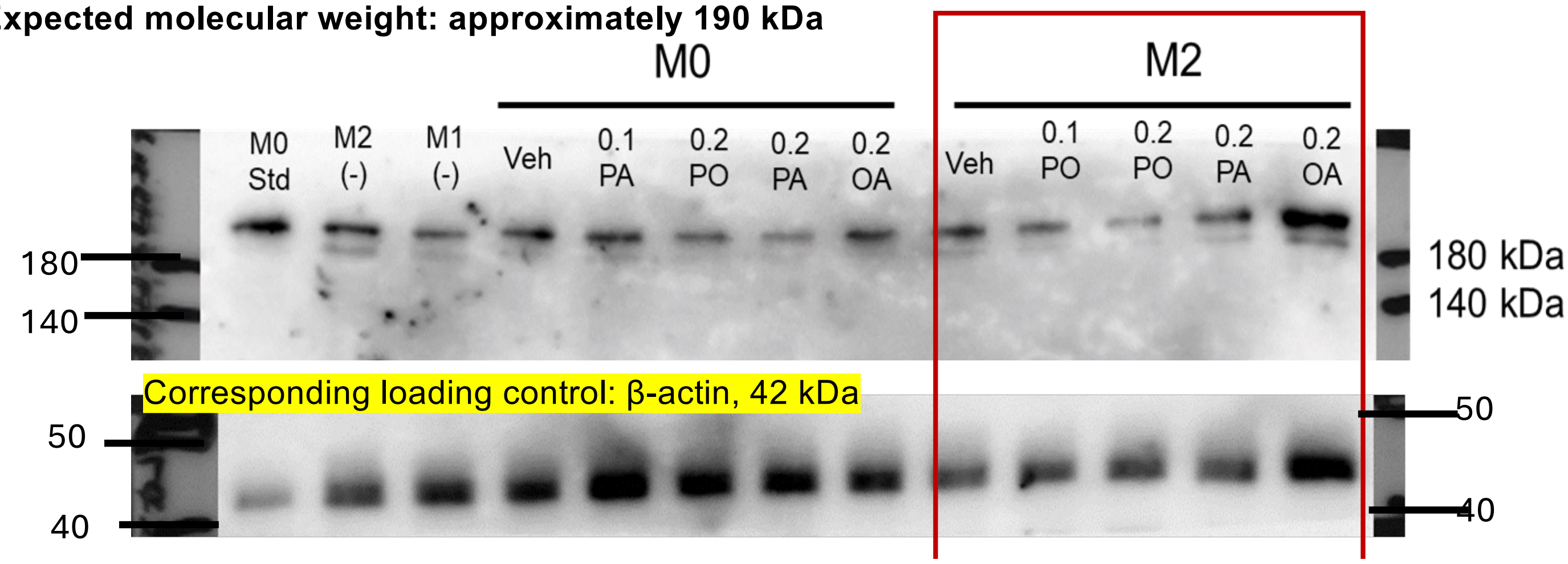

Blot ID: #1317
Target: MR
Expected molecular weight: approximately 190 kDa
The corresponding β-actin loading control was obtained from a separately run gel and membrane using aliquots of the same samples.

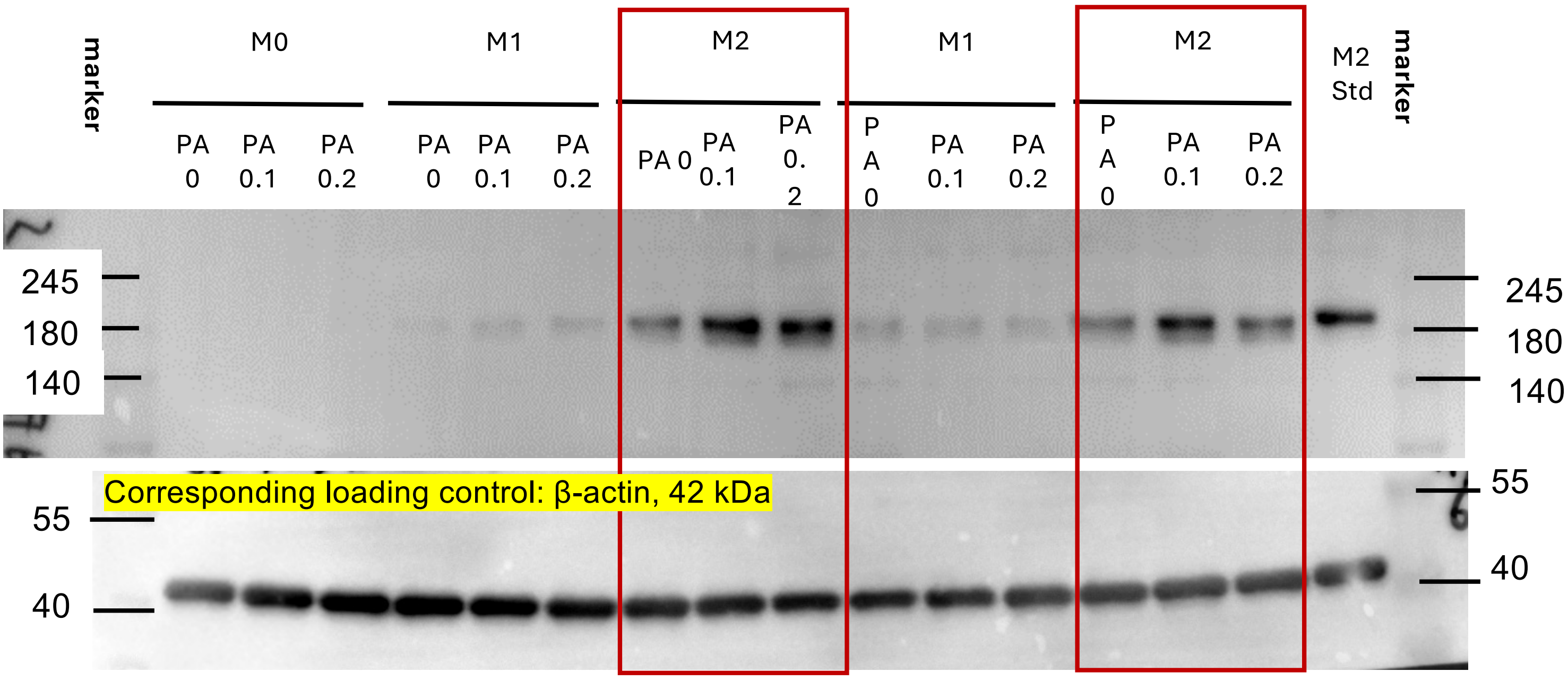

Blot ID: #1307
Target: MR
Expected molecular weight: approximately 190 kDa
The corresponding β-actin loading control was obtained from a separately run gel and membrane using aliquots of the same samples.

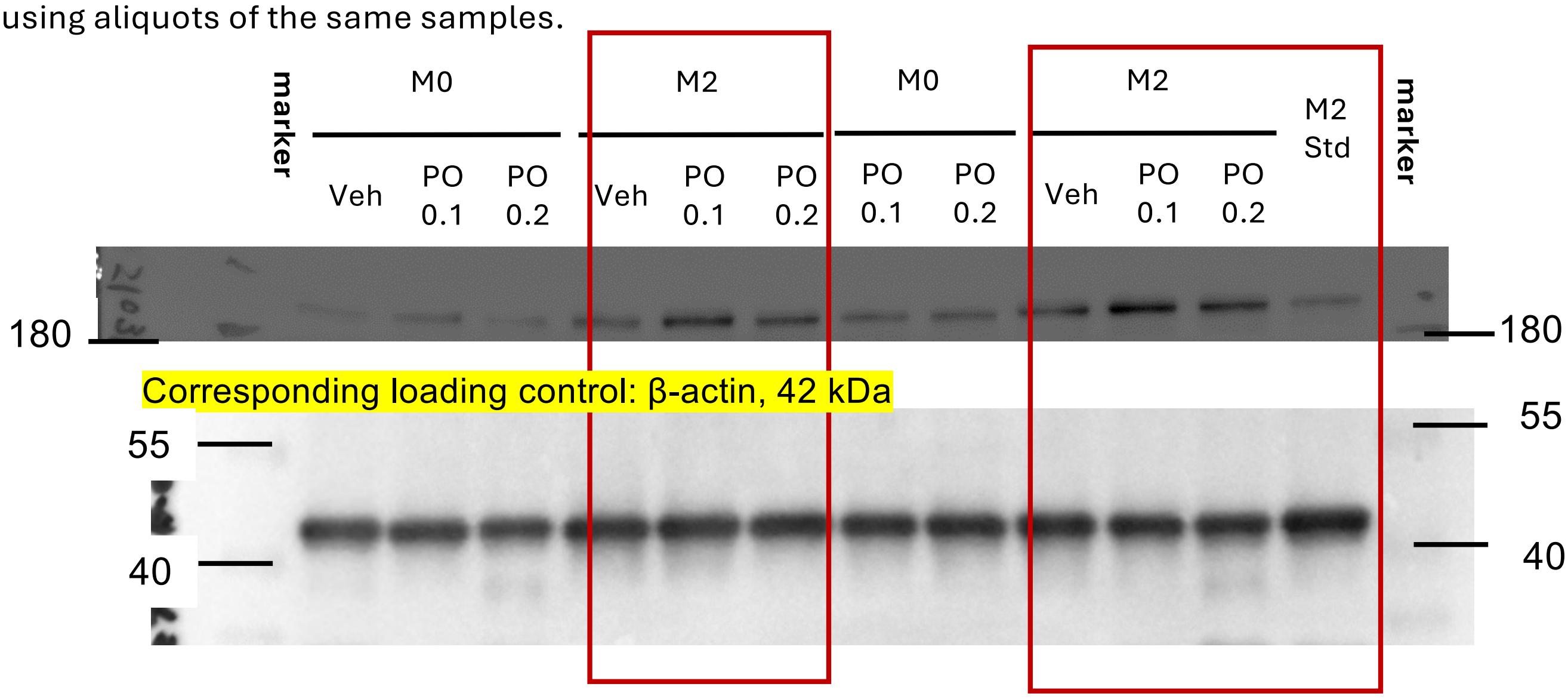

Fig. 4e α-SMA

Blot ID: #801
Target: α-SMA
Expected molecular weight: approximately 42 kDa

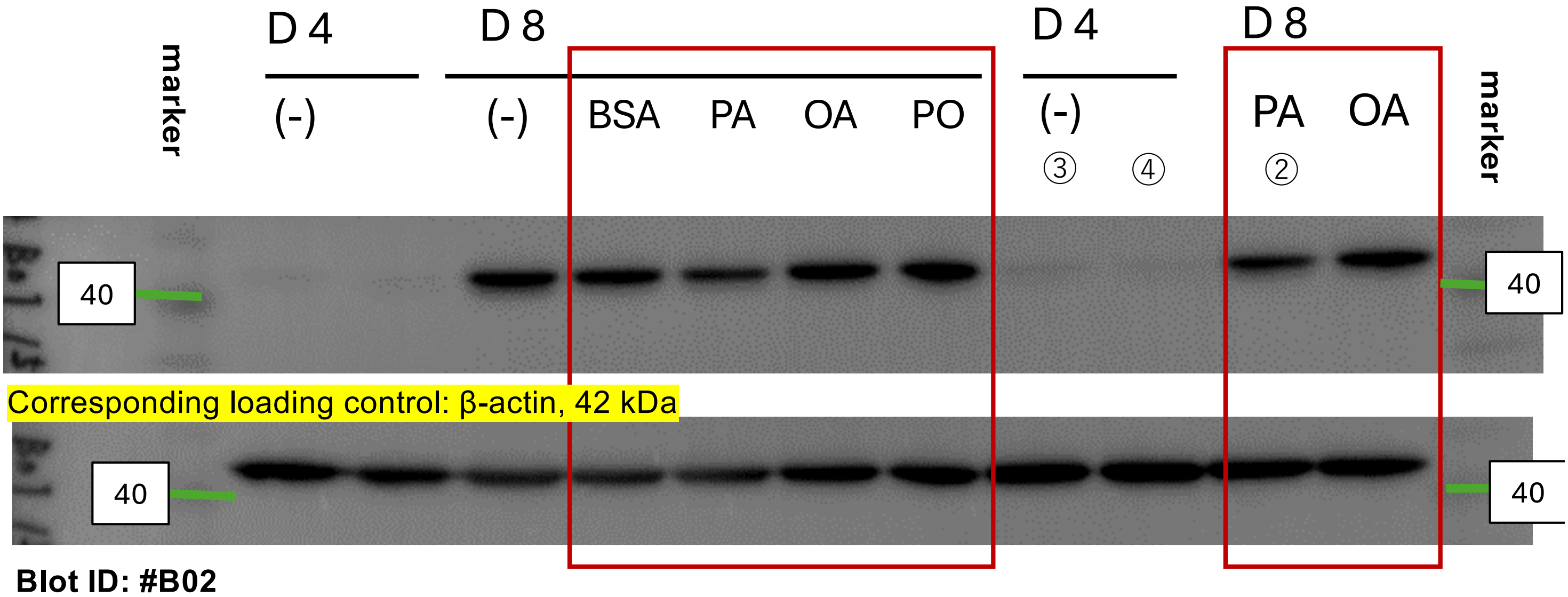

Blot ID: #B02
Target: α-SMA
Expected molecular weight: approximately 42 kDa

The corresponding β-actin loading control was obtained from a separately run gel and membrane using aliquots of the same samples.

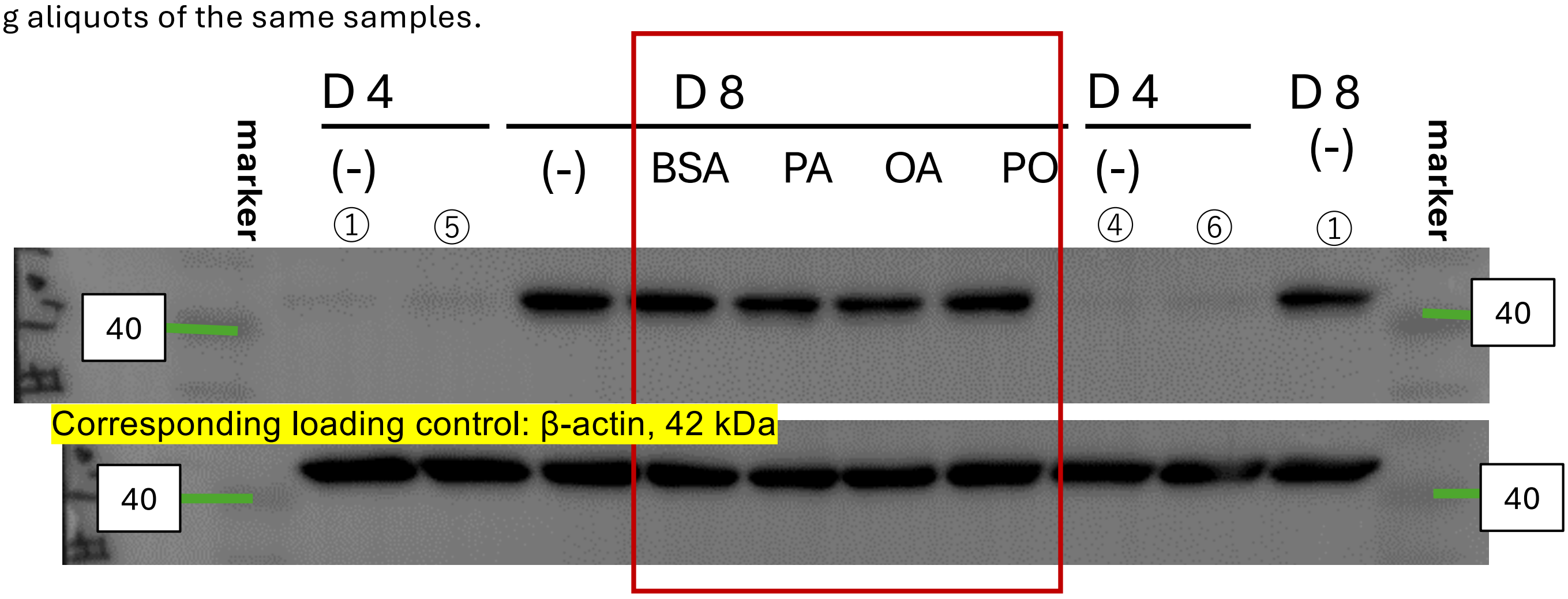

Blot ID: #C01
Target: α-SMA
Expected molecular weight: approximately 42 kDa

The corresponding β-actin loading control was obtained from a separately run gel and membrane using aliquots of the same samples.

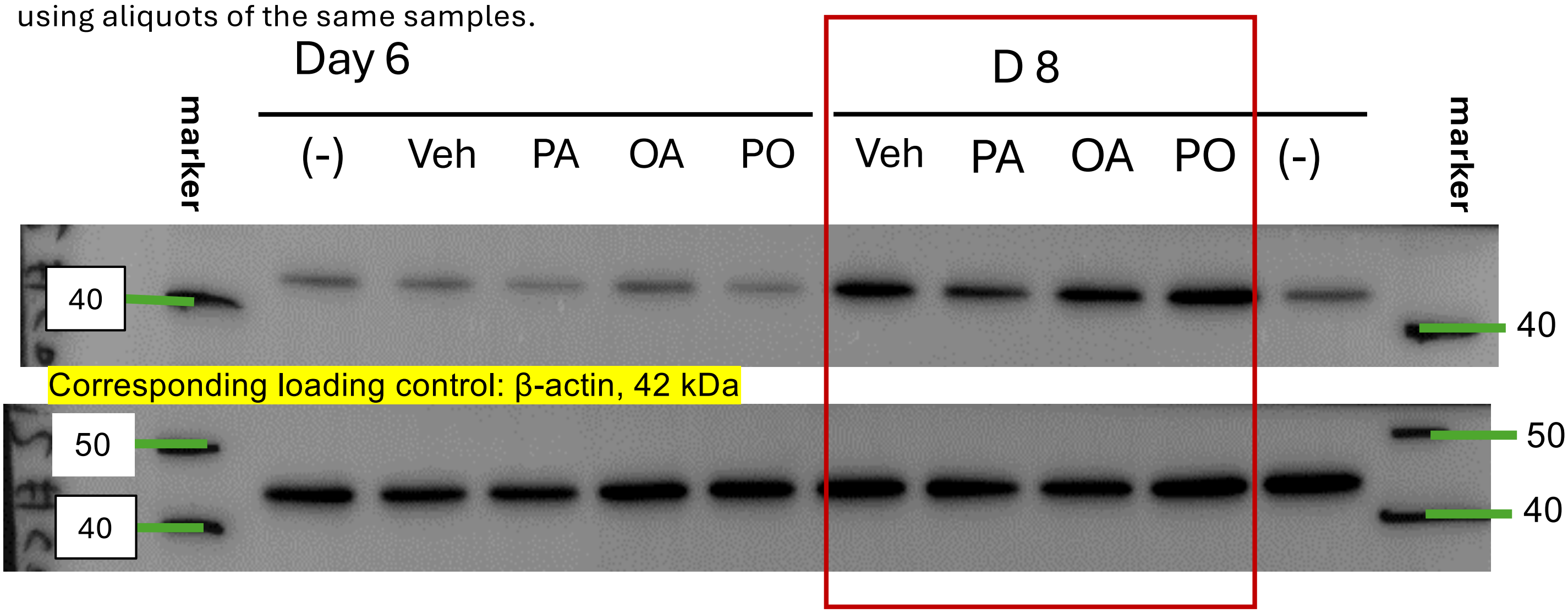

Fig. 4f Cytoglobin

Blot ID: #B01
Target: Cytoglobin
Expected molecular weight: approximately 21 kDa

The corresponding β-actin loading control was obtained from a separately run gel and membrane using aliquots of the same samples.

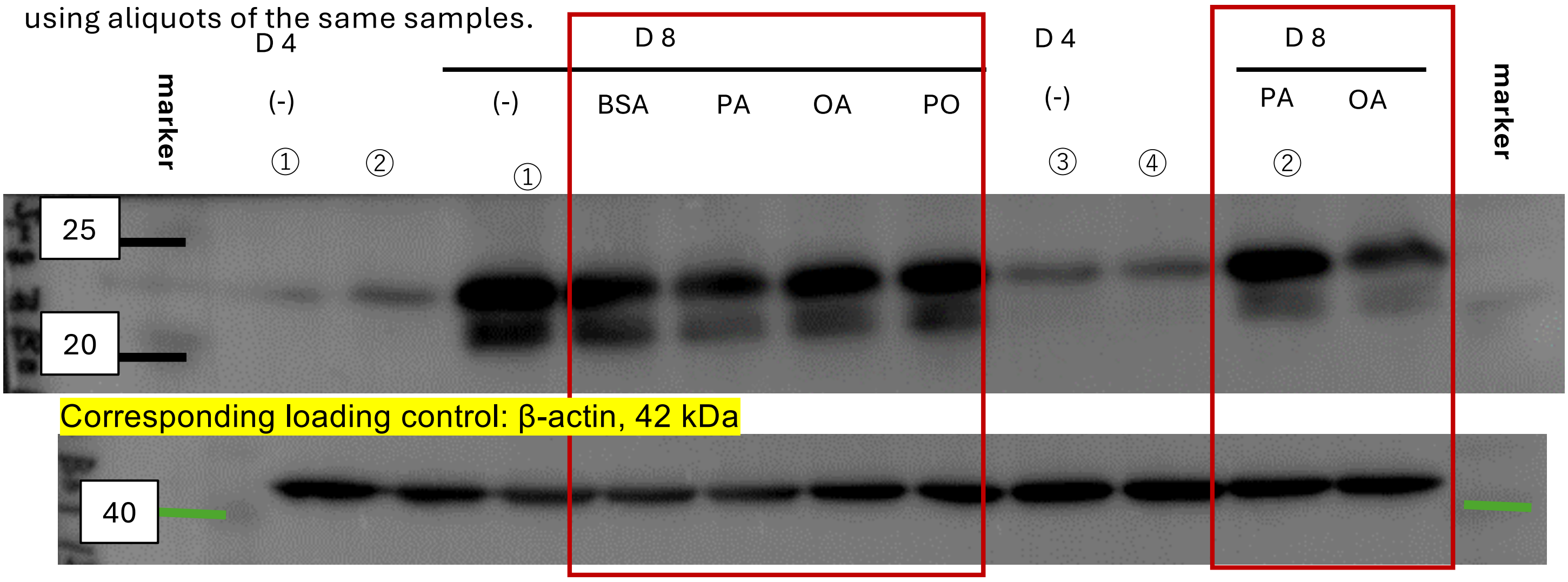

Blot ID: #B02
Target: Cytoglobin
Expected molecular weight: approximately 21 kDa

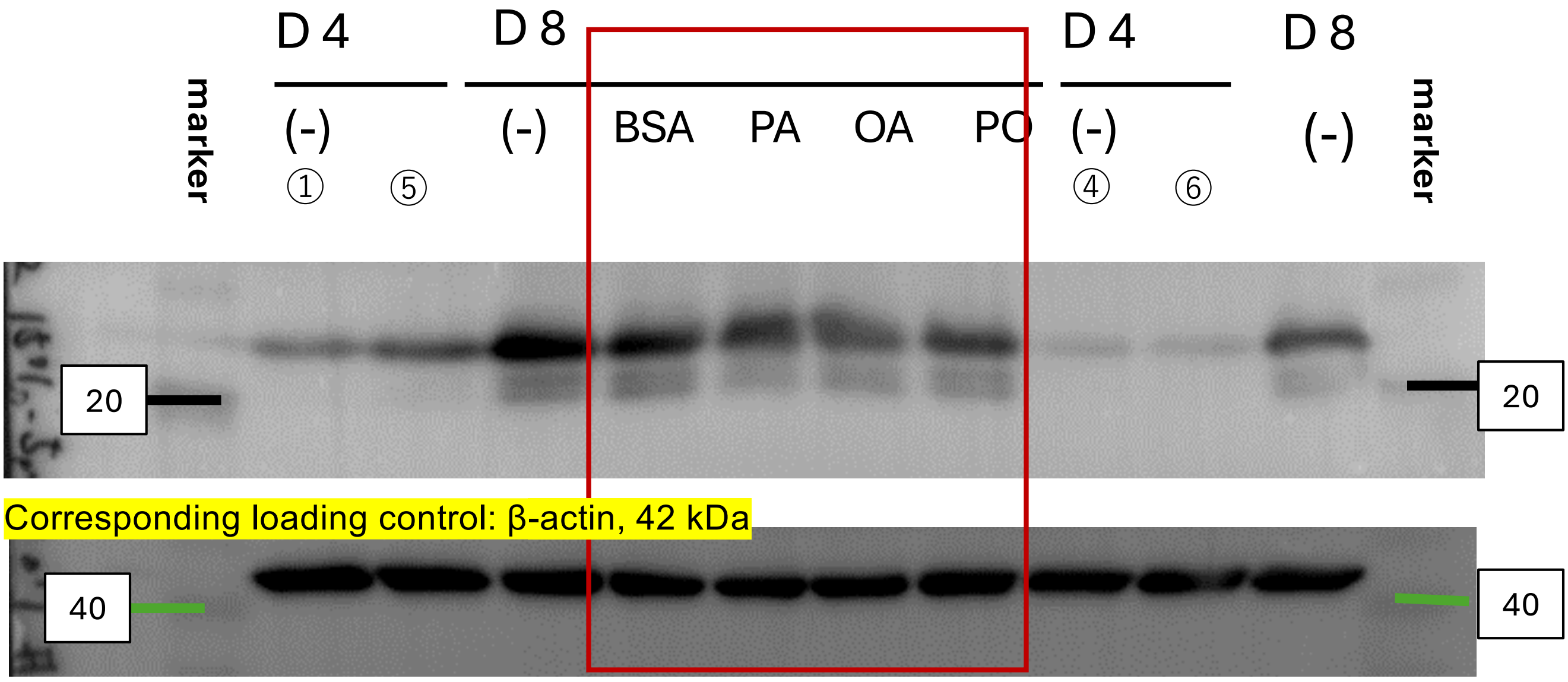

Blot ID: #B03
Target: Cytoglobin
Expected molecular weight: approximately 21 kDa

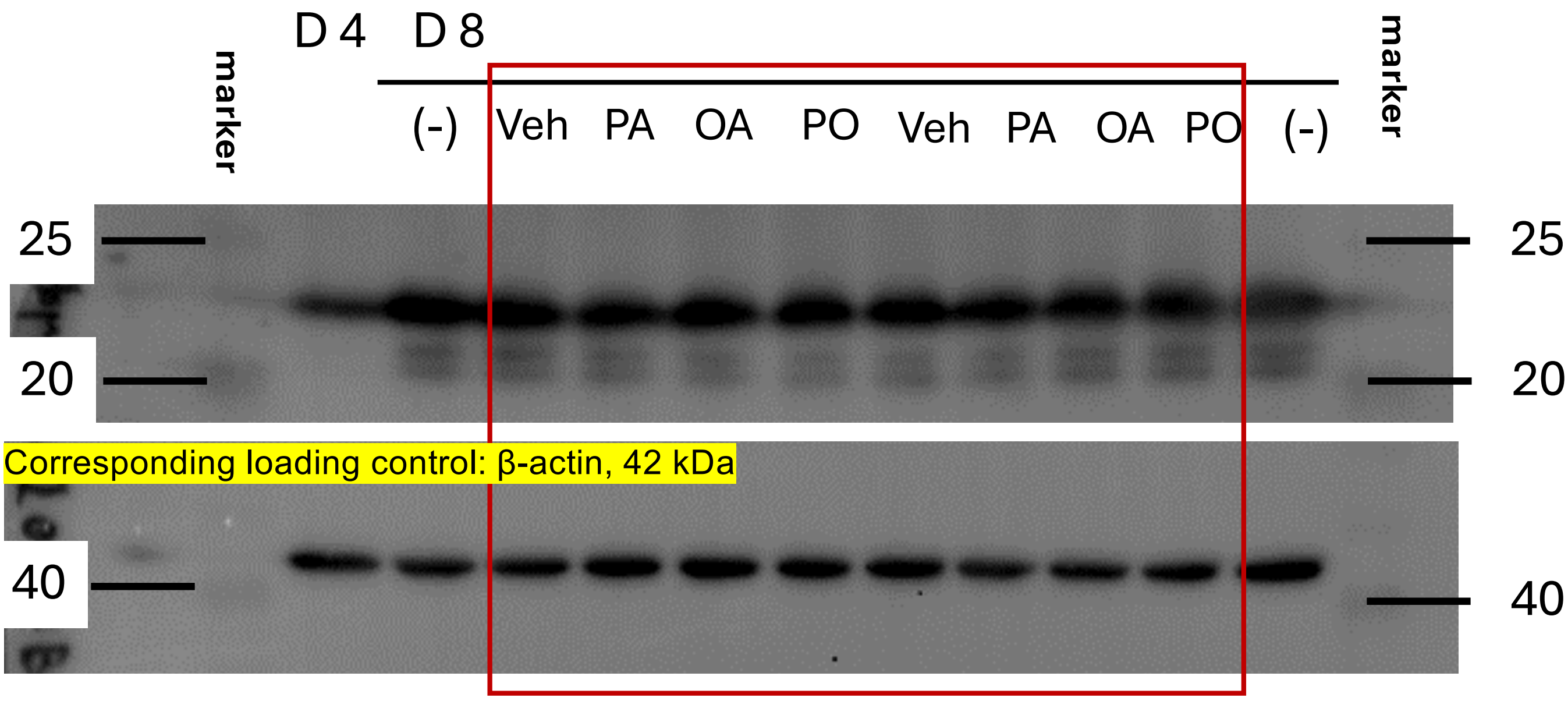

Blot ID: #C01
Target: Cytoglobin
Expected molecular weight: approximately 21 kDa

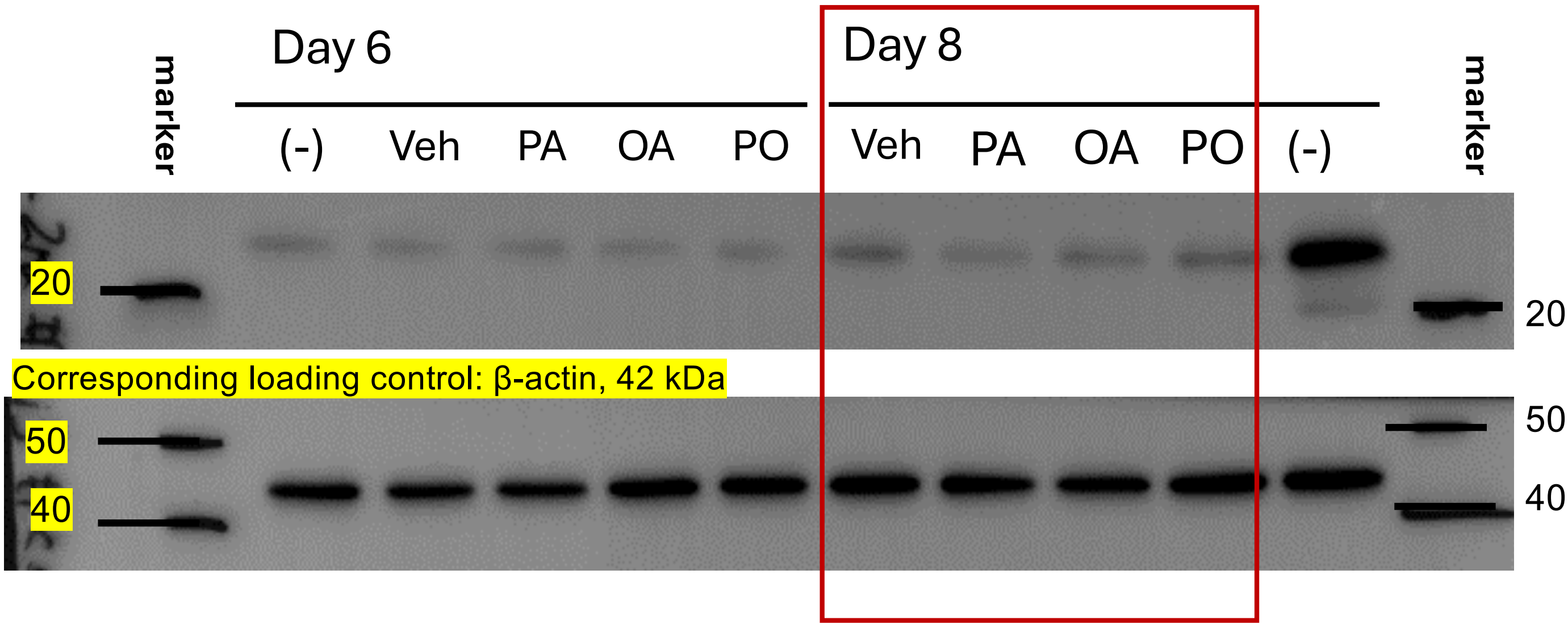

Fig. 6a α-SMA

Blot ID: #H02
Target: α-SMA
Expected molecular weight: approximately 42 kDa

The corresponding β-actin loading control was obtained from a separately run gel and membrane using aliquots of the same samples.

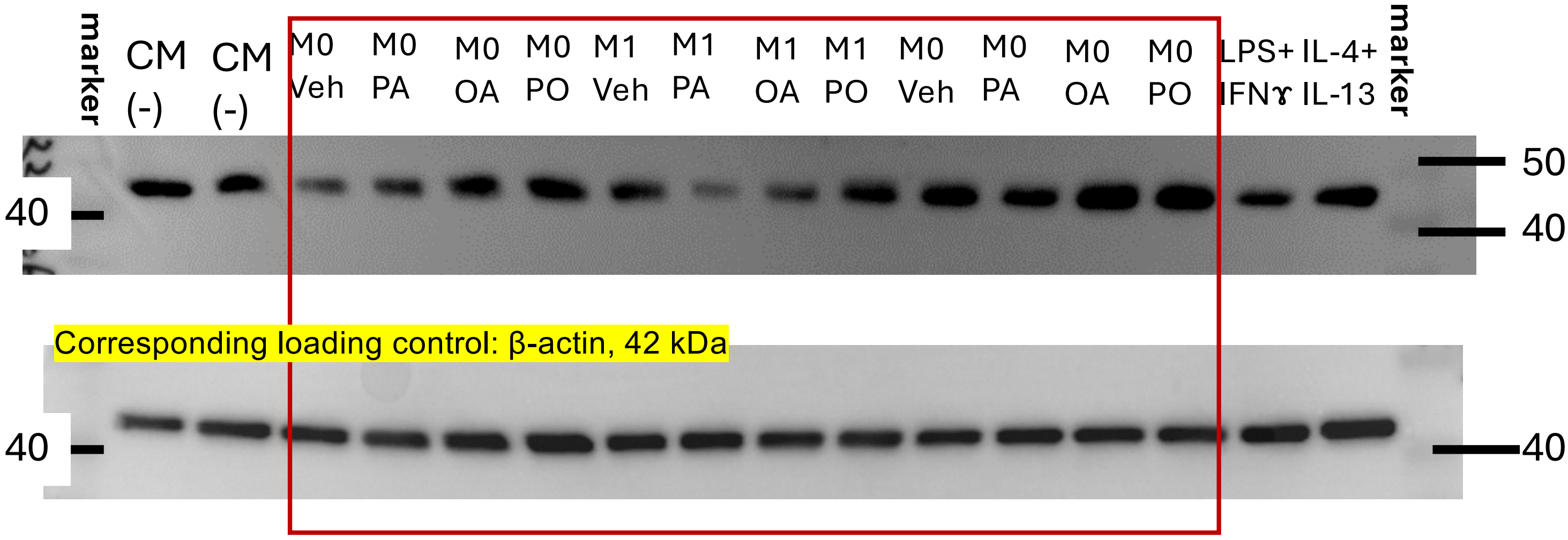

Blot ID: #E01
Target: α-SMA
Expected molecular weight: approximately 42 kDa

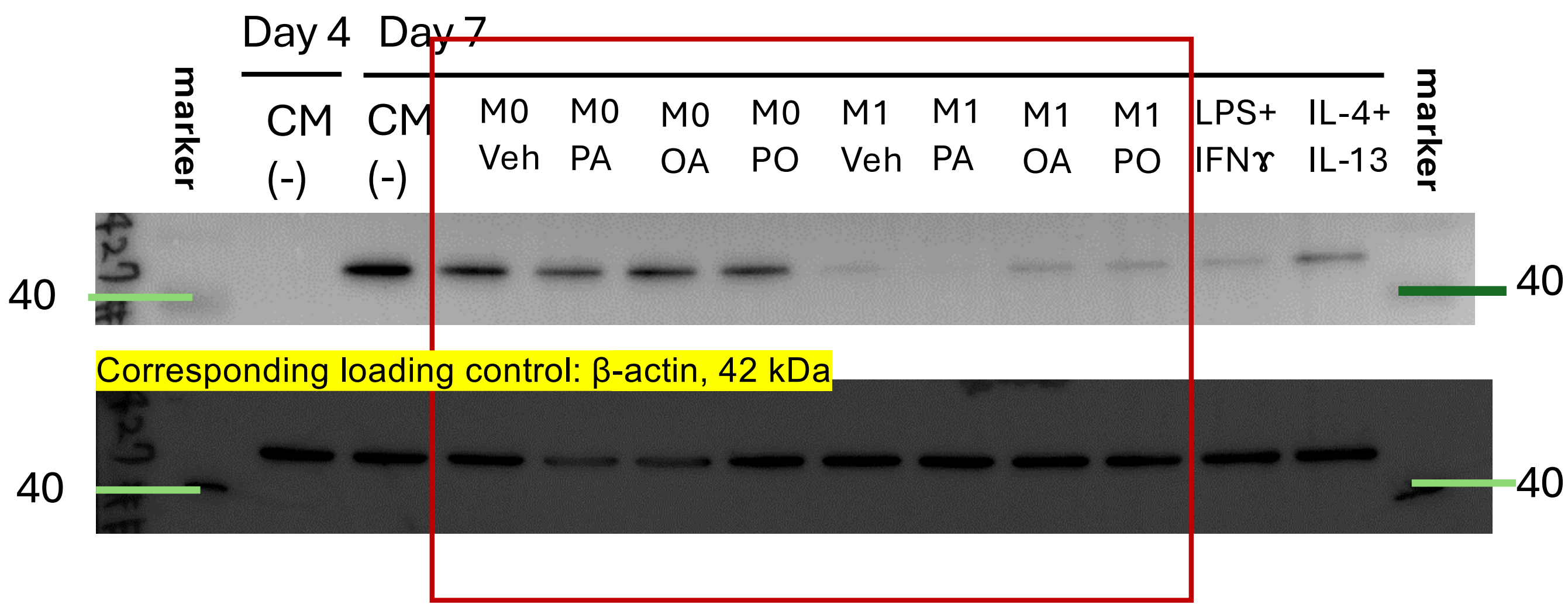

Blot ID: #E02
Target: α-SMA
Expected molecular weight: approximately 42 kDa

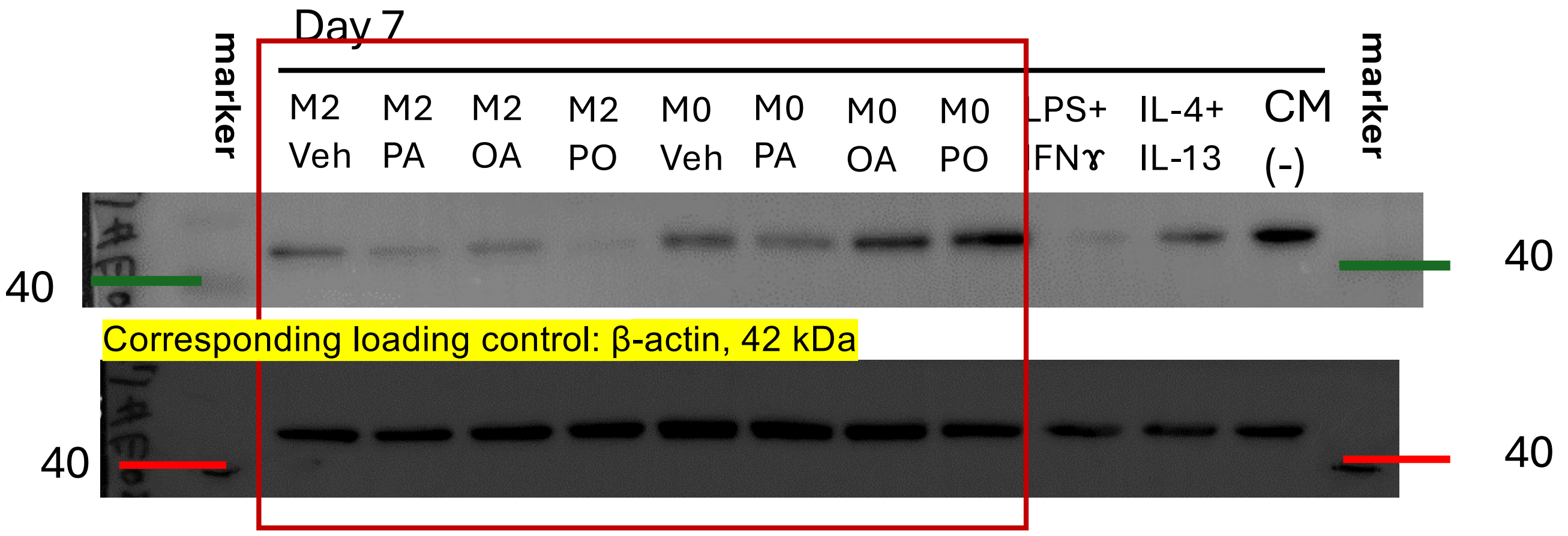

Blot ID: #E14
Target: α-SMA
Expected molecular weight: approximately 42 kDa

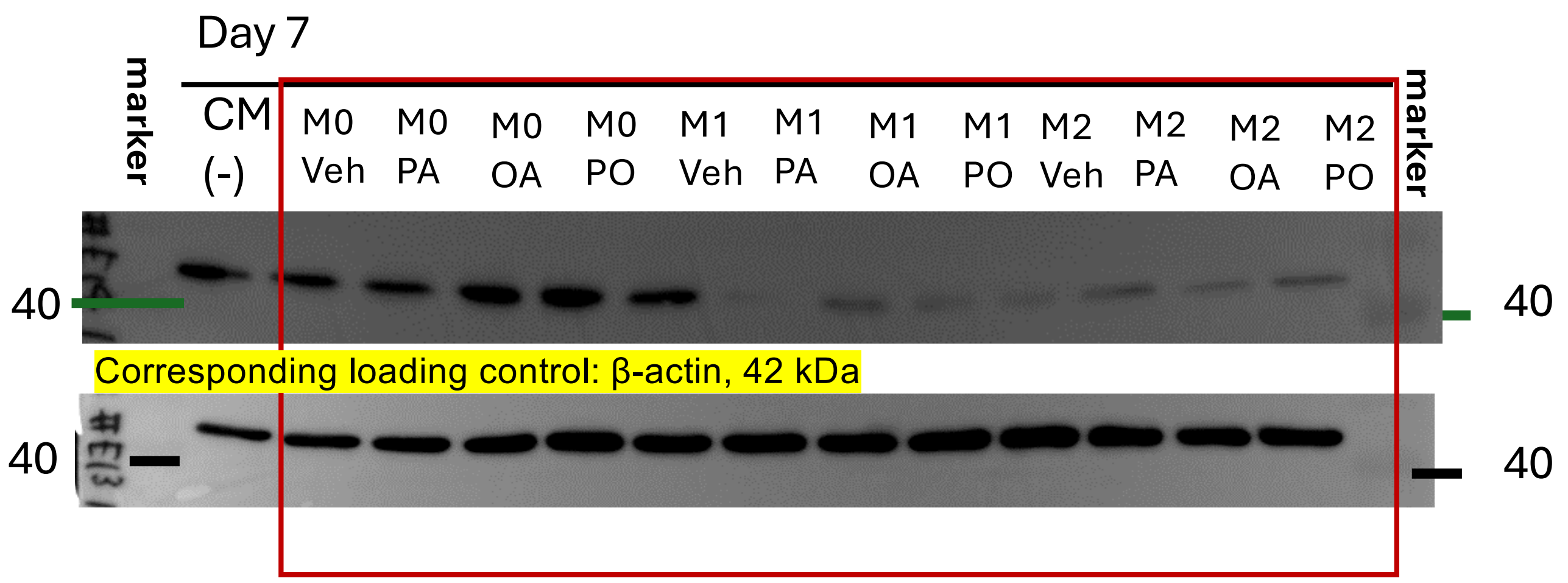

Fig. 6b cytoglobin

Blot ID: #F01
Target: Cytoglobin
Expected molecular weight: approximately 21 kDa
The corresponding β-actin loading control was obtained from a separately run gel and membrane using aliquots of the same samples.

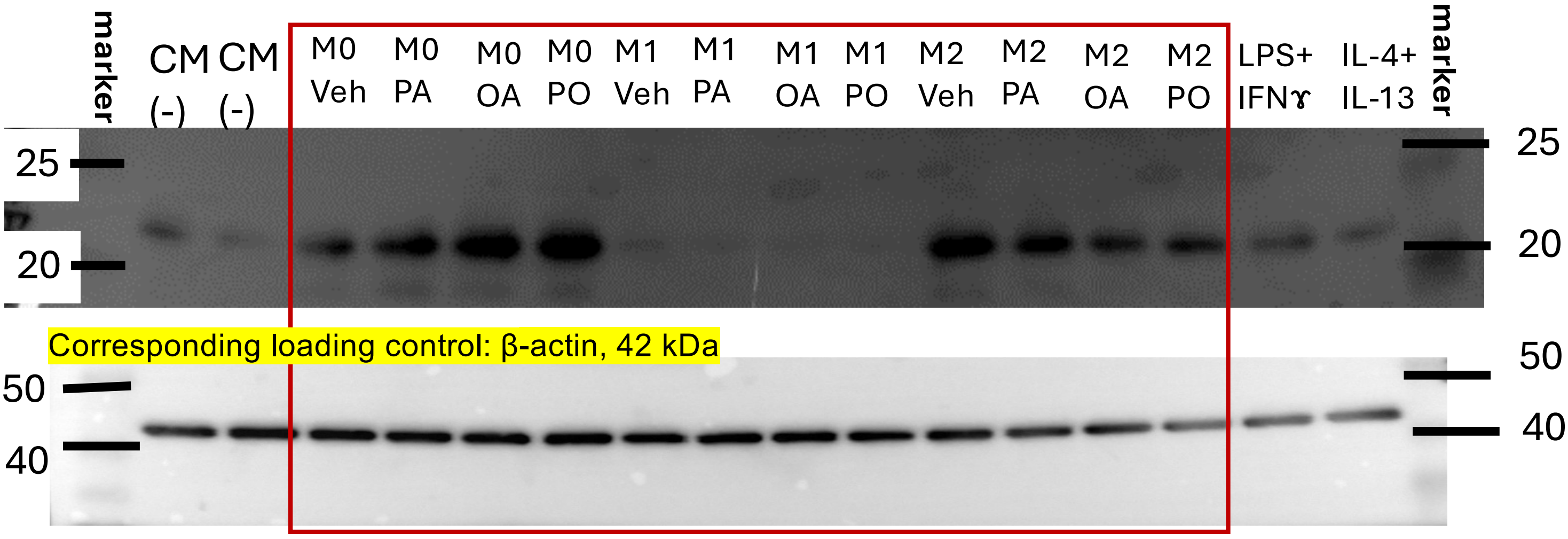

Blot ID: #F02
Target: Cytoglobin
Expected molecular weight: approximately 21 kDa

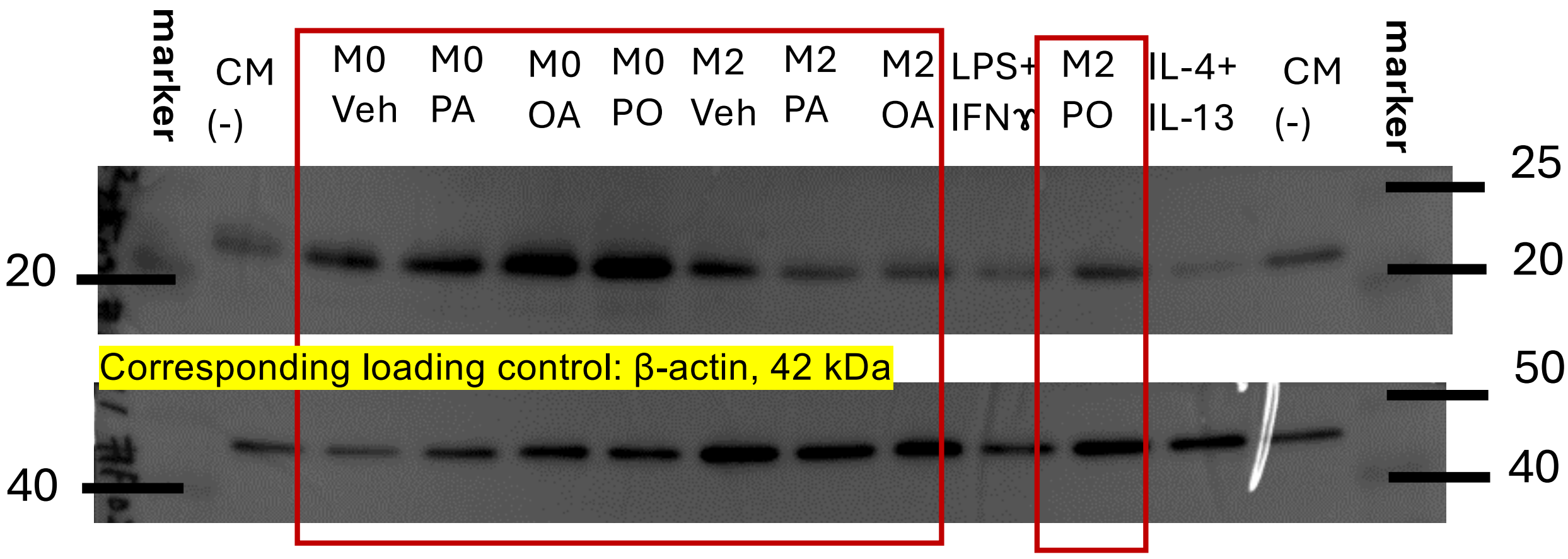

Blot ID: #H01
Target: Cytoglobin
Expected molecular weight: approximately 21 kDa

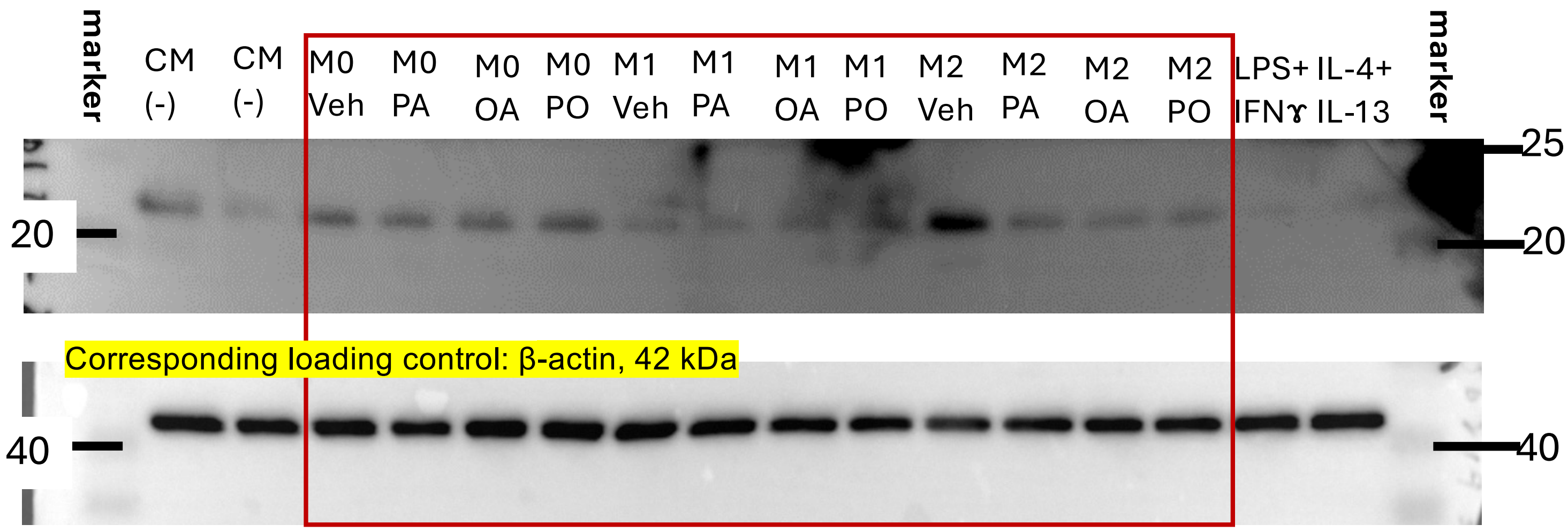

Blot ID: #E01
Target: Cytoglobin
Expected molecular weight: approximately 21 kDa

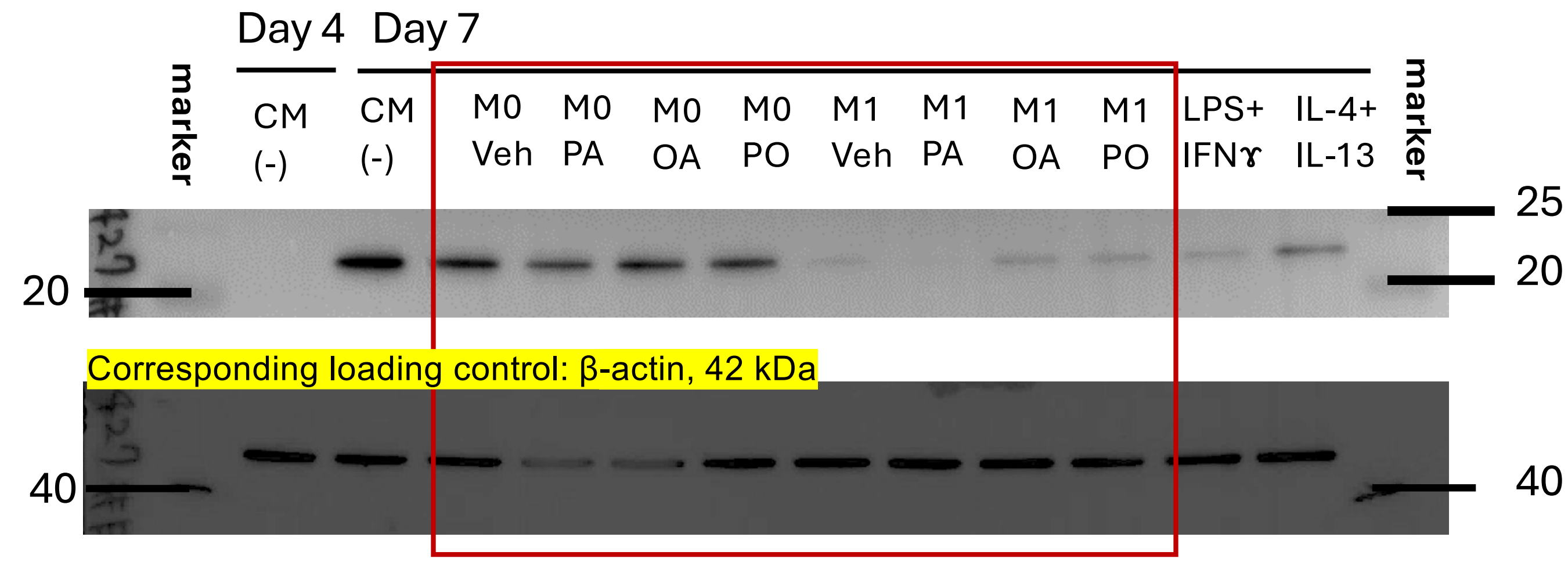

Blot ID: #E02
Target: Cytoglobin
Expected molecular weight: approximately 21 kDa

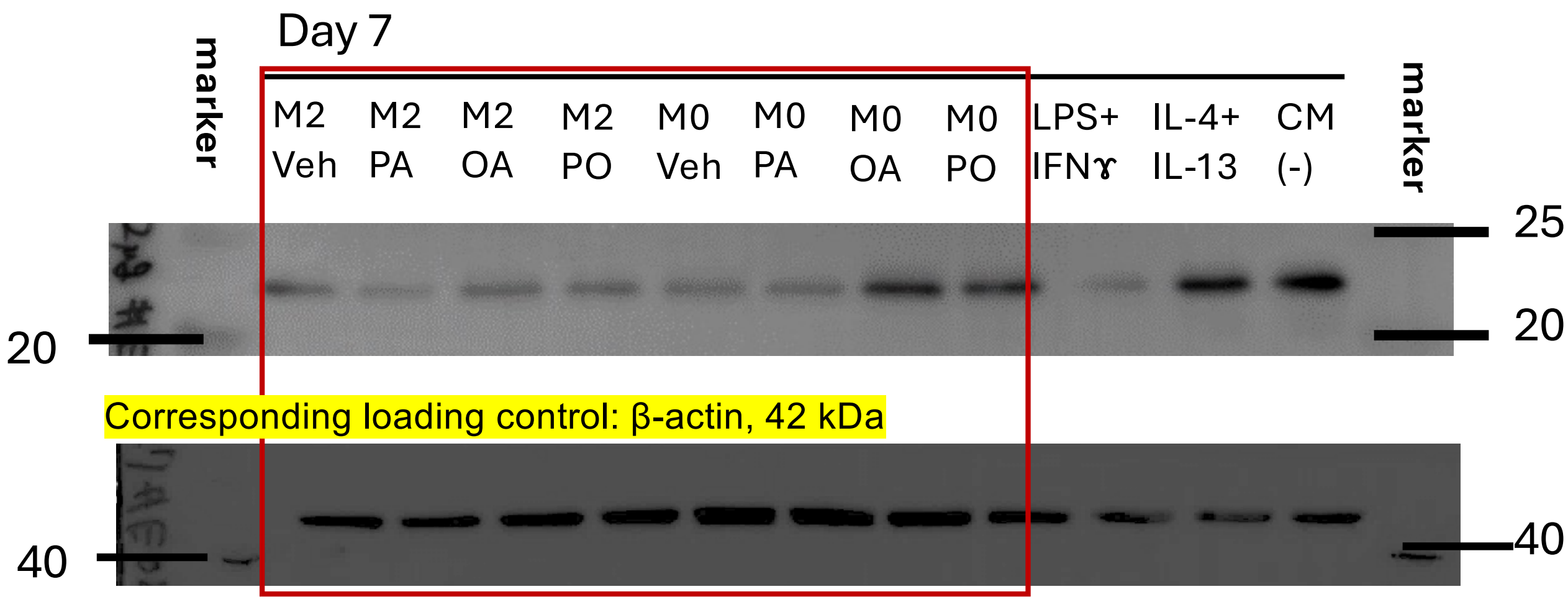

Blot ID: #E14
Target: Cytoglobin
Expected molecular weight: approximately 21 kDa

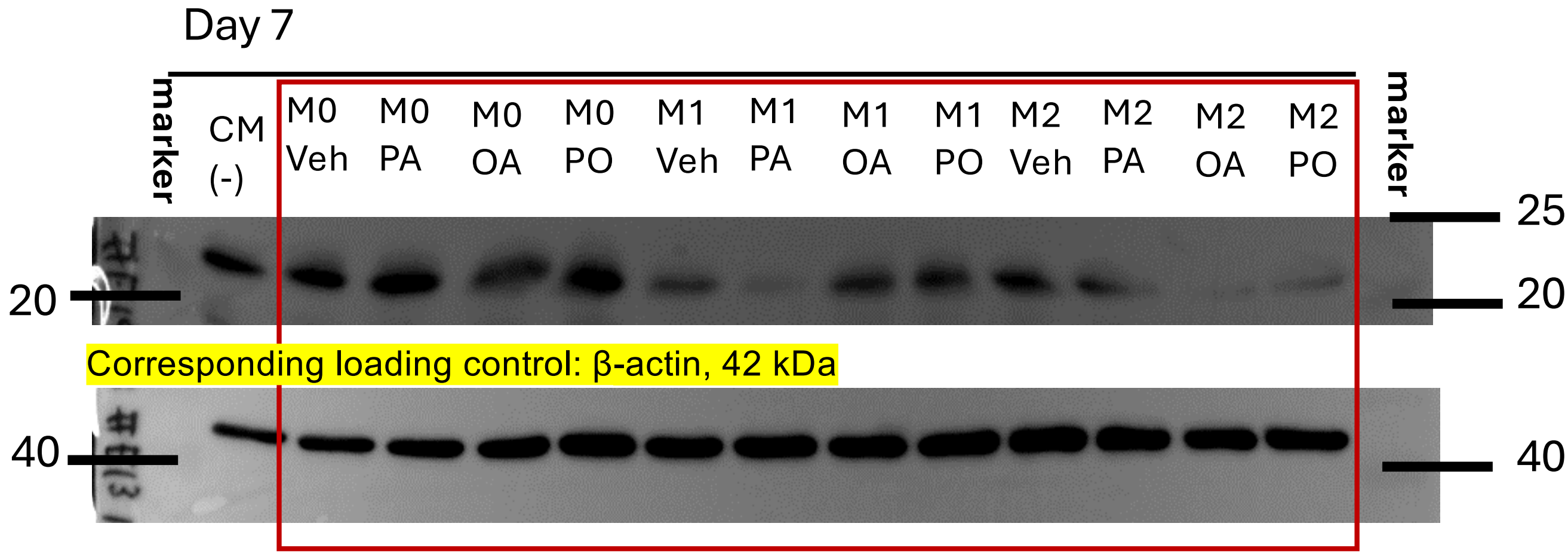

Supplement: Supplementary file 2 [file PHY2-14-e71082-s002.pdf]
