# Supplementary material for: Fatty acid species differentially regulate macrophage polarization and oxidative stress with secondary effects on macrophage–HSC crosstalk
Source: Physiol Rep. 2026 Sep 10;14(17):e71082. doi: 10.14814/phy2.71082 (PMC13562811; doi:10.14814/phy2.71082)
Supplement: Supplementary file 3 [file PHY2-14-e71082-s003.pdf]

Supplementary File S1.

Original images of membrane sections used for Western blot analysis

Original Western blot images corresponding to the representative blots shown in the main figures are provided. During the original experiments, the membranes were cut horizontally according to the expected molecular-weight ranges of the target proteins before antibody incubation. Therefore, intact images of the full uncut membranes are not available. The images presented here are the complete original images available for each membrane section used for detection and densitometric analysis. The blue boxes indicate the regions used for presentation in the main figures.

More than one type of pre-stained molecular-weight marker was used in these experiments. In general, a single-color pre-stained marker was used on one side of the gel and a three-color pre-stained marker on the other side, although the exact marker used varied among experiments. Consequently, the molecular-weight reference bands visible in the original membrane images differ among blots. We have labeled only those molecular-weight marker positions that are clearly identifiable in each original membrane image. No additional protein staining was performed to visualize the molecular-weight markers before blocking or immunoblotting; the marker positions were determined directly from the visible pre-stained marker bands.

The migration positions of the target proteins and β-actin loading controls are indicated. The corresponding figure and panel numbers are provided for each blot. The same blots were used for densitometric analysis where applicable.

Fig. 1c

Target: iNOS
Expected molecular weight: ~**130 kDa**

Molecular-weight marker

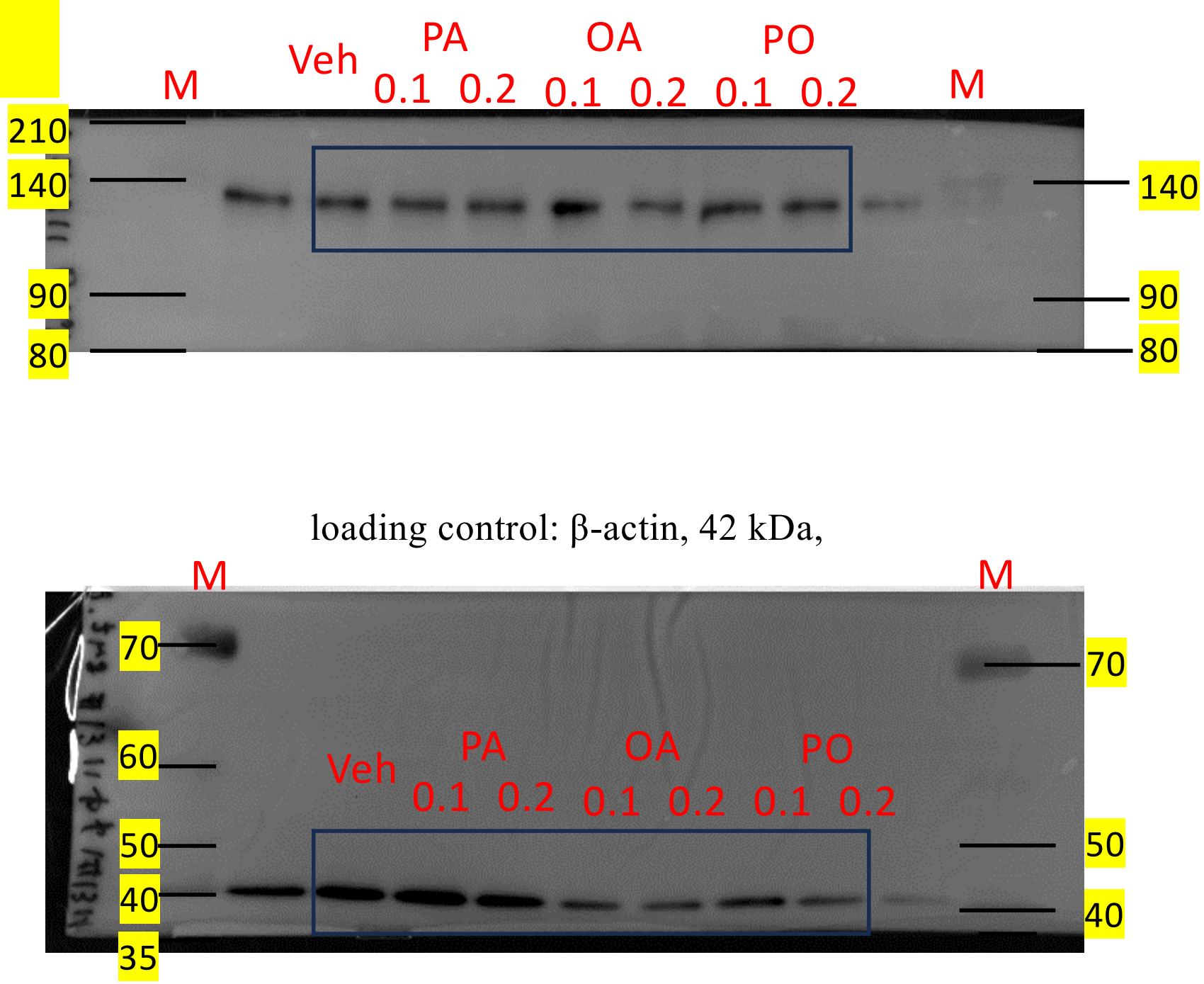

Lane	Sample
M	Molecular-weight marker
1	No BSA/no FA
2	Veh
3	PA 0.1 mM
4	PA 0.2 mM
5	OA 0.1 mM
6	OA 0.2 mM
7	PO 0.1 mM
8	PO 0.2 mM
9	Additional sample, not used in the representative figure
M	Molecular-weight marker

Original images of the available membrane sections corresponding to Fig. 1c. The blue boxes indicate the regions used for presentation in the main figure. The same blots were used for densitometric analysis. "No BSA/no FA" indicates cells maintained under the corresponding experimental condition without BSA vehicle or fatty-acid treatment; "Veh" indicates cells treated with the BSA vehicle used for fatty-acid delivery. Clearly identifiable molecular-weight marker positions are indicated adjacent to the corresponding marker lanes, where applicable, and the migration positions of the target proteins and β-actin loading controls are indicated.

Fig. 1d

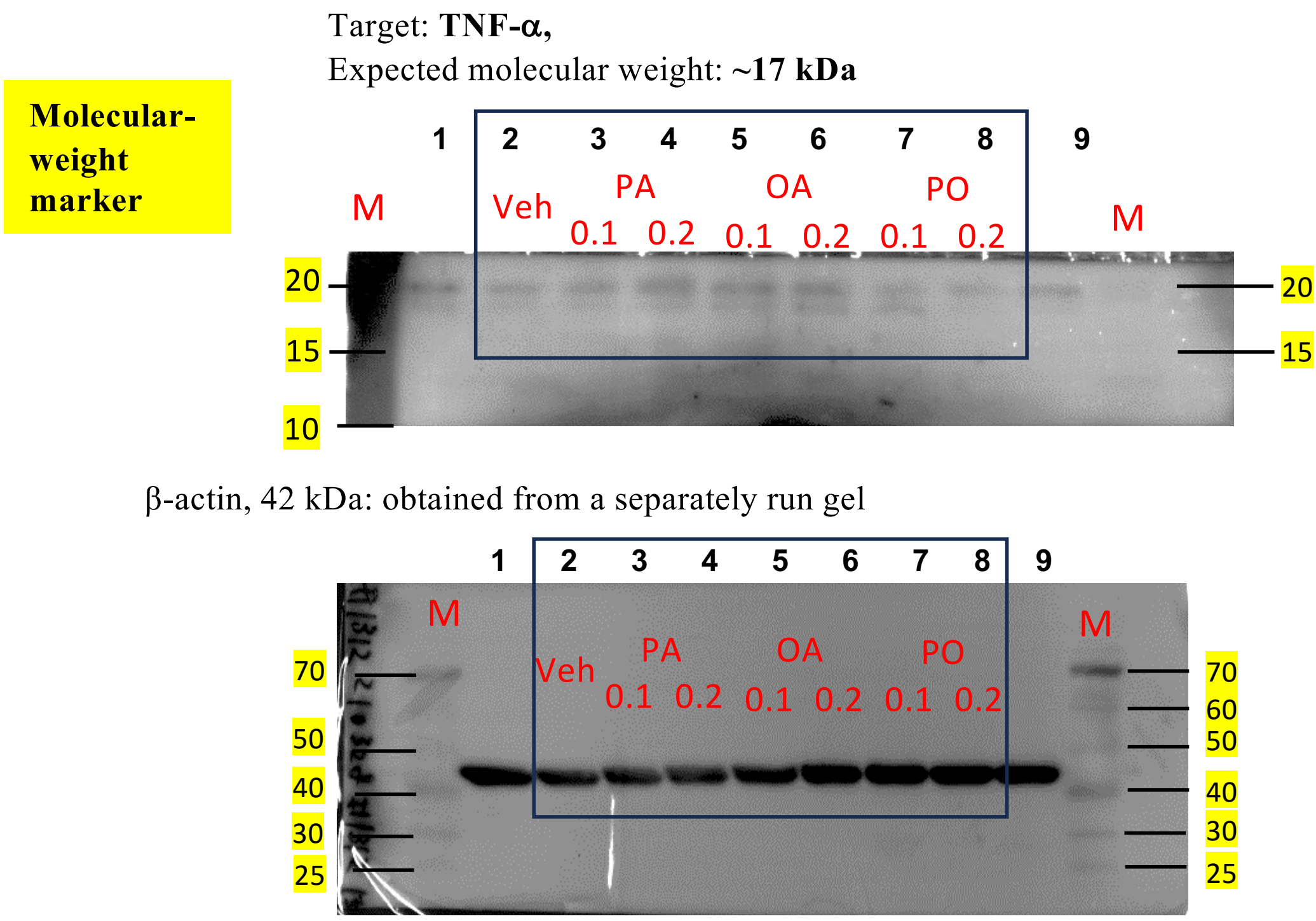

Lane	Sample
M	Molecular-weight marker
1	No BSA/no FA
2	Veh
3	PA 0.1 mM
4	PA 0.2 mM
5	OA 0.1 mM
6	OA 0.2 mM
7	PO 0.1 mM
8	PO 0.2 mM
9	Additional sample, not used in the representative figure
M	Molecular-weight marker

Original images of the available membrane sections corresponding to Fig. 1d. The blue boxes indicate the regions used for presentation in the main figure. The same blots were used for densitometric analysis. The TNF-α and β-actin images were obtained from separate gels and membranes. The β-actin blot was generated using the same sample set and was used for normalization of TNF-α expression. The sample order was identical between the TNF-α and β-actin blots.

Fig. 2d

Target: PPARγ
Expected molecular weight: ~**53 and 58 kDa**

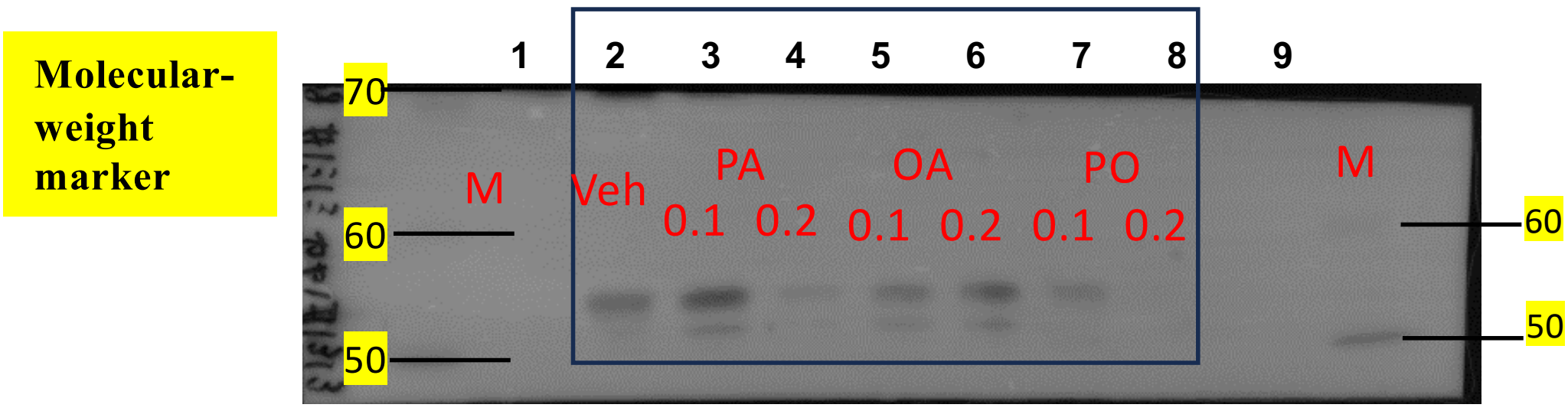

Fig. 2e

Target: MR
Expected molecular weight: ~**190 kDa**

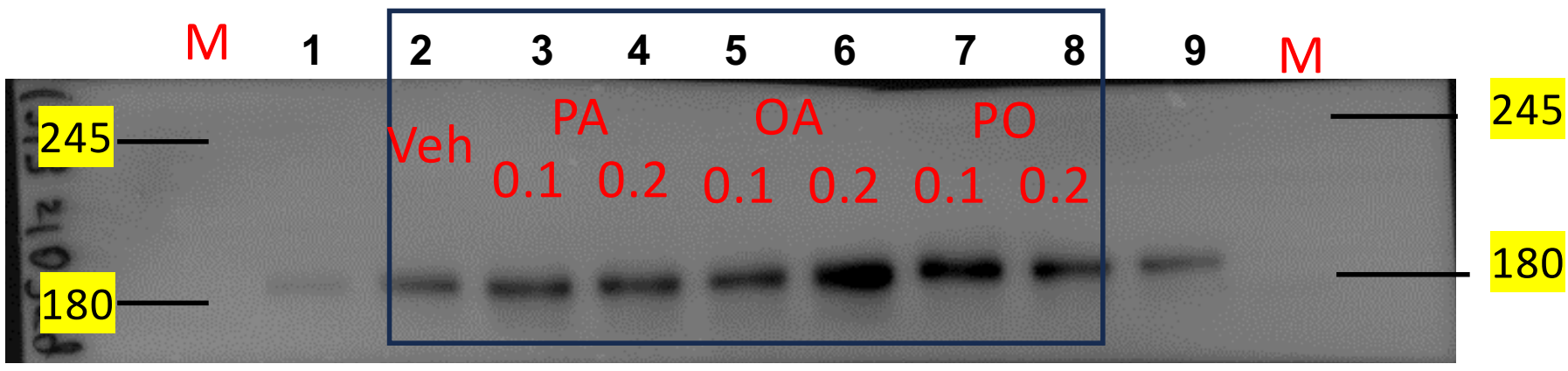

Lane	Sample
M	Molecular-weight marker
1	No BSA/no FA
2	Veh
3	PA 0.1 mM
4	PA 0.2 mM
5	OA 0.1 mM
6	OA 0.2 mM
7	PO 0.1 mM
8	PO 0.2 mM
9	Additional sample, not used in the representative figure
M	Molecular-weight marker

Fig. 2d and 2e

loading control: β-actin, 42 kDa,

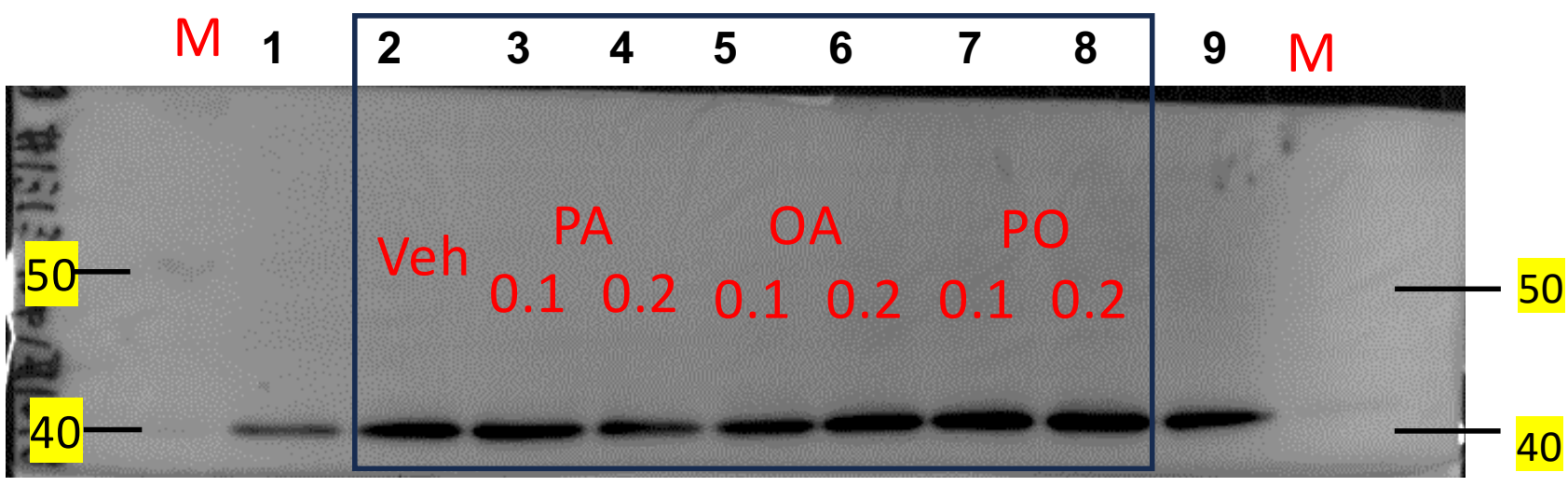

Original images of the available membrane sections corresponding to Fig. 2d and 2e. The blue boxes indicate the regions used for presentation in the main figure. The same blots were used for densitometric analysis.

Fig. 4e

Target: α-SMA
Expected molecular weight: ~**42 kDa**

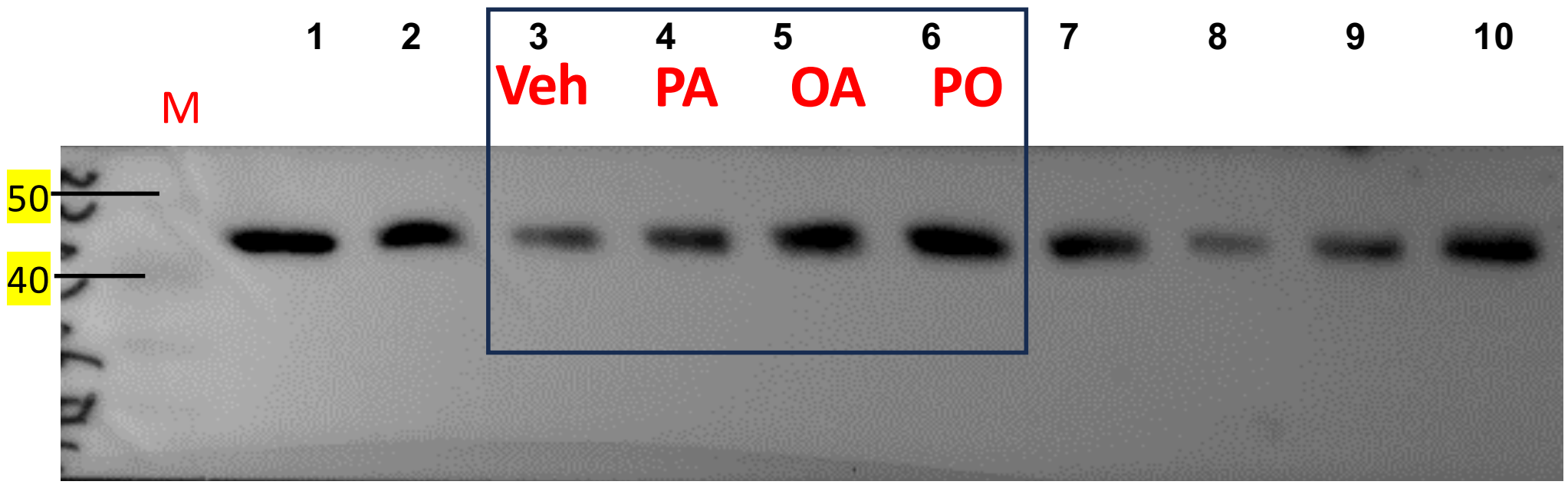

Fig. 4f

Target: cytoglobin,
Expected molecular weight: ~**21 kDa**

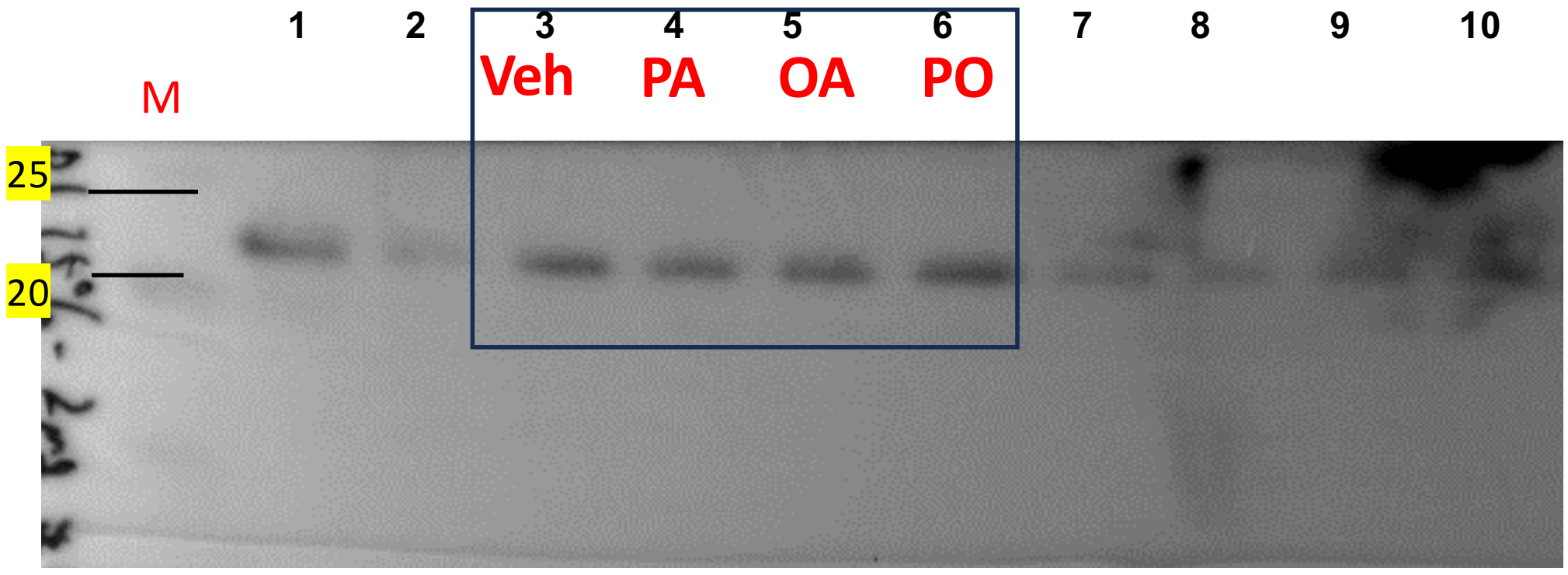

Fig. 4e and 4f

loading control: β-actin, 42 kDa,

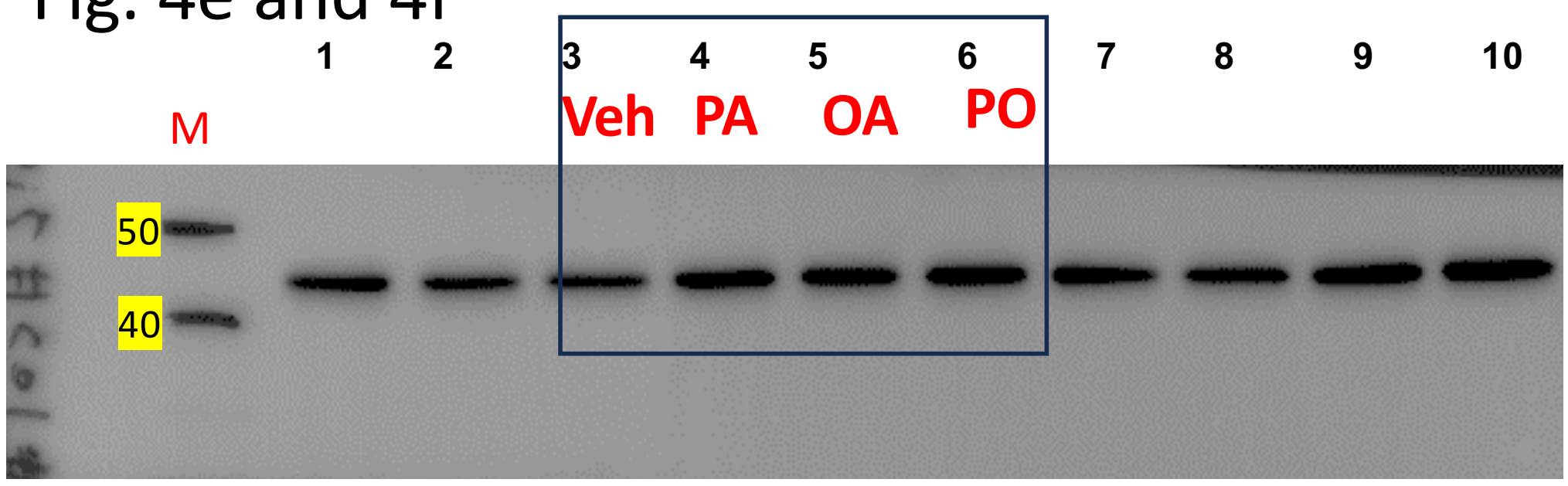

Lane	Sample
M	Molecular-weight marker
1	No BSA/no FA
2	No BSA/no FA
3	Veh
4	PA 0.2 mM
5	OA 0.2 mM
6	PO 0.2 mM
7-10	Additional sample, not used in the representative figure

Original images of the available membrane sections corresponding to Fig. 4e and 4f. The blue boxes indicate the regions used for presentation in the main figure. The same blots were used for densitometric analysis.

Fig. 6a

Target: α-SMA
Expected molecular weight: ~**42 kDa**

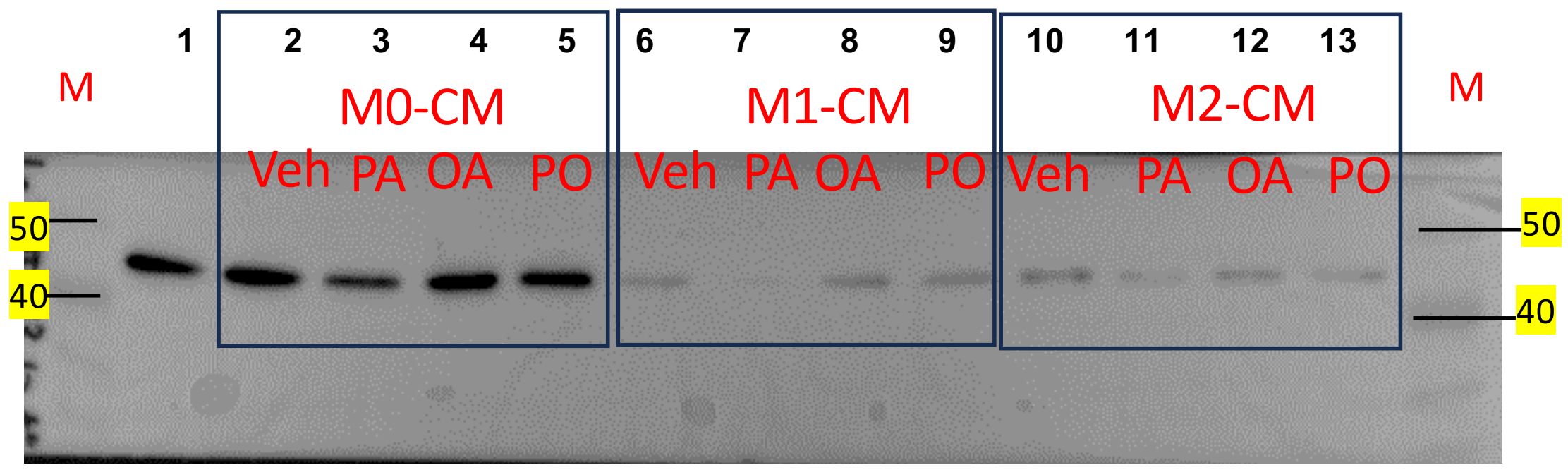

Fig. 6b

Target: cytoglobin,
Expected molecular weight: ~**21 kDa**

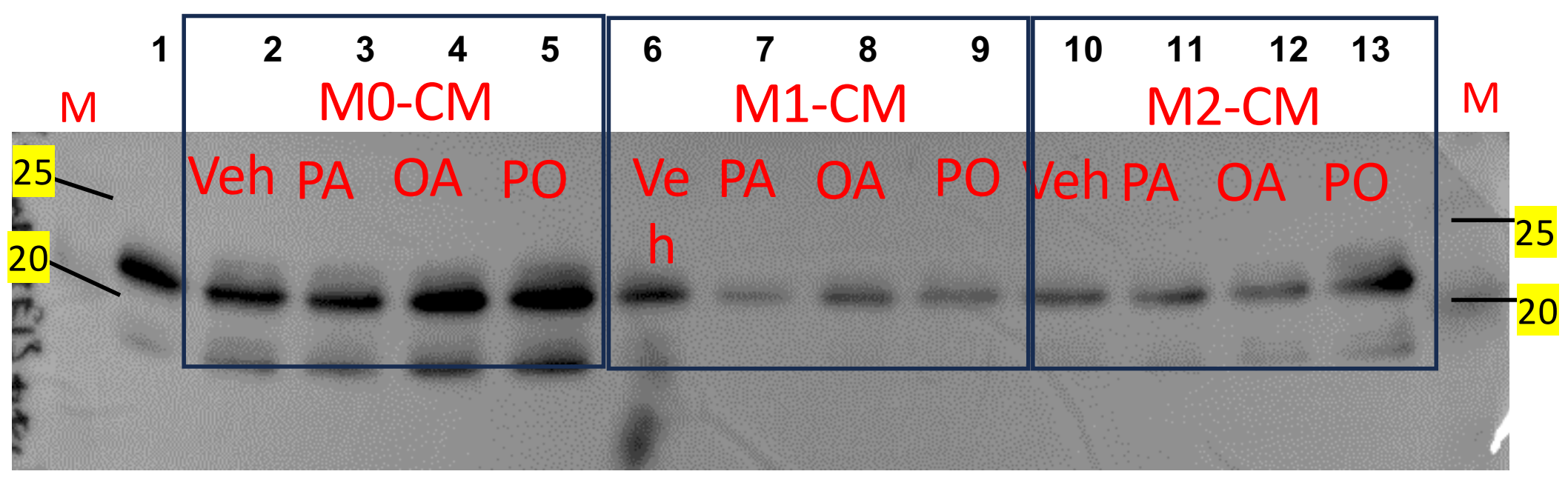

Fig. 6a and 6b

loading control: β-actin, 42 kDa,

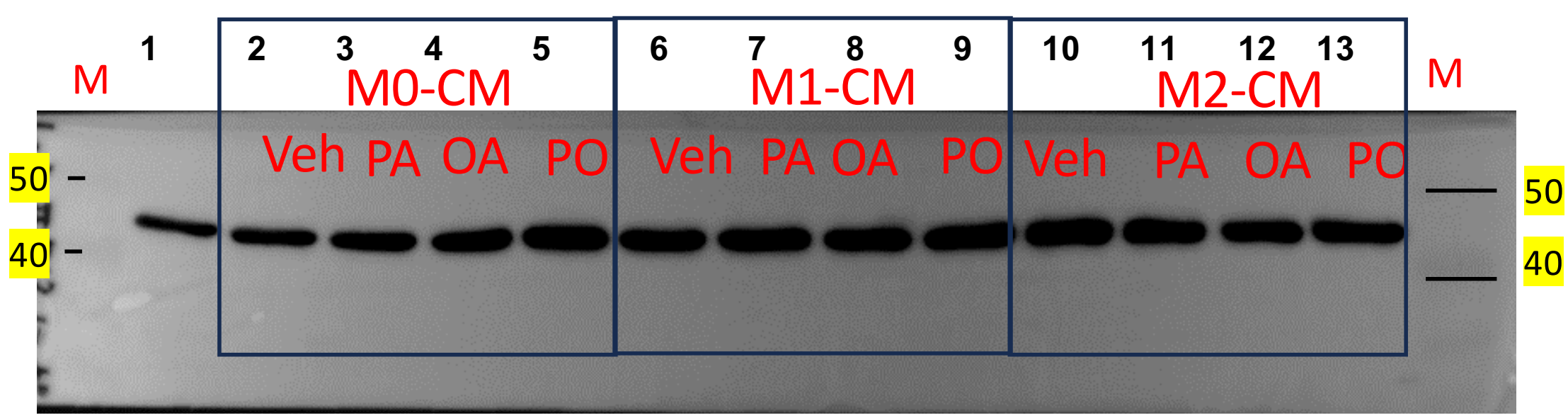

Lane	CM	Sample
M		Molecular-weight marker
1	M	No BSA/no FA
2	M0	Veh
3	M0	PA 0.2 mM
4	M0	OA 0.2 mM
5	M0	PO 0.2 mM
6	M1	Veh
7	M1	PA 0.2 mM
8	M1	OA 0.2 mM
9	M1	PO 0.2 mM
10	M2	Veh
11	M2	PA 0.2 mM
12	M2	OA 0.2 mM
13	M2	PO 0.2 mM
M		Molecular-weight marker

Original images of the available membrane sections corresponding to Fig. 6a and 6b. The blue boxes indicate the regions used for presentation in the main figure. The same blots were used for densitometric analysis.
